# Two Novel *Iboga*-Type and an Oxindole Glucuronide Alkaloid from *Tabernaemontana peduncularis* Disclose Related Biosynthetic Pathways to *Tabernaemontana divaricata*

**DOI:** 10.3390/molecules28186664

**Published:** 2023-09-16

**Authors:** Florian Traxler, Haoqi Zhang, Wiratchanee Mahavorasirikul, Katharina Krivanek, Xiang-Hai Cai, Wichai Aiyakool, Martin Pfeiffer, Lothar Brecker, Johann Schinnerl

**Affiliations:** 1 Department of Organic Chemistry, University of Vienna, Währinger Strasse 38, A-1090 Vienna, Austria; florian.traxler@univie.ac.at (F.T.); katharina.krivanek@gmx.at (K.K.); 2 Vienna Doctoral School in Chemistry, University of Vienna, Währinger Strasse 42, A-1090 Vienna, Austria; 3 Drug Discovery and Development Center, Advanced Science and Technologies, Thammasat University (Rangsit Campus), Pathumthani 12121, Thailand; wiratchanee.m@gmail.com; 4 Thammasat University Research Unit in Cannabis and Herbal Products Innovation, Thammasat University (Rangsit Campus), Pathumthani 12121, Thailand; 5 Department of Botany and Biodiversity Research, University of Vienna, Rennweg 14, A-1030 Vienna, Austria; 6 State Key Laboratory of Phytochemistry and Plant Resources in West China, Kunming Institute of Botany, Chinese Academy of Sciences, Kunming 650201, China; xhcai@mail.kib.ac.cn; 7 Department of Botany, Faculty of Science, Kasetsart University, Bangkok 10900, Thailand; b4804235@gmail.com; 8 Department of Agriculture, Ministry of Agriculture and Cooperatives, Bangkok 10900, Thailand; 9 Institute of Biotechnology and Biochemical Engineering, Graz University of Technology, A-8010 Graz, Austria; martin.pfeiffer@tugraz.at

**Keywords:** Apocynaceae, *Tabernaemontana peduncularis*, *Tabernaemontana divaricata*, javaniside, indole alkaloid, glucuronide alkaloid

## Abstract

Phytochemical investigation of the two *Tabernaemontana* species (Apocynaceae) *T*. *peduncularis* Wall. and *T*. *divaricata* (L.) R.Br. ex Roem. & Schult. indicated closely related biosynthetic pathways leading to lipophilic and hydrophilic alkaloids. In total, 18 specialized metabolites comprising indole-derived alkaloid aglycones, three oxindole-derived alkaloid glycosides, and two iridoid glucosides could be identified in the studied species. Among the alkaloids, the two *Iboga*-type alkaloids 3,7-coronaridine isoindolenine, coronaridine 3,4-iminium and a javaniside derivative bearing a glucuronic acid, named javanuronic acid, could be described by spectroscopic and spectrometric methods for the first time. A docking experiment using alpha-fold was performed to generate a protein model of the enzyme 7-deoxyloganetic acid glucosyl transferase. Performed bioassays exhibited a growth reduction of neonate *Spodoptera littoralis* larvae and reduced cell viability of HepG2 cells of the extracts containing *Iboga* alkaloids, whilst the javaniside derivatives containing hydrophilic fraction did not show any effects. These findings indicate a high flexibility in the formation of bioactive indole alkaloid aglycones by *Tabernaemontana* species and also evidence similar accumulation trends in both species as well as indicate that biosynthetic routes leading to oxindole alkaloids like javanisides are more widespread than reported. Furthermore, the incorporation of the three novel compounds into potential biosynthetic pathways is discussed.

## 1. Introduction

Tryptamine-derived alkaloids occur in a vast structural diversity possessing impressive bioactivities in several plant groups. Most of the known derivatives originate from the plant families Rubiaceae [1] and Apocynaceae [2]. Species of the genus *Tabernaemontana* (99 species circumtropical; nine species in Thailand) (Apocynaceae) [3] are well-known for their accumulation of highly bioactive strictosidine-derived lipophilic alkaloids with complex chemical structures such as *Iboga*-type alkaloids [4,5,6]. In addition to our recent work dealing with tryptamine-derived alkaloids of rubiaceous species [7,8,9], we analyzed methanolic extracts obtained from the leaves and stem bark of *Tabernaemontana peduncularis* Wall. collected in Thailand. Comparatively, we also investigated the alkaloidal composition of *Tabernaemontana divaricata* (L.) R.Br. ex Roem. & Schult. cultivated in a greenhouse to obtain insights into parallel accumulation patterns. For unambiguous identification, we isolated the most significant compounds present in the HPLC chromatograms and elucidated their chemical structures by NMR and MS. Additionally, we performed *in silico* molecular docking experiment to get insights in the possible biosynthesis of one of the identified compounds. The crude extract of *T*. *peduncularis* was initially tested against the insect pest *Spodoptera littoralis* Boisduval, (Noctuidae), and the anti-proliferative activities of the alkaloidal fractions of *T*. *peduncularis* against hepatocellular carcinoma (HepG2) cell line were assessed. Two hydrophilic oxindole alkaloids, previously undescribed in Apocynaceae, were extracted from the leaves of both plants, highlighting their similar alkaloid accumulation patterns. They were subsequently tested for their cytotoxic activity against CH1/PA-1 (ovarian teratocarcinoma), SW480 (colon carcinoma), and A549 (lung carcinoma) cell lines. 

## 2. Results and Discussion

**Figure 1:**
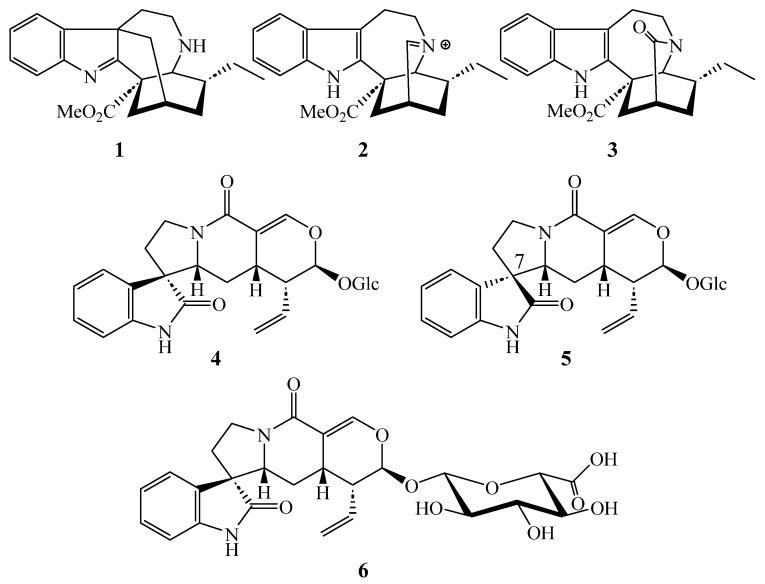
Alkaloids isolated from different parts of *Tabernaemontana peduncularis*.

**Figure 2:**
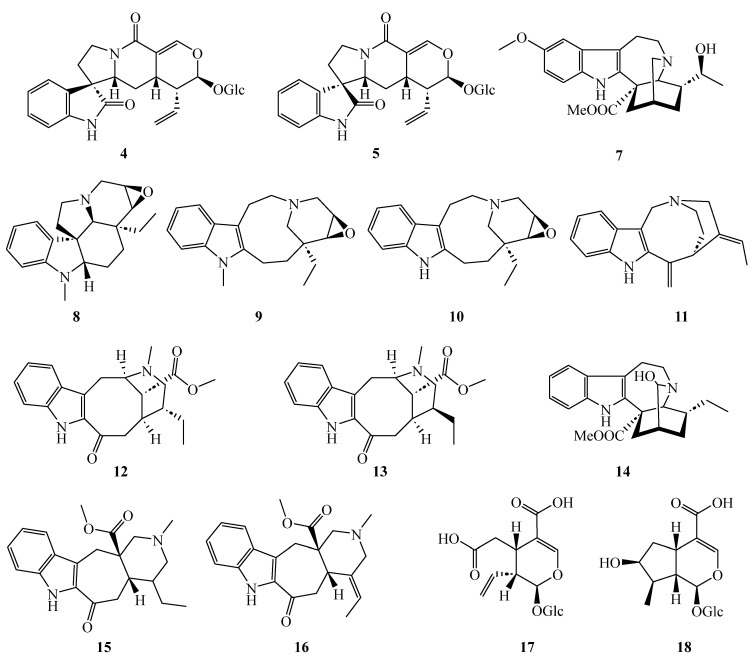
Alkaloids isolated from different parts of *Tabernaemontana divaricata*.

**Figure 3:**
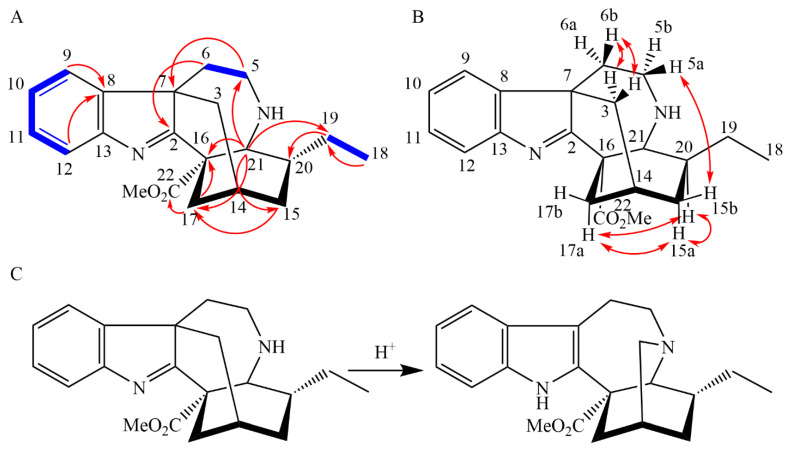
(**A**) Relevant COSY (bold blue line) and HMBC (red arrow) correlations of **1**; (**B**) Relevant NOESY correlations of **1**; (**C**) Rearrangement of **1** under acidic conditions (TFA) to the known alkaloid coronaridine. This transformation may also occur during acid/base extraction methods for alkaloids and is discussed in Section 2.4.

**Figure 4:**
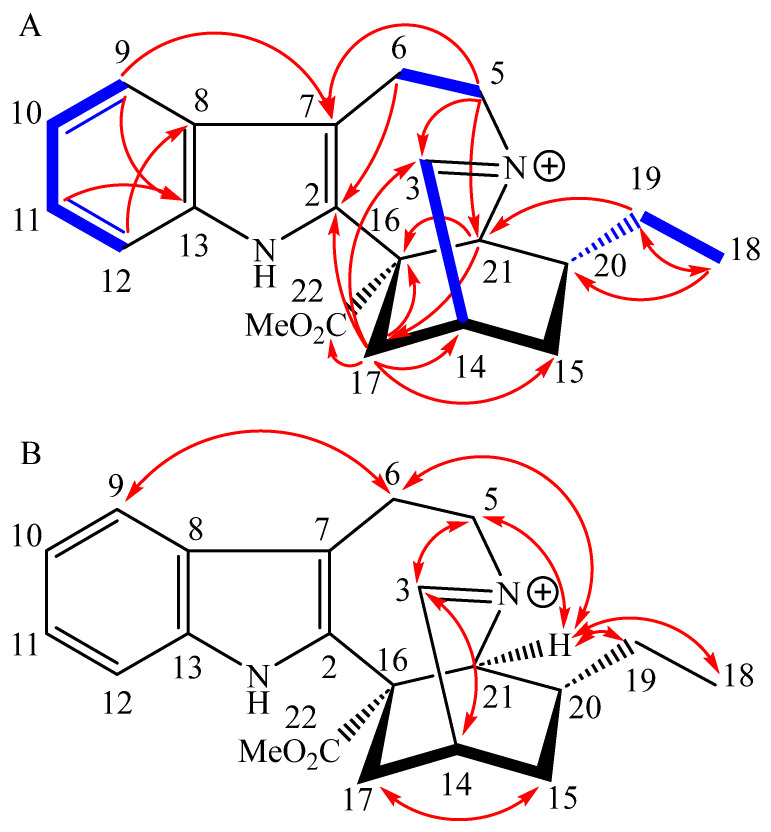
(**A**) Relevant COSY (bold blue line) and HMBC (red arrow) correlations of **2**; (**B**) Relevant NOESY correlations of **2**.

**Figure 5:**
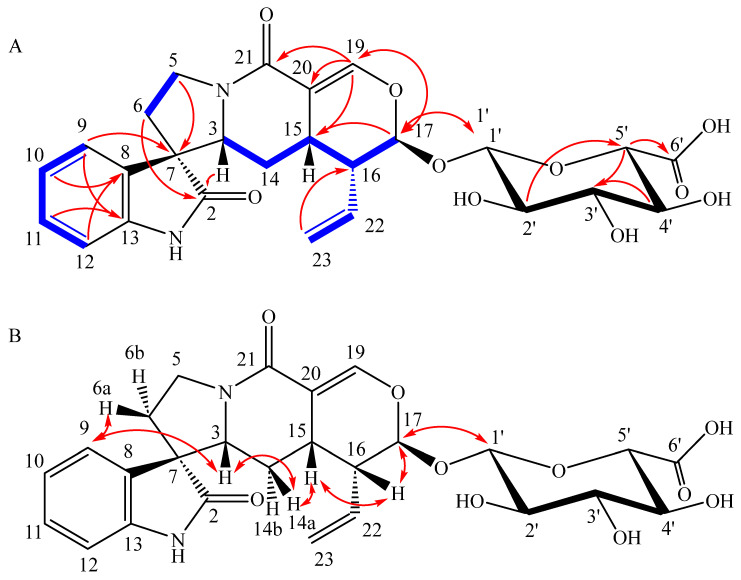
(**A**) Relevant COSY (bold blue line) and HMBC (red arrow) correlations of **4**; (**B**) Relevant NOESY correlations in **4**.

From *T. peduncularis*, we isolated six indole alkaloids (**1**–**6**) (Figure 1) and from *T. divaricata*, 14 indole alkaloids and terpenoid precursors (**4**, **5**, **7**–**18**) (Figure 2). The structures of all isolated compounds were elucidated with data from HR-ESI-MS as well as from 1D (^1^H and ^13^C) and 2D (COSY, TOCSY, NOESY, HSQC, and HMBC) NMR spectroscopic data. The structures, as well as the spectroscopic and spectrometric data of all already known compounds, are in very good agreement with the previously described data. Relative configurations were determined by NMR spectroscopy through proton coupling constants and nuclear Overhauser effects. The absolute configurations were derived by following the biosynthetic pathways starting from known enantiomerically pure precursors and are shown graphically in Figure 1, Figure 2, Figure 3, Figure 4 and Figure 5.

### 2.1. Structure Elucidation of the Novel Compounds ***1***, ***2***, and ***6***

The HR-ESI-MS spectra of 3,7-coronaridine isoindolenine (**1**) showed the major adduct ion at 339.2060 *m*/*z* ([M + H]^+^ calcd 339.2072) from which the sum formula C_21_H_26_N_2_O_2_ was derived. NMR data of this compound (Table 1) showed four aromatic ^1^H resonances (δ_H_ 7.28–7.55 ppm) with the typical coupling patterns and the ^13^C resonances (δ_C_ 121.3–150.8 ppm) corresponding to the A ring of an indole moiety (Figure 3). The resonances corresponding to the indole B ring, however, were altered. A comparison of the peculiar ^13^C signal at δ_C_ 186.0 ppm with the ^13^C NMR chemical shift of C-2 in isoakuammiline (δ_C_ 183.3 ppm) indicates a good accordance with a 3*H*-indole motif [10].

The further moieties of the structure resemble an *Iboga*-type scaffold with a methyl ester moiety (C-22 and C-23 (δ_C_ 172.6 and 52.9 ppm)) connected to the quaternary carbon C-16 that could be determined by its HMBC correlations from C-21 and C-17. Atom C-21 was identified by its chemical shift (δ_C_ 56.1 ppm) and various HMBC correlations to C-5, C-15, C-16, C-17, and C-19. The distinct chemical shift of C-18 (δ_C_ 11.7 ppm) and the corresponding dd ^1^H coupling pattern of CH_3_-18 caused by coupling to H-19 established the ethyl side chain, which could be linked to C-20 by HMBC correlations from CH_3_-18.

TOCSY correlations revealed H-5 and H-6 to be an isolated spin system that could be connected by HMBC correlations from H-5 to the quaternary C-7 and hence the B-ring of the 3*H*-indole motif. This corresponds well with the structure of tryptamine, a precursor for monoterpene indole alkaloids [11,12]. The connection of C-3 to C-7 was indirectly achieved as HMBC correlations were not detected. In principle, the chemical shift of C-3 would allow the connection to N-4, yet this would leave C-7 with an open valence. Additionally, the observed NOESY correlations between H-5a and H-15b are only possible if C-3 is connected to C-7. As this molecule is very rigid, the stereochemistry of C-14 and C-16 was connected and was set as *R* and *S*, respectively, by comparison to the structurally similar molecules coronaridine and heyneanine, which were previously isolated from *T*. *peduncularis* [13]. Based on this stereochemistry, the NOESY correlations between H-20 and H-17a identified the stereocenter at C-20 as *R*.

HR-ESI-MS spectra of coronaridine 3,4-iminium (**2**) showed a positively charged ion at 337.1782 *m*/*z* ([M]^+^ calcd 337.1916—the discrepancy originates from technical problems during the measurement and is discussed in detail in the Supplementary Materials, chapter 2, including Table S1 and Figures S6–S9) from which the sum formula C_21_H_25_N_2_O_2_^+^ was deduced. The NMR data of the compound (Table 1) shows a peculiar C-3 carbon shift of δ_C_ 187.3 ppm with the corresponding proton shift of δ_H_ 10.53 ppm. Such highly deshielded signals in natural products are typically produced by aldehydes or imines, yet this chemical shift is too far upfield for typical aldehydes as well as too far downfield for typical imines [14]. Considering the HMBC correlations of H-5 and H-17 towards C-3 as well as the COSY correlation between H-3 and H-14, an imine is the more likely option (Figure 4). This in turn locates a positive charge on N-4, which can indirectly be observed by the slight downfield shift of C-5 (δ_C_ 58.1 ppm) and C-21 (δ_C_ 62.3 ppm) compared to other *Iboga* alkaloids.

The indole moiety of this alkaloid was identified through its four characteristic aromatic ^1^H resonances (H-9 to H-12, δ_H_ 7.11–7.62 ppm) and the subsequent HMBC correlations towards the quaternary carbons C-7, C-8, and C-13. Atom C-2 was identified through HMBC correlations from H-17 and H-6, completing the B ring of the indole motif. Furthermore, COSY correlations between H-6 and H-5 as well as HMBC correlations between H-5 and C-7, C-3, and C-21 established the ethylamine motif originating from the tryptamine precursor. The ethyl sidechain was revealed through the distinct chemical shift of C-18 (δ_C_ 11.2 ppm) and the dd coupling pattern of H-18 towards H-19. Further COSY couplings between H-19 and H-20 as well as HMBC correlations between H-18 and C-20 allowed for the connection to the iboga ring system. HMBC correlations from H-17 to C-14, C-15 and the quaternary C-16 established the further bonds in this ring system. Additionally, a methyl ester group was identified through its characteristic ^13^C chemical shift (C-22, δ_C_ 169.8 ppm; OMe, δ_C_ 54.0 ppm) and ^1^H singlet signal (OMe, δ_H_ 3.85 ppm). This methyl ester group was connected to C-16 via HMBC correlations from H-17 to C-22.

As the *Iboga* alkaloid scaffold is comparatively rigid, the relative and absolute configuration of the stereocenters of C-14, C-16, and C-21 were assumed to be the same as in other *Iboga* alkaloids reported earlier for the *Tabernaemontana* species. The stereocenter at C-20 would, however, sterically allow two configurations. NOESY correlations proved to be not very pronounced and were thus inconclusive in identifying the relative orientation compared to those of C-21 (Figure 4). However, the proton NMR signal from H-21 shows a singlet coupling pattern. Based on the Karplus equation, this allows for the conclusion that the orientations of H-20 and H-21 must be approximately perpendicular to each other and thus produce torsion angles close to 90°. It is notable that the structure of compound **2** was already proposed by Farrow et al. [15] as an intermediary product in the biosynthesis of coronaridine in *Tabernanthe iboga* Baill. (Apocynaceae). These authors were, however, not able to fully characterize the structure due to its instability, a characteristic we also observed.

The HR-ESI-MS spectra of the javanuronic acid (**6**) showed a not very prominent positively charged molecular ion at 551.1635 *m*/*z* ([M + Na]^+^ calcd 551.1635) as well as a more pronounced negatively charged molecular ion at 527.1681 *m*/*z* ([M − H]^−^ calcd 527.1665). From those, the molecular formula of C_26_H_28_N_2_O_10_ could be derived. A comparison of the NMR data (Table 1) to javaniside **4** (Table S3) revealed large similarities (Figure S29). As such, the oxindole structure could be identified by its typical chemical shifts and coupling pattern of the four protons, H-9 to H-12. The quaternary spiro-carbon C-7 could be identified through HMBC correlations from H-9 to H-5 (Figure 5). COSY correlations between H-5 and H-6 further elucidated the 5-membered ring system which could be concluded by the HMBC correlations from both H-6 and H-3 to the lactam C-2. Continuing from H-3, the large spin system to H-14, H-15, H-16, H-17, H-22, and H-23 could be established by COSY coupling patterns. The vinyl side chain of C-22 and C-23 branching of this spin system was easily identifiable due to the characteristic shift and coupling patterns of the geminal protons at C-23 (δ_C_ 120.5 ppm, δ_H_ 5.16 and 5.22 ppm).

Equally revealing was the characteristic chemical shift of the acetal C-17 (δ_C_ 97.7 ppm). HMBC correlations from H-17 pointed towards another acetal C-1′ (δ_C_ 99.6 ppm), corresponding to the anomeric carbon of the glycoside moiety as well as to the isolated spin H-19. HMBC correlations from H-19 towards the conjugated amide C-21 and corresponding quaternary carbon C-20 as well as C-15 completed the ring system of the aglycon, which corresponds well to those of the aglycon of javaniside **4**. The configuration of the spiro carbon C-7, that is, the difference between javaniside **4** and 7-*epi*-javaniside **5**, was determined through the NOESY correlations. While it may not be immediately obvious from the 2D drawing, the terpene part of the aglycon is approximately planar while the oxindole moiety is perpendicular to it. The configuration at C-7 then determines the direction of the oxindole moiety. NOESY correlations between H-3 and H-9 as well as H-6a and H-9 determine an *S* configuration similar to javaniside **4**. Although H-3 and H-15 did not exhibit a NOESY correlation towards each other, they both correlated to H-14a, determining their syn configuration. Similarly, H-15 and H-16 are syn configurated as demonstrated by their NOESY correlations.

The relative stereochemistry of position 17, however, was more difficult to determine, as the NOESY correlation between H-16 and H-17 does not allow for any conclusion. Based on the biosynthetic precursor secologanine, the same relative orientation as in javaniside (**4**) should be expected. However, a glycosyl exchange may be possible here (see Section 2.3), which could have led to an inversion of position 17. In the aglycon of javaniside (**4**), this is an anti-configuration of H-16 and H-17 with a dihedral angle of approx. 69°, which corresponds well with the observed *J*-coupling of 1.7 Hz. However, the dihedral angle in the syn configuration is approximately 59°, which is a fairly small difference, yet it should result in a slightly higher *J*-coupling constant [16]. Additionally, a syn configuration would place the glycan closer to the vinyl side chain (H-22 and H-23); however, no such NOESY correlations were observed. The aglycon therefore matches the aglycon of javaniside (**4**).

The glycan, however, differed significantly as the expected ^13^C triplet signal of the CH_2_OH (C-6′) group was not present. The mass data, however, already suggested a higher oxidized compound compared to javaniside **4**, as the sum formula contains one more oxygen. Furthermore, the explicitness of the negatively charged [M − H]^−^ ion compared to the positively charged [M + Na]^+^ ion indicated a molecule with a lower p*K*_S_ like a carbonic acid. Indeed, a quaternary carbon at δ_C_ 176.7 (C-6′) can be observed. Additionally, HMBC correlations from H-5′ link it to the glycoside moiety, establishing the presence of a uronic acid. Due to overlapping peaks (H-3′ and H-4′) as well as higher-order couplings, the coupling constants necessary do determine that the stereochemistry had to be simulated by the DAISY package included in TopSpin. The obtained results correspond well with a β-glucuronic acid [17].

### 2.2. Organ Specificity and Identification of the known Compounds

Comparative HPLC profiling using UV diode array detection at 230 nm of the methanolic crude extract obtained from the leaves and stem bark of *T. peduncularis* revealed the exclusive accumulation of the *Iboga*-type alkaloids (**1**–**3**) in the stem bark and the javaniside derivatives (**4**–**6**) in the methanolic leaf extract (Table 2; Figure S81). Leaves of various *Tabernaemontana* species have been proven to be sources for structurally diverse indole alkaloids several times [18,19], but in many studies, pooled aerial parts like twigs and leaves of the *Tabernaemontana* species were used for investigation (e.g., [20]); therefore, a comparison of the data is impossible. Our data suggest some differentiation in the accumulation of compounds, but further studies are required to obtain a closer insight into this issue. A direct comparison of the woody *Tabernaemontana* species to the well-studied herb *Catharanthus roseus* (L.) G.Don. is not expedient due to their different life forms.

Similar to *T*. *peduncularis*, we found the exclusive accumulation of the javaniside epimers **4** [21] and **5** [21] in the methanolic leaf extract of *T. divaricata* together with the known alkaloids voacristine (**7**) [22], mehranine (**8**) [23], voafinidine epoxide (**9**) [24], voaphylline (**10**) [24], and apparicine (**11**) [23] (Table 2). In the methanolic crude extract obtained from the stem bark of *T. divaricata*, we identified apparicine (**11**) [23] as well as tabernaemontanin (**12**) [25], dregamine (**13**) [26], 3-hydroxy coronaridine (**14**), ervatamine (**15**) [27], and 19–20 didehydroervatamine (**16**) [27]; the latter two were also present in the crude methanolic wood extract (Figure S82). From the crude methanolic root extract of *T. divaricata*, the (seco)iridoids secologanoside (**17**) [28] and loganic acid (**18**) [29] could be isolated and identified. The latter, loganic acid (**18**), is an important precursor in the terpenoid pathway towards strictosidine and other monoterpene indole alkaloids [11]. The structural formulae of compounds isolated from *T. peduncularis* and *T. divaricata* are given in Figure 1 and Figure 2. The organ specific distribution is summarized in Table 2.

To the best of our knowledge, the spiro-linked javaniside derivatives are herein described for the first time in Apocynaceae. Javaniside **4** and its epimers were previously described from *Alangium javanicum* (Blume) Wangerin [30,31], *Cornus officinalis* Siebold & Zucc [32]) (both Cornaceae), and Rubiaceae (*Pauridiantha callicarpoides* (Hiern) Bremek. [21], *Palicourea luxurians* (Rusby) Borhidi [33], *Nauclea officinalis* (Pierre ex Pit.) Merr. & Chun ((3*S*,7*R*)-epimer) [34], *Uncaria rhynchophylla* (Miq.) Miq [35]).

### 2.3. Biosynthetic Origins of Javaniside-Type Alkaloids ***4***–***6***

The biosynthetic pathway towards complex monoterpene indole alkaloids found in Apocynaceae is not yet fully described, but a key step is the formation of the precursor strictosidine from tryptamine and secologanin in a Pictet–Spengler reaction. Strictosidine is then deglucosylated to form reactive aldehydes [11,12].

**Figure 6:**
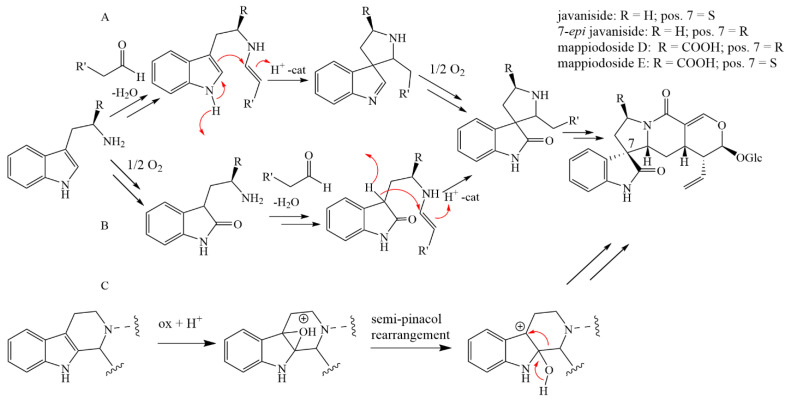
Three possible biosynthetic pathways (**A**–**C**) towards javaniside-like structures. Typically, the Pictet–Spengler reaction is accomplished by a pictet–spenglerase, which leads to the formation of various monoterpene indole alkaloids [12]. In pathway **A**, however, the Pictet–Spengler reaction occurs via a spiroindolenine transition state that is followed by the oxidation of the imine instead of the necessary group migration to form strictosidine [36]. In pathway **B**, the tryptamine is first oxidized to 2-oxotryptamine prior the enamine formation and ring closure. The intermediate from both pathways can then be further transformed to javanisides by saponification and subsequent lactam-ring formation. Cong et al. postulated a similar pathway towards mappiodosides D and E, starting from tryptophan instead of tryptamine and following pathway A [37]. A recently reported alternative pathway **C** would originate from strictosidine or strictosamide. Here, a monoxigenase-catalyzed oxidation followed by a (semi-)pinacol rearrangement (only semipinacol depicted) would lead to the spiro-indole motif. The formation of the lactam present in javanisides may happen before or after this step [38].

The retention of the glucoside moiety in the oxindole structure of javanisides, however, is not consistent with this. This further supports our previously developed hypothesis [33] that the biosynthetic pathway for javanisides may differ from those of other monoterpene indole alkaloids. Hence, the Pictet–Spenglerase responsible for the formation of strictosidine is either able to accept a broader range of educts such as 2-oxotryptamine or the catalysis of the reaction progresses via a spiroindolenine transition state. The latter has been described for similar Pictet–Spengler reactions with certain catalysts [36]. If the reaction then does not proceed with the migration of the amino carbon, the molecule can be oxidized and subsequently form javaniside or its derivatives (Figure 6). Cong et al. [37] postulated a similar biosynthetic pathway for the structurally related mappiodosides D and E in *Mappianthus iodoides* Hand.-Mazz. (Icacinaceae) (Figure 6). Recently, Nguyen et al. identified a cytochrome P450 from *Mitragyna speciosa* Korth. (Rubiaceae) as a potential enzyme responsible for an alternative biosynthetic pathway. This is based on strictosidine or its analogues and induced by monoxigenase-catalyzed oxidation. The terminal oxindole results from a (semi-)pinacol rearrangement (Figure 6). The formed stereocenter can further isomerize by an intramolecular Mannich reaction [38].

Additionally, the presence of the javanuronic acid (**6**) requires a closer look at possible biosynthesis. It is unlikely that such a selective oxidation happened in a non-enzymatic way during the isolation and purification since we employed mild isolation procedures (Figures S1–S5). Furthermore, such artifacts should have been earlier described for isolation procedures of other monoterpene indole alkaloids [1,2,3,4,5,6,8,9,10,13,18,19,20,21,22,23]. It seems possible, but very unlikely, that the glucose was oxidized after the formation of the indole alkaloid, as this would typically require a specialized enzyme for the oxidation of javaniside (**4**) or an alcohol oxidase with a very broad substrate spectrum. Similar enzymatic systems should then be present in other plants as well, leading to a more common prevalence of glucuronated plant metabolites. However, to the best of our knowledge, this is the first described glucuronated plant-derived alkaloid. Some sparse reports of other glucuronated plant metabolites, such as flavonoids [39] and terpenoids [40,41], show that this possibility should not be entirely excluded. In this context, it should also be considered that the javanuronic acid (**6**) was potentially produced by mutualistic organisms living in or on the leaves, as glucuronidation is a common detoxification pathway for many organisms [42,43]. Nonetheless, it is also debatable if the alkaloid would be stable enough to allow for a deglucosylation followed by glucuronidation, since deglucosylation exposes reactive aldehydes similar to the deglucosylation of strictosidine [12].

**Figure 7:**
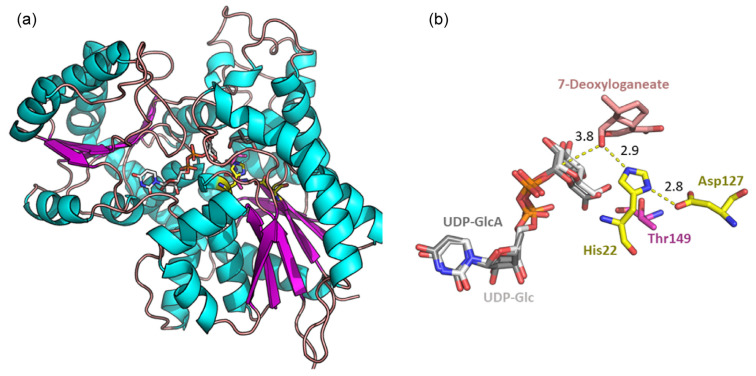
AlphaFold predicted model of 7-deoxyloganetic acid glucosyltransferase originating from *Tabernaemontana elegans*. (**a**) The overall fold of the predicted structure for the glycosyltransferase. Secondary structure elements are highlighted, alpha helixes are in cyan, beta sheets are in purple, and loops are in salmon. (**b**) Overlay of the docking poses for UDP-Glc (white) and UDP-GlcA (grey). Catalytic residues are highlighted in yellow, catalytic key distances are shown as yellow dashes and distances are given in Å, and residues in close proximity with C’’6 hydroxyl are highlighted in purple. The calculated binding energy for the best binding pose of UDP-Glc was 13.21 kcal/mol, while for UDP-GlcA, the binding energy decreased slightly to 13.02 kcal/mol. Both complexes appear catalytically competent for both UDP sugars.

The formation of **6** along the established biosynthesis pathways of monoterpene indole alkaloids would, however, require a more general Pictet–Spenglerase as well as several altered enzymes to accept the different substrates. The crucial step is the glycosylation of 7-deoxyloganetic acid mediated by the enzyme 7-DLGT (7-deoxyloganetic acid glucosyltransferase) [44]. In silico docking studies performed are based on the alpha fold generated structure of the 7-DLGT from *Tabernaemontana elegans* Stapf, the closest relative to *T. peduncularis* with a known protein sequence. The model for the ternary complex shows that an acceptance of UDP-GlcA as an alternative substrate might be possible in principle (Figure 7). However, even if the enzyme in *T. peduncularis* would facilitate this transformation, there are several further transformations required to form strictosidine or javaniside like structures, and all enzymes involved would have to share the relaxed Glc versus GlcA recognition. [44].

It seems possible that hydrophilic alkaloids like javanisides (and hence glucuronated alkaloids as well) were simply overlooked in the past in Apocynaceae or probably other plant taxa as well. Hydrophilic alkaloids, however, would most likely remain in the aqueous phase during the commonly performed acid-base extraction and would therefore be missed. Even glucuronidation could be more common in the biosynthesis of monoterpene indole alkaloids in Apocynaceae or in other plant taxa, as the deglycosylation of strictosidine-glucuronate would lead to the same results regardless.

### 2.4. Acid Induced Rearrangement of Iboga-Type Alkaloid ***1***

Treatment of **1** with approx. 1 eq. of TFA caused a rearrangement to the known alkaloid coronaridine (Figure 3). Coronaridine is an alkaloid commonly found in Apocynaceae and was previously isolated from the stem bark in the only other published study on *T. peduncularis* by Zèches-Hanrot et al. [13] (published under its synonym *Ervatamia peduncularis* (Wall.) King & Gamble). Assuming such a spontaneous, proton-catalyzed rearrangement also occurs in the natural environment, such a transformation could indicate an alternative biosynthetic pathway towards coronaridine than those established in related plants [15]. However, it is also possible that **1** was formed from coronaridine by the reversed rearrangement. The latter assumption is supported by the co-occurrence of **2**, which was identified as an intermediate product in the biosynthesis of coronaridine [15]. Regardless of the biosynthetic pathway towards **1**, it stands to reason that similar reactions under oxidative conditions could also lead to the 7-hydroxy indolenine alkaloids described by Zèches-Hanrot et al. in *T. peduncularis* (after Soxhlet extraction and acid/base purification methods) [13]. Nevertheless, this illustrates the necessity to carefully choose mild isolation procedures, as such acid-catalyzed rearrangements could occur during traditional acid/base isolation procedures.

### 2.5. Evaluation of Cell Viability from Crude Extracts from T. peduncularis

To obtain information on the toxicity of extracts from *T*. *peduncularis*, we assessed initially the anti-proliferative activity of the crude leaf extract, as well as of the chloroform, the ethyl acetate (EtOAc), and the residual water phase obtained from the stem bark extract. For this purpose, we used the 3-(4,5-dimethylthiazol-2-yl)-2,5-diphenyl-2H-tetrazolium bromide (MTT) assay [45]. This test resulted in cell viabilities less than 50% at a concentration of 25 µg mL^−1^. Table 3 shows the growth inhibitory activity (IC_50_) on the HepG2 cell line exposed to each compound for 48 h. Among these samples, the most potent cytotoxicity was found from the chloroform phase of the stem bark extract with an IC_50_ value of 5.30 ± 1.02 µg mL^−1^. The crude methanolic leaf extract and the EtOAc phase showed almost equal cytotoxic effects to HepG2 cells with IC_50_ values of 8.08 ± 1.56 and 8.12 ± 1.19 µg mL^−1^, respectively. Although the isolated compounds could not be tested due their little amounts and the availability of cells, the achieved results provide some impressions about the cytotoxic effects of such alkaloids and confirm the results from other research groups, e.g., [6].

### 2.6. Cytotoxicity of Javaniside (***4***) and **7**-epi-Javaniside (***5***)

It has been previously reported that javaniside **4** has DNA strand scission capabilities in the presence of Cu^2+^ ions [30,31]. We therefore tested the isolated javaniside (**4**) and its epimer 7-*epi*-javaniside (**5**) separately for their cytotoxic activity against the CH1/PA-1 (ovarian teratocarcinoma), SW480 (colon carcinoma), and A549 (lung carcinoma) cell lines. However, the compounds exhibited no significant activity, as the IC_50_ values were higher than 200 μM (the highest value tested in our assays) against these cell lines. This is in accordance with similar compounds isolated from *Uncaria rhynchophylla* (Rubiaceae), differing in hydroxylation at C-11, which did not exhibit any cytotoxic activity [47]. However, Wu et al. [48] noted that substitution in the A ring seems to inhibit DNA strand scission activity, as 11-methoxy javaniside did not exhibit such effects.

The observed cytotoxicity from the crude leaf extracts of *T. peduncularis* (Table 3) can therefore not be explained by the prevalence of the javanisides **4** and **5** in the leaves. It can be expected that javanuronic acid (**6**) similarly shows low or no cytotoxicity, which means that other compounds present in much lower concentrations have to be responsible for the observed cytotoxicity of the crude leaf extract.

### 2.7. Antifeedant Activities of T. peduncularis Extracts on Neonate S. littoralis Larvae

This initially performed assay indicated clear antifeedant activities of the tested crude methanolic extracts obtained from the leaves and stem bark. Especially, the stem bark extract led to a growth inhibition of 82% at a conc of 1.0 mg g^−1^ of food pellet and 87% at 2.5 mg g^−1^, whilst the leaf extract exhibited a growth reduction of 56% at 1.0 mg g^−1^ and 50% at 2.5 mg g^−1^. Nicotine served as a positive control (48.3% growth reduction at 1.0 mg g^−1^). The observed mortality in comparison to the negative control (food powder only) was high with >75% in the stem bark extract, whilst the mortality in the leaf extract was at around 30%. This high mortality also leads to the little differences between the two tested concentrations. Although this experiment was just initially conducted, the results mirror well the results of the cytotoxicity assay (Section 2.5) and the organ-specific distribution pattern of the *Iboga* alkaloids **1**–**3** (Table 3) in the stem bark extract and less active oxindole glycosides **4**–**6** in the leaf extract.

### 2.8. Docking Experiment

The known 7-deoxyloganetic acid glucosyl transferase from *Catharanthus roseus* (Uni Prot. ID: U5NH37) was used as a search model in a pBlast against the genus *Tabernaemontana*. The best homologue found with a sequence identity of 90% was the 7DLGT of *Tabernaemontana elegans* Stapf (NCBI Accession code: AGX93070.1). Alpha fold was used to generate a protein model used in docking runs [49]. Molecular docking was performed with AutoDock VINA implemented in Yasara v. 18.2.7 [50]. The ligands were generated using the Grade web3 server [51]. In a simulation cell of 10 Å × 10 Å × 10 Å was placed the presumed active site, and 100 docking runs with a fully flexible ligand were performed. The results were clustered by an RMSD of 3 Å. Visual inspection of the docking solutions together with the evaluation of the scoring function were used to select the best pose (Figure 7).

## 3. Experimental Section

### 3.1. HPLC and Chromatographic Conditions

HPLC analyses were performed on an Agilent 1100 series with UV-diode array detection using a Hypersil BDS-C18 (250 × 4.6 mm, 5 μm grain size) column at a flow rate of 1.0 mL min^−1^ and an inj. volume of 10 µL. An aqueous solution containing 10 mM ammonium acetate (A) and methanol (MeOH) (B) were used as eluents, and the following gradient was used: from 40–90% B in A within 12 min, from 90–100% B in A within 0.1 min, and 100% B was kept for 5.9 min. The wavelength of detection was set at 230 nm (reference WL, 360 nm). The reported UV-Vis absorption maxima were derived from the HPLC measurements. MPLC separations were performed over silica gel 60 columns (40–63 µm in particle size, Merck Lobar with dimensions of 240-10 or 310-25) employing various eluents. MPLC separations were conducted either on silica gel 60 eluted with various petrol ether (PE)/EtOAc/MeOH mixtures or reversed phase columns (RP-C18) eluted with water/MeOH mixtures. The particle sizes were 40–63 µm and the column had dimensions of 240-10 mm or 310-25 mm. Thin-layer chromatography was performed on silica gel 60 F_254_ plates, with a layer thickness of 0.2 mm (Merck, Rahway, NJ, USA), developed with CHCl_3_/MeOH/H_2_O 80:20:1 or with the organic phase of *n*-butanol/acetic acid/water 50:10:40, respectively. The stationary phases for CC were either Sephadex LH-20 (GE Healthcare, Chicago, IL, USA) or silica gel 60 (Merck) with a 0.2–0.5 mm or 40–63 μm particle size. For the preparative TLC, silica gel F_254_ plates, with a layer thickness of 0.5 mm (Merck), were used.

### 3.2. NMR Spectroscopy

For the NMR spectroscopic measurements, each compound was dissolved in a deuterated solvent (CD_3_OD or CDCl_3_) (the isolated amounts (1–5 mg) in a 0.6 mL solvent) and transferred into 5 mm high precision NMR sample tubes. NMR spectra were recorded at 298.2 ± 0.1 K on a Bruker AV III 600 spectrometer at 600.25 MHz (^1^H) and 150.93 MHz (^13^C) or a Bruker AV III HD 700 at 700.40 MHz (^1^H) and 176.13 MHz (^13^C), respectively. Spectra were processed with the Topspin 4.01 software. Chemical shifts (δ) are reported in ppm; for ^1^H, this is relative to the residual non-deuterated solvent signals in CD_2_HOD (δ_H_ = 3.31 ppm) and chloroform (δ_H_ = 7.26 ppm), and for ^13^C, this is relative to the solvent signals (methanol-*d*_4_, δ_C_ = 49.0 ppm; CDCl_3_, δ_C_ = 77.0 ppm). To determine the 2D spectra, 128 experiments with 2048 data points each were recorded. After the linear forward prediction (LPfc) of 256 data points in the f_1_ dimension and squared sinusoidal (QSINE) multiplication in both dimensions, they were Fourier transformed to 2D spectra. A simulation of coupling constants was performed with the DAISY (version 3.5.2 (2016)) package included in the Bruker Topspin software package. Coupling constants and proton chemical shifts that could be extracted from the spectra were used as starting points for the simulations; otherwise, reasonable literature values were used. Subsequently, the *J*-coupling constants, chemical shifts, as well as line widths were iterated until the best possible fit, judged by visual evaluation, was achieved. Three dimensional relationships, such as dihedral angles, were calculated using Chem3D 22.0.0 (included in ChemOffice Suite by PerkinElmer), and the energy of the structures was minimized using MMFF94 calculations first.

### 3.3. Mass Spectrometry

HR-ESI-MS spectra were obtained on a maXis UHR ESI-Qq-TOF mass spectrometer (Bruker Daltonics, Bremen, Germany). Samples were dissolved and further diluted in ACN/MeOH/H_2_O in the ratio of 99:99:2 (*v*/*v*/*v*) and directly infused into the ESI source with a syringe pump. The ESI ion source was operated on as follows: capillary voltage—4.0–4.5 kV, nebulizer—0.4 bar (N_2_), dry gas flow—4 L min^−1^ (N_2_), and dry temperature—180 °C. Mass spectra were recorded in the range of *m*/*z* 50–1900 in the positive- and negative-ion mode. The sum formulae of the detected ions were determined using Bruker Compass DataAnalysis 4.1 based on the mass accuracy (Δ*m*/*z* ≤ 5 ppm) and isotopic pattern matching (SmartFormula algorithm).

### 3.4. Plant Material

Plant material from *T. peduncularis* was collected in its natural habitat at Ton Tae Nt. Park (7°17’42.3” N, 99°53’00.4” E) in 2016, Trang province, Thailand and was identified by W. Aiyakool. The corresponding voucher specimen (WU 0099055) was deposited at the Herbarium of the University in Vienna. Plant material from *T. divaricata* was obtained from the Schönbrunn Botanical Garden in Vienna (Austria) in 2020.

### 3.5. Extraction and Isolation

#### 3.5.1. *Tabernaemontana peduncularis*

Ground air-dried leaves (58 g) and stem bark (67 g) were exhaustively extracted separately with MeOH at room temperature (3 × 2 days). The obtained crude extracts were pooled and filtered, and the solvent was removed *in vacuo*. The residue was further partitioned between petrol ether (PE) with 5% ethyl acetate (EtOAc) and water. The aqueous phase was subsequentially washed with chloroform, EtOAc, and *n*-butanol. The alkaloid containing fractions were further processed by various chromatographic techniques. Each separation step was monitored by TLC and HPLC.

Stem bark: A total of 67 g of ground stem bark yielded 2.5 g of brownish residue and 400 mg of the CHCl_3_ phase. The Dragendorff’-positive CHCl_3_ fraction was chromatographed by size exclusion column chromatography (SEC) with Sephadex LH-20 eluted with MeOH. This step afforded 4.9 mg of **1**. Fractions containing Dragendorff’-positive spots with different R_f_ values were pooled and purified by MPLC with mixtures of PE and EtOAc from 90:10 till 70:30. Final purification by prep. TLC eluted with PE/EtOAc 85:15 yielded 3.6 mg of **2** and 4.3 mg of **3** (See also Figure S1).

Leaves: A total of 170 mg of the *n*-butanolic phase, obtained from 2.5 g of crude extract, was separated by SEC over Sephadex LH-20 eluted with MeOH/acetone (70:30) followed by MPLC (RP-18, 5–60% MeOH in H_2_O). The latter step afforded 4 mg of **4** and 3 mg of **5**. A portion of approx. 100 mg of the aqueous phase, which contained impure **6**, was subjected to MPLC (RP-18, 5–40% MeOH in H_2_O) followed by prep. TLC developed in CHCl_3_/MeOH/H_2_O in a ratio of 65:30:5 yielded 2.5 mg of **6** (See also Figure S1).

#### 3.5.2. *Tabernaemontana divaricata*

For all samples, air-dried ground leaves (55 g), roots (21.3 g), wood (130 g), and stem bark (78.4 g) were exhaustively extracted separately with MeOH at room temperature (3 × 2 days). The obtained crude extracts were pooled and filtered, and the solvent was removed in vacuo. The residue was further partitioned between PE with 5% EtOAc and water. The aqueous phase was subsequentially washed with chloroform, EtOAc, and *n*-butanol. The alkaloid containing fractions were further processed by various chromatographic techniques. Each separation step was monitored by TLC and HPLC.

Leaves: A total of 55 g of ground-dried leaves yielded 9.1 g of crude extract, and the subsequently performed liquid–liquid extraction with CHCl_3_ of the extraction suspended in water gave 600 mg lipophilic phase. This obtained lipophilic phase was subjected to column chromatography (CC) over silica gel 60 (0.2–0.5 mm grain size) eluted with mixtures of *n*-heptane, EtOAc, and MeOH with increasing polarities. The fraction 6 (11.7 mg) was further purified by prep. TLC developed in *n*-heptane/EtOAc (80:20). This yielded 8.2 mg of **9**. Fractions 22–29 were merged (18.1 mg) and subjected to SEC over Sephadex LH-20 eluted with MeOH. This step yielded 3.0 mg of **8** and 1.2 mg of **7**. Compound **10** (5.8 mg) was obtained by SEC over Sephadex LH-20 eluted with MeOH from silica gel fractions 8–10 (31.3 mg) (See also Figure S2).

The pooled EtOAc (0.1 g) and *n*-butanol (1.2 g) phases were chromatographed over silica gel 60 (40–60 µm grain size) eluted with mixtures consisting of chloroform and MeOH. The Dragendorff’-positive fractions 7 and 8 were merged (181 mg) and further subjected to SEC over Sephadex LH-20 eluted with MeOH (SEC01). Final purification of **11** (3.2 mg) was achieved by prep. TLC from the merged Dragendorff’-positive fractions (7.7 mg). The fractions 14–28 containing impure **4** and **5** of SEC01 were combined (96 mg) and firstly subjected to SEC over Sephadex LH-20 eluted with MeOH. Compounds **4** and **5** were finally purified by RP-MPLC (5–50% MeOH in water). This yielded 21.0 mg of **4** and 18.0 mg of **5** (See also Figure S2).

Stem bark: A total of 78.4 g of ground stem bark yielded 2.3 g of greenish residue and 0.4 g of brownish residue from the CHCl_3_ phase. The alkaloid containing CHCl_3_ fractions was chromatographed in two batches by SEC over Sephadex LH-20 eluted with MeOH (25 and 27 fractions, respectively). Pooling of samples 22 and 23 from the first batch and 26 and 27 from the second yielded 2.0 mg of **11**. Prep. TLC (70:25:5 PE/EtOAc/MeOH) from the pooled fractions 10 (first batch), 11, and 12 (second batch) yielded 11.9 mg of impure **12**. Pooling of fractions (14–16 of the first batch and 16–20 of the second batch) and further purification by MPLC (silica gel 60, 5–100% EtOAc in PE) yielded 3.5 mg of **12**. Further purification of the combined fractions 28–32 from the previous MPLC with prep. TLC (90:7.5:2.5 *n*-heptane/EtOAc/MeOH) yielded 10.1 mg of **14**. Likewise, fractions 66–69 were combined and purified by prep. TLC (70:25:5 *n*-heptane/EtOAc/MeOH) which yielded 4.5 mg of **13** (See also Figure S3).

Wood: Peeled and dried wood (130 g) were ground and extracted with MeOH in the same manner as described above. From the brownish viscous residue (ca., 5 g), 0.7 g of the CHCl_3_ phase could be obtained. Approximately 300 mg of this fraction was further chromatographed over Sephadex LH-20 eluted with MeOH. This yielded 71.5 mg of impure **15** and **16**. This fraction was further refined by SEC over Sephadex LH-20 eluted with acetone. This step led to 20.8 mg of impure **15** and **16**. The final purification was achieved by prep. TLC developed in acetone/MeOH (99.5: 0.5). This step yielded 1.2 mg of **15** and 0.9 mg of **16** (See also Figure S4).

Roots: A total of 21.3 g of dried roots afforded 1.9 g of crude methanolic extract and 0.8 g of the aqueous phase after liquid–liquid extraction. This aqueous phase was suspended in MeOH and centrifuged at 12,500 rpm for 5 min. The supernatant was first subjected to the SEC of Sephadex LH-20 eluted with MeOH, and the fractions containing **17** and **18** were pooled (300 mg) and subjected to RP-MPLC eluted with mixtures consisting of H_2_O and MeOH from 98:2 to 50:50. This step yielded 2.0 mg of **17** and 6.5 mg of **18** (See also Figure S5).

### 3.6. Evaluation of Cell Viability

The hepatocellular carcinoma (HepG2) cell line used in this study was obtained from ATCC (Manassas, VA, USA). The HepG2 cells were cultured in an EMEM medium (ATCC, MD, USA) and were supplemented at 10% with a fetal bovine serum (Gibco) and streptomycin plus penicillin (100 μg mL^−1^ each), respectively (Sigma Co., Madrid, Spain). The cells were maintained at 37 °C, 95% relative humidity, and with 5% CO_2_ in the atmosphere. All the compounds were evaluated in vitro for their anti-proliferative activity against the hepatocellular carcinoma (HepG2) cell line using the 3-(4,5-dimethylthiazol-2-yl)-2,5-diphenyl-2H-tetrazolium bromide (MTT) assay, as a previously reported technique [45]. Briefly, cells were seeded into 96-well tissue culture plates in an appropriated basal medium containing 10% FBS to a final volume of 100 μL. The cells were subjected to different treatments after 24 h of seeding. For the screening test, cells were then incubated for 48 h with test compounds at a concentration of 25 μg mL^−1^. The MTT solution was subsequently added, and cells were incubated for 3 h. After that, the supernatants were removed, and the precipitated formazan was dissolved by adding 100 μL of DMSO. Absorbance at 570 nm was determined using a microplate reader (Varioskan™ Flash Multimode Reader; Thermo Scientific™, Koto City, Tokyo). HepG2 cells were seeded into 96-well tissue culture plates for 24 h and were then incubated for 48 h with test compounds at nine concentrations from 0–200 μg mL^−1^, with doxorubicin (0–100 μg mL^−1^) as the positive control, or vehicle (DMSO) and continued MTT assay as performed in the screening test.

### 3.7. Cytotoxicity Assay Using SW480, CH1/PA-1, and A549 Cells

#### 3.7.1. Cell Lines and Media

The CH1/PA-1 cells (identified via STR profiling as PA-1 ovarian teratocarcinoma cells by Multiplexion, Heidelberg, Germany) were a gift from Lloyd R. Kelland, CRC Center for Cancer Therapeutics, Institute of Cancer Research, Sutton, UK. The SW480 (human adenocarcinoma of the colon) and A549 (human non-small cell lung cancer) cells were provided by the Institute of Cancer Research, Department of Medicine I, Medical University of Vienna, Austria. All cell culture media, supplements, and assay reagents were purchased from Sigma-Aldrich and plasticware from Starlab, unless noted otherwise. Cells were grown in 75 cm^2^ culture flasks as adherent cultures in a minimum essential medium (MEM) supplemented with 10% heat-inactivated fetal bovine serum (FBS; BioWest), 1 mM sodium pyruvate, 4 mM l-glutamine, and 1% non-essential amino acids (from a 100× ready-to-use stock). Cultures were maintained at 37 °C in a humidified atmosphere containing 5% CO_2_.

#### 3.7.2. Assay Procedure

Subconfluent SW480 (colon carcinoma), CH1/PA-1 (ovarian teratocarcinoma), and A549 (non-small cell lung cancer) cells were harvested by trypsinization for 3–5 min. Supplemented MEM was added to stop trypsinization, and cells were centrifuged for 3 min at 1200 rpm. After the aspiration of the supernatant, the cell pellet was resuspended in supplemented MEM. Afterwards, the CH1/PA-1, SW480, and A549 cells were seeded in 100 µL of aliquots in densities of 1.0 × 10^3^, 2.0 × 10^3^ and 3.0 × 10^3^ cells/well, respectively, in clear flat-bottom 96-well microculture plates. After the incubation of the plates for 24 h, test compounds were dissolved in 100% DMSO, serially diluted in supplemented MEM, and added in triplicates of 100 µL/well, whereupon the concentration of DMSO did not exceed 0.5% *v*/*v*. Plates were incubated for 96 h, and then the medium was replaced with 100 µL/well of an MTT-medium mixture. For this purpose, MTT powder had been dissolved in PBS to a final concentration of 5 mg mL^−1^ and then diluted 1:7 in a supplemented RPMI 1640 medium (supplemented with 10% heat-inactivated FBS and 4 mM of l-glutamine). After 4 h of incubation, the dyeing solution was replaced with 150 µL/well of DMSO and optical densities were measured at 550 nm (with 690 nm as reference) with a microplate reader (BioTek ELx808, Winooski, VT, USA). Interpolated IC_50_ values were averaged from at least three independent experiments.

### 3.8. Antifeedant Assay

This experiment was conducted as previously published [52]. Briefly, 367 mg of freeze-dried food powder containing ground white beans, yeast, ascorbic acid, and ethyl 4-hydroxybenzoate (Sigma-Aldrich, St. Louis, MO, USA) as a preservative was spiked with 2.5 and 1.0 mg g^−1^ of food pellet of the crude methanolic extract. Nicotine served as the positive control and was tested in the conc of 1.0 mg g^−1^, and the unspiked food powder served as the negative control. After evaporation of the solvent (MeOH, 16 h), an aqueous vitamin solution and the antibiotic chloramphenicol were added, and the powder solidified with 1.1 mL of a warm Agar solution (5 g of Agar in 140 mL of dH_2_O). These food pellets were transferred into Petri dishes, and 10 freshly hatched larvae of the cotton leafworm *S*. *littoralis* were placed on top of each pellet. The Petri dishes were kept in an incubator at 26 °C and 90% humidity in darkness. The masses of the survivors were evaluated after 96 h, and the percentage of the gained weight was calculated. This experiment was conducted in triplicate.

### 3.9. Spectroscopic Data of the Isolated Compounds

3,7-Coronaridine isoindolenine (**1**): C_21_H_26_N_2_O_2_; HR-ESI-MS: [M + H]^+^ 339.2060 *m*/*z* (calcd 339.2072) (Figure S10); λ max _(MeOH/H_2_O)_ 226, 286, 292 nm; NMR data are reported in Table 1 and Figures S30–S36.

Coronaridine 3,4-iminium (**2**): C_21_H_25_N_2_O_2_^+^; HR-ESI-MS: [M]^+^ 337.1782 *m*/*z* (calcd 337.1911); a discussion of the discrepancy of 38 ppm due to technical problems can be found in the supporting materials in chapter 2, including Figures S6–S9; λ max _(MeOH/H_2_O)_ 222, 284, 292 nm; NMR data are reported in Table 1 and Figures S37–S43.

3-Oxocoronaridine (**3**): C_21_H_24_N_2_O_3_; HR-ESI-MS: [2M + Na]^+^ 727.3190 *m*/*z* (calcd 727.3471), [3M + Na]^+^ 1079.4837 *m*/*z* (calcd 1079.5258), [M − H]^−^ 351.1759 *m*/*z* (calcd 351.1708); λ max _(MeOH/H_2_O)_ 222, 284, 292 nm; NMR data are reported in Table S2 and Figures S14, S44 and S45.

Javaniside (**4**): C_26_H_30_N_2_O_9_; HR-ESI-MS: [M + H]^+^ 515.2027 *m*/*z* (calcd 515.2029), [M + Na]^+^ 537.1848 *m*/*z* (calcd 537.1849), [2M + Na]^+^ 1051.3789 *m*/*z* (calcd 1051.3800), [M − H]^−^ 513.1881 (calcd 513.1873); λ max _(MeOH/H_2_O)_ 208, 244 nm; NMR data are reported in Table S3 and Figures S15, S46 and S47.

7-*Epi*-javaniside (**5**): C_26_H_30_N_2_O_9_; HR-ESI-MS: [M + H]^+^ 515.2034 *m*/*z* (calcd 515.2029 *m*/*z*), [M + Na]^+^ 537.18150 *m*/*z* (calcd 537.1849), [2M + Na]^+^ 1051.3791 *m*/*z* (calcd 1051.3800), [M − H]^−^ 513.1888 (calcd 513.1873); λ max _(MeOH/H_2_O)_ 208, 244 nm; NMR data are reported in Table S4 and Figures S16, S48 and S49.

Javanuronic acid (**6**): C_26_H_28_N_2_O_10_; HR-ESI-MS: [M + Na]^+^ 551.1635 *m*/*z* (calcd 551.1635), [M − H]^−^ 527.1681 (calcd 527.1665), [M+Na-2H]^−^ 549.1500 (calcd 549.1485) (Figures S10–S12); λ max _(MeOH/H_2_O)_ 208, 244 nm; NMR data are reported in Table 1 and Figures S50–S56.

Voacristine (**7**): C_22_H_28_N_2_O_4_; HR-ESI-MS: [M + H]^+^ 385.2153 *m*/*z* (calcd 385.2127), [M − H]^−^ 383.1969 (calcd 383.1971); λ max _(MeOH/H_2_O)_ 232, 288, 296 nm; NMR data are reported in Table S5 and Figures S17, S57 and S58.

Mehranine (**8**): C_20_H_26_N_2_O; HR-ESI-MS: [M + H]^+^ 311.2137 *m*/*z* (calcd 311.2123); λ max _(MeOH/H_2_O)_ 208, 256, 290 nm; NMR data are reported in Table S6 and Figures S18, S59 and S60.

Voafinidine epoxide (**9**): C_20_H_26_N_2_O; HR-ESI-MS: [M + H]^+^ 311.2118 *m*/*z* (calcd 311.2123), [M + K]^+^ 349.1677 *m*/*z* (calcd 349.1682); λ max _(MeOH/H_2_O)_ 232, 288, 294 nm; NMR data are reported in Table S7 and Figures S19, S61 and S62.

Voaphylline (**10**): C_19_H_24_N_2_O; HR-ESI-MS: [M + H]^+^ 297.1963 *m*/*z* (calcd 297.1967), [M + Na]^+^ 319.1782 *m*/*z* (calcd 319.1786), [M − H]^−^ 295.1809 (calcd 295.1810); λ max _(MeOH/H_2_O)_ 230, 284, 290 nm; NMR data are reported in Table S8 and Figures S20, S63 and S64.

Apparicine (**11**): C_18_H_20_N_2_; HR-ESI-MS: [M + H]^+^ 265.1711 *m*/*z* (calcd 265.1705); λ max _(MeOH/H_2_O)_ 208, 304 nm; NMR data are reported in Table S9 and Figures S21, S65 and S66.

Tabernaemontanin (**12**): C_21_H_26_N_2_O_3_; HR-ESI-MS: [M + H]^+^ 355.2012 *m*/*z* (calcd 355.2021 *m*/*z*), [M + Na]^+^ 377.1832 (calcd 377.1841), [2M + Na]^+^ 731.3773 *m*/*z* (calcd 731.3784), [3M + Na]^+^ 1085.5701 *m*/*z* (calcd 1085.5727), [M − H]^−^ 353.1872 (calcd 353.1865); λ max _(MeOH/H_2_O)_ 210, 240, 316 nm; NMR data are reported in Table S10 and Figures S22, S67 and S68.

Dregamine (**13**): C_21_H_26_N_2_O_3_; HR-ESI-MS: [M + H]^+^ 355.2018 *m*/*z* (calcd 355.2021), [M − H]^−^ 353.1883 (calcd 353.1865); λ max _(MeOH/H_2_O)_ 206, 240, 316 nm; NMR data are reported in Table S11 and Figures S23, S69 and S70.

3-Hydroxy coronaridine (**14**): C_21_H_26_N_2_O_3_; HR-ESI-MS: [M + Na]^+^ 377.1830 *m*/*z* (minor, calcd. 377.1836), [M+H-H_2_O] 337.1912 *m*/*z* (major, elimination, calcd. 337.1916); λ max _(MeOH/H_2_O)_ 224, 274, 284, 292 nm; NMR data are reported in Table S12 and Figures S24, S71 and S72.

Ervatamine (**15**): C_21_H_26_N_2_O_3_; HR-ESI-MS: [M + H]^+^ 355.2021 *m*/*z* (calcd 355.2021), [M − H]^−^ 353.1950 (calcd 353.1865); λ max _(MeOH/H_2_O)_ 208, 238, 312 nm; NMR data are reported in Table S13 and Figures S25, S73 and S74.

19,20-Didehydroervatamine (**16**): C_21_H_24_N_2_O_3_; HR-ESI-MS: [M + H]^+^ 353.1862 *m*/*z* (calcd 353.1865), [M − H]^−^ 351.1716 *m*/*z* (calcd 351.1708); λ max _(MeOH/H_2_O)_ 224, 284, 292 nm; NMR data are reported in Table S14 and Figures S26, S75 and S76.

Secologanoside (**17**): C_16_H_22_O_11_; HR-ESI-MS: [M + Na]^+^ 413.1033 *m*/*z* (calcd 413.1059), [2M + Na]^+^ 803.2168 *m*/*z* (calcd 803.2221), [M − H]^−^ 389.1083 (calcd 389.1083); λ max _(MeOH/H_2_O)_ 228 nm; NMR data are reported in Table S15 and Figures S27, S77 and S78.

Loganic acid (**18**): C_16_H_24_O_10_; HR-ESI-MS: [M + Na]^+^ 399.1240 *m*/*z* (calcd 399.1267), [2M + Na]^+^ 755.2582 *m*/*z* (calcd 755.2636), [3M + Na]^+^ 1151.3928 *m*/*z* (calcd 1151.4005), [M − H]^−^ 375.1291 (calcd 375.1291); λ max _(MeOH/H_2_O)_ 228nm; NMR data are reported in Table S16 and Figures S28, S79 and S80.

## 4. Conclusions

An investigation of *Tabernaemontana peduncularis*, collected in its native habitat, led to the discovery of two coronaridine derivatives (**1** and **2**) and an oxindole alkaloid glucuronide (**6**), which is, to the best of our knowledge, the first described alkaloid bearing a glucuronic acid as a sugar moiety. From the greenhouse-bred *T*. *divaricata*, various known *Iboga* (**7**–**16**) and oxindole alkaloids (**4** and **5**) and two iridoid glcycosides (**17** and **18**) could be identified, indicating similarities in the accomplished biosynthetic routes in both species. *In silico* performed docking experiments regarding the glucuronic acid in compound **6** point towards variations in the glycosyltransferases, which enable the transfer of glucuronic acid to the iridoid unit. In contrast to the spiro-linked alkaloid glycosides, the extracts containing the lipophilic *Iboga* derivatives exhibited notable effects against *S*. *littoralis* and the hepatocellular carcinoma (HepG2) cell line. No cytotoxic effects of the javaniside derivatives **4** and **6** against the human cancer cell lines CH1/PA-1, SW480, and A549 could be observed.

## Figures and Tables

**Table 1:** NMR spectroscopic data of 3,7-coronaridine isoindolenine (**1**), coronaridine 3,4-iminium (**2**) in CDCl_3_, and javanuronic acid (**6**) in CD_3_OD. Positions of the carbon atoms are indicated in Figure 3, Figure 4 and Figure 5. All ^1^H NMR chemical shifts (ppm) are listed together with the relative integral, the multiplicity as well as the coupling constants (*J* in (Hz)). In addition, the ^13^C NMR chemical shifts (ppm) and multiplicities are given.

	1		2		6	
pos	δ_C_ (ppm)	δ_H_ (ppm)	δ_C_ (ppm)	δ_H_ (ppm)	δ_C_ (ppm)	δ_H_ (ppm)
2	186.0, s		132.1, s		181.0, s	
3	50.2, t	3.62 (1H, ddd, *J* = 13.8, 11.3, *J* = 2.3)	187.3, d	10.53 (1H, d, *J* = 4.8)	65.6, d	4.10 (1H, *J* = 11.4, 3.1)
		3.11 (1H, ddd, *J* = 15.1, 4.0, *J* = 1.7)				
5	49.8, t	2.81 (1H, m)	58.1, t	5.29 (1H, m)	45.6, t	4.04 (1H, m)
		2.64 (1H, m)		4.23 (1H, dd, *J* = 12.8, 7.6)		3.76 (1H, dd, *J* = 11.9, 9.9)
6	34.2, t	2.23 (1H, m)	20.1, t	3.48 (2H, m)	33.4, t	2.42 (1H, m)
		2.07 (1H, ddd, *J* = 15.1, 2.7, *J* = 2.1)				2.25 (1H, dd, *J* = 13.0, 7.5)
7	38.0, s		105.9, s		58.0, s	
8	141.6, s		126.5, s		129.5, s	
9	127.3, d	7.28 (1H, d, *J* = 7.6)	118.0, d	7.44 (1H, d, *J* = 7.8)	123.9, d	7.32 (1H, d, *J* = 7.4)
10	129.9 ^a^, d	7.35 (1H, m)	120.0, d	7.11 (1H, dd, *J* = 7.8, 7.5)	123.7, d	7.08 (1H, ddd, *J* = 7.4, 7.2, 0.7)
11	121.7 ^a^, d	7.35 (1H, m)	122.9, d	7.17 (1H, m)	123.9, d	7.26 (1H, ddd, *J* = 7.8, 7.2, 1.0)
12	121.3, d	7.55 (1H, d, *J* = 7.8)	112.4, d	7.62 (1H, *J* = 7.8)	111.0, d	6.92 (1H, d, *J* = 7.8)
13	150.8, s		136.1, s		143.5, s	
14	27.4, d	1.89 (1H, m)	33.7, d	2.21 (1H, m)	27.0, t	1.39 (1H, ddd, *J* = 12.1, 3.9, 3.6)
						1.29 (1H, m)
15	31.8, t	1.75 (1H, m)	29.8, t	2.04 (1H, m)	28.7, d	2.99 (1H, m)
		1.07 (1H, m)		1.11 (1H, m)		
16	59.0, s		55.1, s		44.4, d	2.63 (1H, m)
17	38.4, t	2.94 (1H, m)	35.9, t	2.58 (1H, d, *J* = 14.1)	97.7, d	5.52 (1H, d, *J* = 1.7)
		2.22 (1H, m)		1.87 (1H, m)		
18	11.7, q	0.89 (3H, dd, *J* = 8.5, 7.2)	11.2, q	0.95 (3H, dd, *J* = 7.3, 7.2)		
19	27.1, t	1.53 (1H, m)	27.5, t	1.42 (1H, m)	148.3, d	7.39 (1H, d, *J* = 2.3)
		1.42 (1H, m)				
20	38.2, d	1.37 (1H, m)	33.9, d	2.03 (1H, m)	108.9, s	
21	56.1, d	3.99 (1H, m)	62.3, d	5.07 (1H, s)	166.0, s	
22	172.6, s		169.8, s		133.8, d	5.47 (1H, ddd, *J* = 17.2, 10.0, 7.2)
23					120.5, t	5.22 (1H, dd, *J* = 17.3, 1.6)
						5.16 (1H, dd, *J* = 10.2, 1.8)
OMe	52.9, q	3.69 (3H, s)	54.0, q	3.85 (3H, s)		
1′					99.6, d	4.66 (1H, d, *J* = 8.0)
2′					74.6, d	3.18 (1H, dd, *J* = 8.0, 9.4 *)
3′					73.6, d	3.41 (1H, dd, *J* = 9.4 *, 9.1 *)
4′					75.9, d	3.41 (1H, dd, *J* = 9.1 *, 9.7 *)
5′					77.7, d	3.58 (1H, d, *J* = 9.7 *)
6′					176.7, s	

^a^ Exact assignment of respective peaks was not possible. * Coupling constants determined by simulating/iterating with DAISY.

**Table 2:** Overview about the organ-specific distribution of the identified alkaloids. Note that wood and root materials of *T. peduncularis* were not studied.

Taxon	Leaves	Bark	Wood	Roots
*T. peduncularis*	**4**, **5**, **6**	**1**, **2**, **3**	/	/
*T. divaricata*	**4**, **5**, **7**, **8**, **9**, **10**, **11**	**11**, **12**, **13**, **14**, **15**, **16**	**15**, **16**	**17**, **18**

**Table 3:** In vitro cytotoxicity screening test and cytotoxic IC_50_ values of the crude methanolic leaf extract and fractions obtained from liquid–liquid extraction from the crude methanolic stem bark extract on hepatocellular carcinoma (HepG2) cells. Doxorubicin was used as a positive control. The cell viability is given in % of the positive control, and the IC_50_ values are given in µg mL^−1^.

Sample	Organ	Cell Viability ± SD	IC_50_ ± SD	Cytotoxic Activity
Crude extract	leaves	28.47 ± 2.03	8.08 ± 1.56	high
CHCl_3_ phase	stem bark	20.79 ± 5.92	5.30 ± 1.02	high
EtOAc phase	stem bark	28.48 ± 1.83	8.12 ± 1.19	high
Water phase	stem bark	58.22 ± 10.65	nd	nd
Doxorubicin		nd	2.11 ± 0.13	high

The cytotoxicity against HepG2 cells was categorized according to the U.S. National Cancer Institute (NCI) and the Geran protocol [46]. IC_50_: < 20 µg mL^−1^ (high cytotoxic activity), IC_50_: 20–100 µg mL^−1^ (moderate cytotoxic activity), IC_50_: 101–500 µg mL^−1^ (weak cytotoxic activity), IC_50_ > 500 µg mL^−1^ (no cytotoxic activity). nd = Not determined.

## Data Availability

Data are available from the corresponding authors.

## Supplementary Materials

The following supporting information can be downloaded at: https://www.mdpi.com/article/10.3390/molecules28186664/s1. Isolation schemes, mass spectra of compounds **1**, **2**, and **6**, including a detailed discussion of the mass spectrum of compound **2**, NMR tables of compounds **3**–**5**, **7**–**18**. ^1^H and ^13^C NMR figures of all isolated compounds as well as 2D NMR figures for compounds **1**, **2**, and **6** are also included, as well as the alpha fold predicted model of 7-deoxyloganetic acid glucosyltransferase.

## Sample Availability

Samples of the compounds are available from the authors.
