# Supplementary material for: Two Novel Iboga-Type and an Oxindole Glucuronide Alkaloid from Tabernaemontana peduncularis Disclose Related Biosynthetic Pathways to Tabernaemontana divaricata
Source: Molecules. 2023 Sep 16;28(18):6664. doi: 10.3390/molecules28186664 (PMC10535570; doi:10.3390/molecules28186664)
Supplement: Supplementary file 1 [file molecules-28-06664-s001.zip › molecules-2589864-supplementary.pdf]

# Two Novel Iboga-type and an Oxindole Glucuronide Alkaloid from *Tabernaemontana peduncularis* Disclose Related Biosynthetic Pathways to *Tabernaemontana divaricata*

Supplementary material

Florian Traxler [a,b], Haoqi Zhang [a,b], Wiratchanee Mahavorasirikul [c,d], Katharina Krivanek [a,e], Xianghai Cai [f], Wichai Aiyakool [g,h], Martin Pfeiffer [i], Lothar Brecker [a,*], Johann Schinnerl [e,*]

[a] Department of Organic Chemistry, University of Vienna, Währinger Strasse 38, A-1090 Vienna, Austria.

[b] University of Vienna, Vienna Doctoral School in Chemistry, Währinger Strasse 42, A-1090 Vienna, Austria.

[c] Drug Discovery and Development Center, Advanced Science and Technologies, Thammasat University (Rangsit Campus), Pathumthani 12121, Thailand.

[d] Thammasat University Research Unit in Cannabis and Herbal Products Innovation.

[e] Department of Botany and Biodiversity Research, University of Vienna, Rennweg 14, A-1030 Vienna, Austria.

[f] State Key Laboratory of Phytochemistry and Plant Resources in West China, Kunming Institute of Botany, Chinese Academy of Sciences, Kunming 650201, People's Republic of China.

[g] Department of Botany, Faculty of Science, Kasetsart University, Bangkok 10900, Thailand.

[h] Department of Agriculture, Ministry of Agriculture and Cooperatives, Bangkok 10900, Thailand.

[i] Institute of Biotechnology and Biochemical Engineering, Graz University of Technology, 8010 Graz, Austria.

* Corresponding authors.
lothar.brecker@univie.ac.at (L. Brecker); johann.schinnerl@univie.ac.at (J. Schinnerl)

# Content


1. Isolation schemes ........ 3

2. Discussion of the mass spectrum of **2** ........ 8

3. Mass spectra of **1** and **6** ........ 13

4. NMR Tables ........ 17

5. Comparison of 13C NMR spectra of compounds 4, 5 and 6 ........ 32

6. $^{1}$H and $^{13}$C NMR spectra of isolated compounds ........ 33

7. HPLC profiles of the isolated alkaloids ........ 84

# 1. Isolation schemes

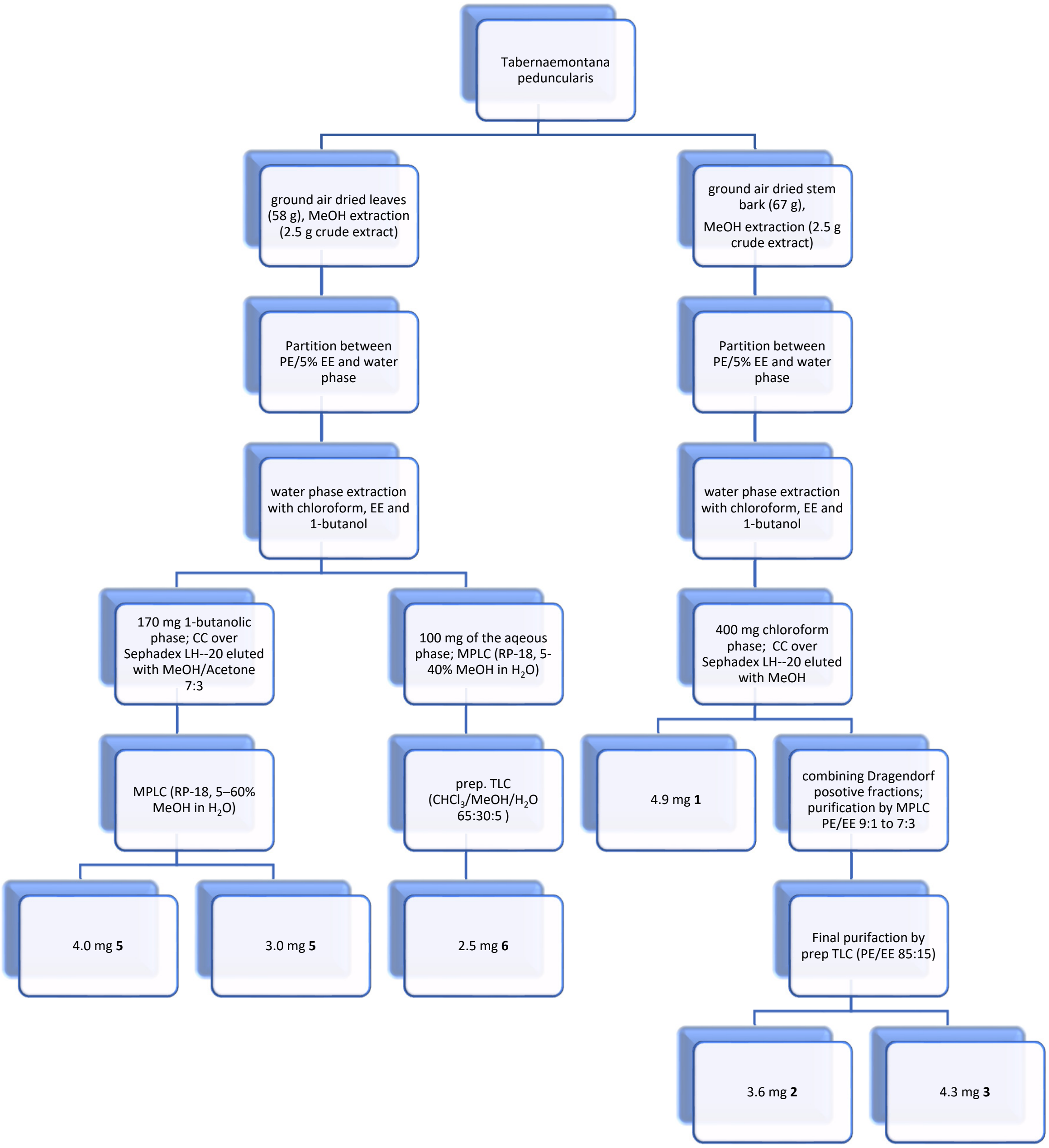


**Figure S1**: Isolation scheme of *Tabernaemontana peduncularis*.

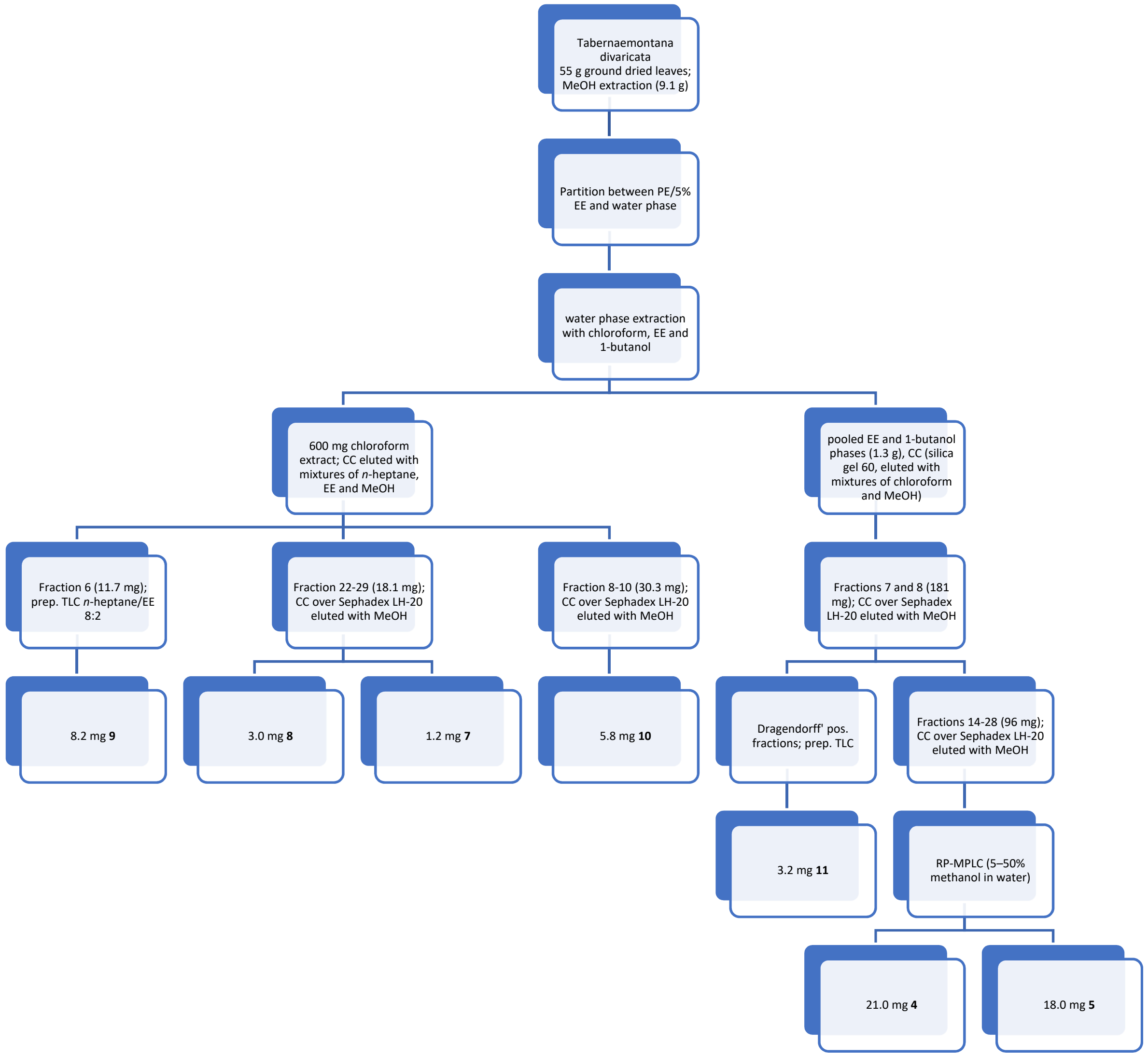


**Figure S2**: Isolation scheme of *Tabernaemontana divaricata* leave material.

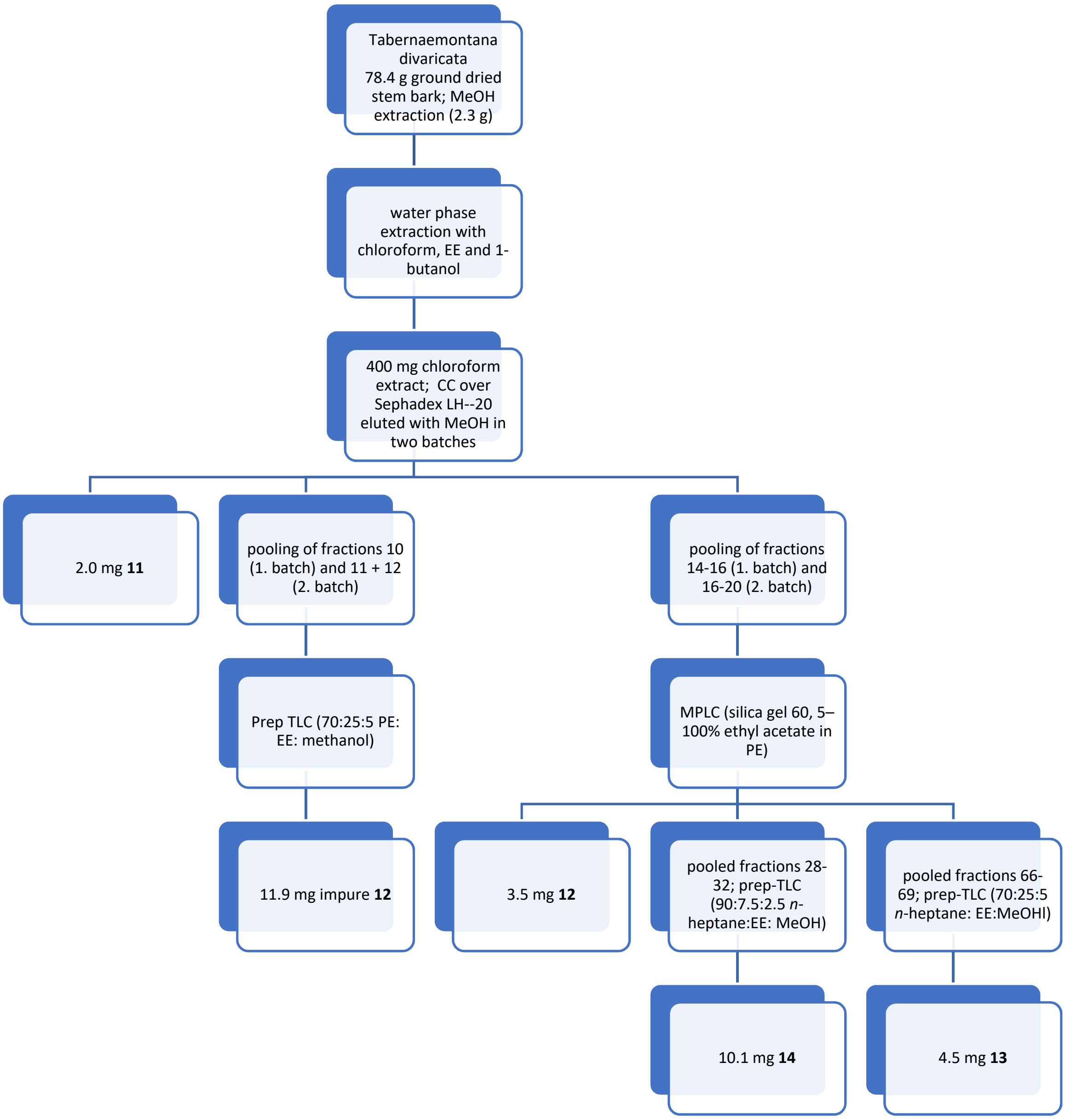


**Figure S3**: Isolation scheme of *Tabernaemontana divaricata* stem bark material.

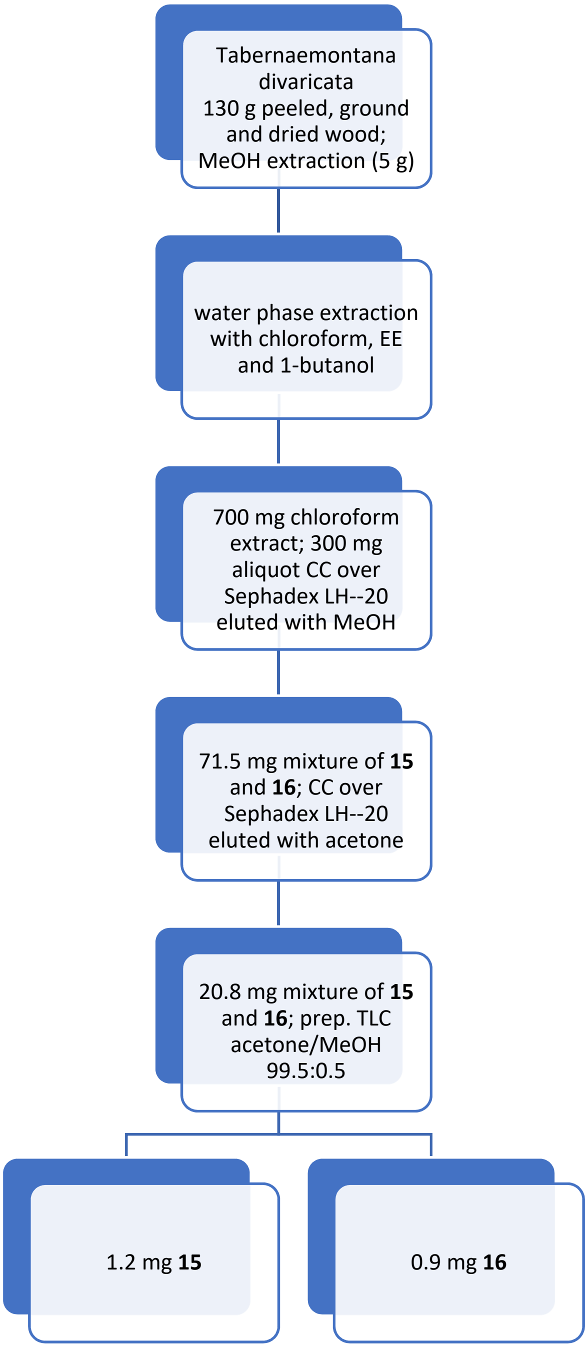


**Figure S4**: Isolation scheme of *Tabernaemontana divaricata* wood material.

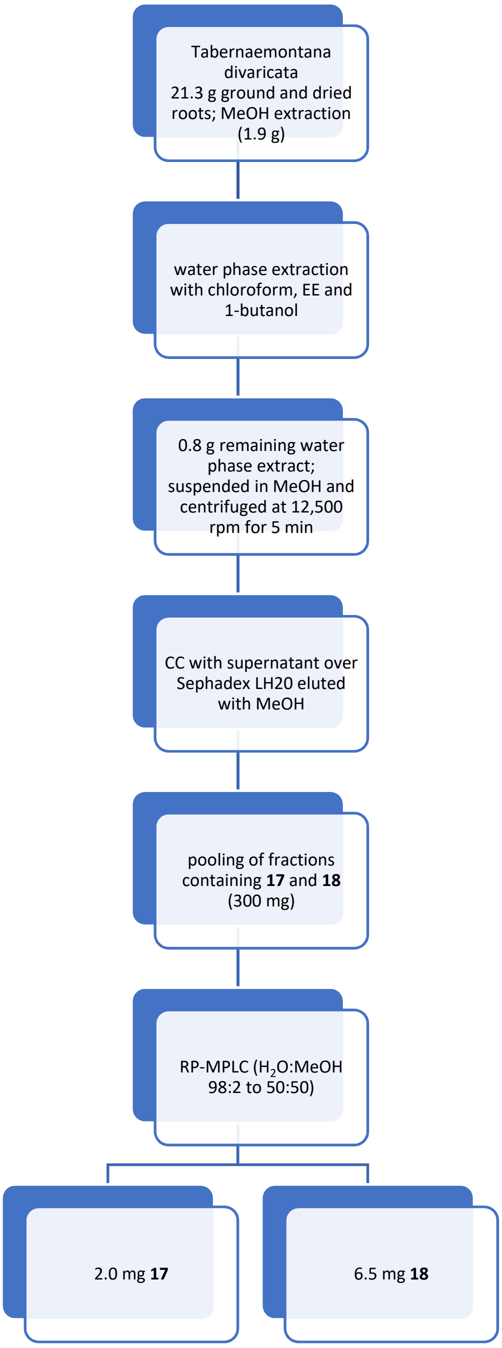


**Figure S5**: Isolation scheme of *Tabernaemontana divaricata* root material

## 2. Discussion of the mass spectrum of **2**

Evidently there is a significant deviation of the measured mass to the calculated of -38.26 ppm (measured 337.1782 *m/z*; $[M]^+$ calculated: 337.1916 *m/z*). Although this is far from the calibration target of our mass facility (<5 ppm) we are fairly certain that the calibration was way off during this measurement. Unfortunately, the sample degraded before we noticed the discrepancy.

The indications that let us believe that our interpretation of the mass data is correct:

- A common impurity (erucamide, a plastic additive) that is regularly observed in our mass spectra is shifted in a similar way (see table S1 and figure S7).
- It was the last run of the day, well after the last calibration run.
- We submitted this as an "unknown" sample, so the mass spectrometry centre at our faculty had no structure proposal to compare their measurement with and catch the bad calibration.
- Many other visible peaks in the spectrum can be explained as degradation products of **2** and exhibit similar mass deviations (table S1)
- Other interpretations of the mass data are not in agreement with the NMR data. The most likely sum formulas to the two most prominent peaks according to the Bruker Compass DataAnalysis 5.1 software are depicted in figure S8.

**Table S1**: Observed peaks, their interpretation and the deviation in the mass spectrum of **2.**

| Explanation | Ion | measured | calculated | deviation [ppm] |
|---|---|---|---|---|
| Observed mass peak | $[M]^+$ | 337.1782 | 337.1911 | -38.26 |
| Addition of $H_2O$ to the Imide (Hydrolysis of imide) | $[M+H_2O]^+$ | 355.1879 | 355.2016 | -38.57 |
| Reduction of Imide to amide | $[M+2H]^+$ | 339.1937 | 339.2067 | -38.32 |
| Addition of methanol (solvent) to the imide | $[M+CH_3OH]^+$ | 369.2031 | 369.2173 | -38.38 |
| Common contaminant: Erucamide | $[C_{22}H_{43}NO+Na]^+$ | 360.3099 | 360.3237 | -38.24 |

# Generic Display Report

**Analysis Info**

| | | | |
|---|---|---|---|
| Analysis Name | E:\Data\MS_MessService\53999000002.d | Acquisition Date | 11/2/2017 4:15:37 PM |
| Method | tune_low_MS_Service_10_17.m | Operator | msc |
| Sample Name | KK-WD02 | Instrument | maXis |
| Auftraggeber/Com | Brecker / Botanik<br>Ergebnis: +/- 5ppm<br>ACN/MeOH + 1%H2O | | |

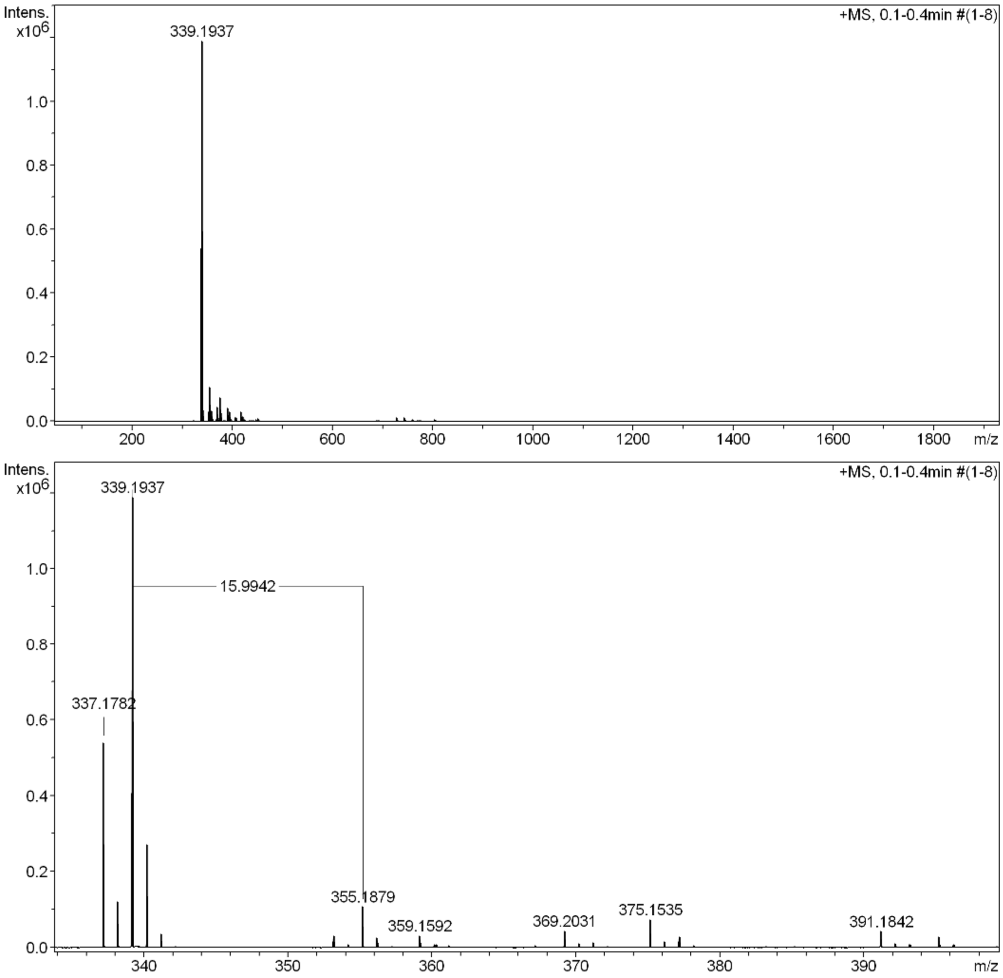


Bruker Compass DataAnalysis 4.0 printed: 11/2/2017 4:22:59 PM Page 1 of 1

**Figure S6**: Mass spectrum of **2**.

# Generic Display Report

**Analysis Info** Acquisition Date 02/11/2017 16:15:37

| | | | |
|---|---|---|---|
| Analysis Name | \\Diskstation\MSC NAS\MSC Data\MS_Service\MAXIS\MS7_MessService_Archiv_2017_490_549\539\53999000002.d | | |
| Method | tune_low_MS_Service_50_57_m | Operator | msc |
| Sample Name | KK-WD02 | Instrument | maXis |
| Comment | Brecker / Botanik<br>Ergebnis: +/- 5ppm<br>ACN/MeOH + 1%H2O | | |

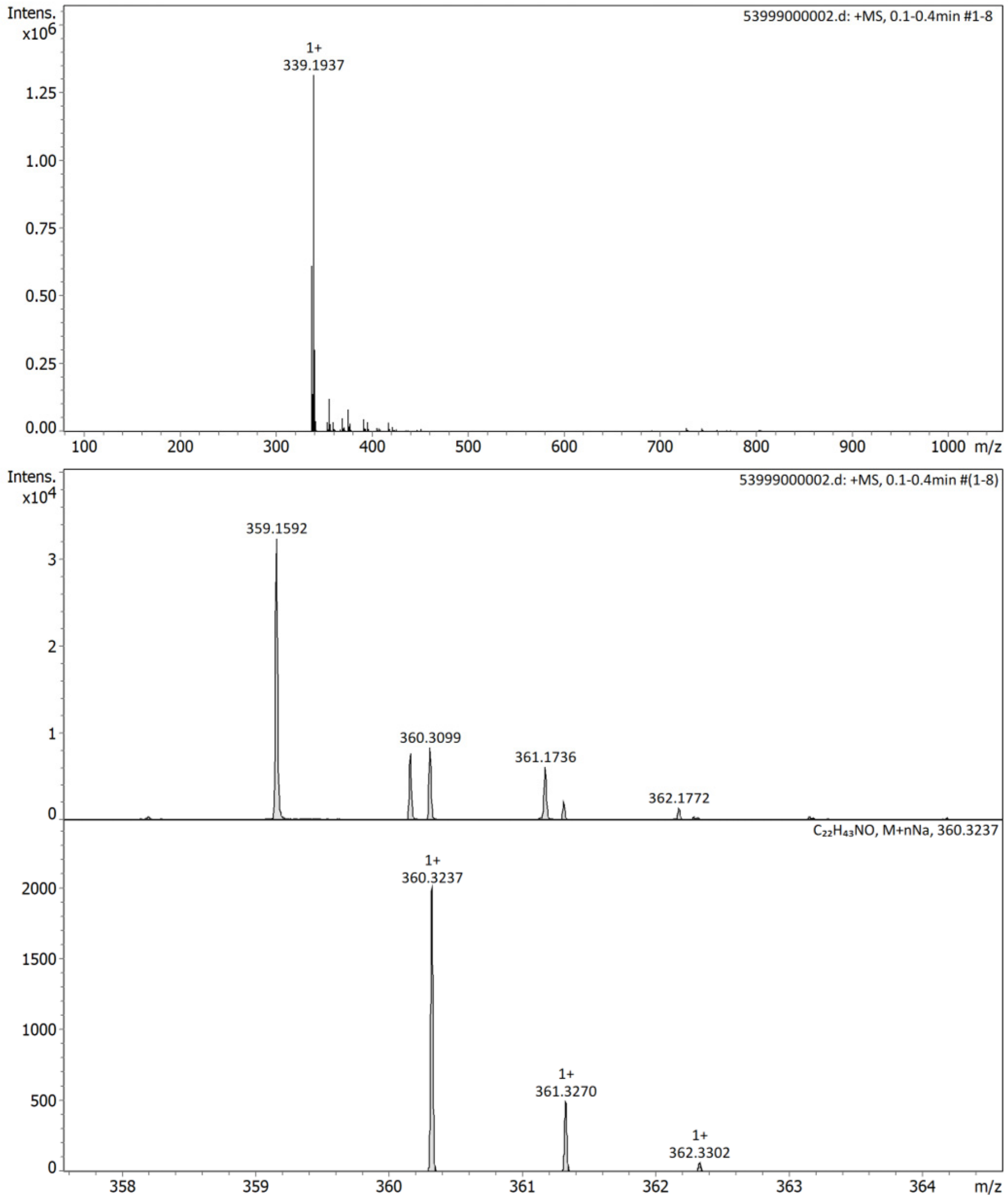


Bruker Compass DataAnalysis 5.1 printed: 16/03/2023 15:54:15 by: admin Page 1 of 1

**Figure S7**: Zoom into the erucosamide (common contaminant) peak (top). The simulation of the erucosamide peak is at the bottom ($[C_{22}H_{43}NO+Na]^+$). Note the similar shift of 38 ppm compared to the peaks of compound **2**.

# Mass Spectrum SmartFormula Report

**Analysis Info**

| | | | |
|---|---|---|---|
| Analysis Name | \\Diskstation\msc nas\MSC Data\MS_Service\MAXIS\MS_MessService_Archiv_2017_490_549\539\53999000002.d | Acquisition Date | 02/11/2017 16:15:37 |
| Method | tune_low_MS_Service_10_17.m | Operator | msc |
| Sample Name | KK-WD02 | Instrument | maXis 255552.00016 |
| Comment | Brecker / Botanik<br>Ergebnis: +/- 5ppm<br>ACN/MeOH + 1%H2O | | |

**Acquisition Parameter**

| | | | | | |
|---|---|---|---|---|---|
| Source Type | ESI | Ion Polarity | Positive | Set Nebulizer | 0.4 Bar |
| Focus | Not active | Set Capillary | 4500 V | Set Dry Heater | 180 Â°C |
| Scan Begin | 50 m/z | Set End Plate Offset | -500 V | Set Dry Gas | 4.0 l/min |
| Scan End | 1900 m/z | n/a | n/a | Set Divert Valve | Source |
| | | Set Corona | 0 nA | Set APCI Heater | 0 Â°C |

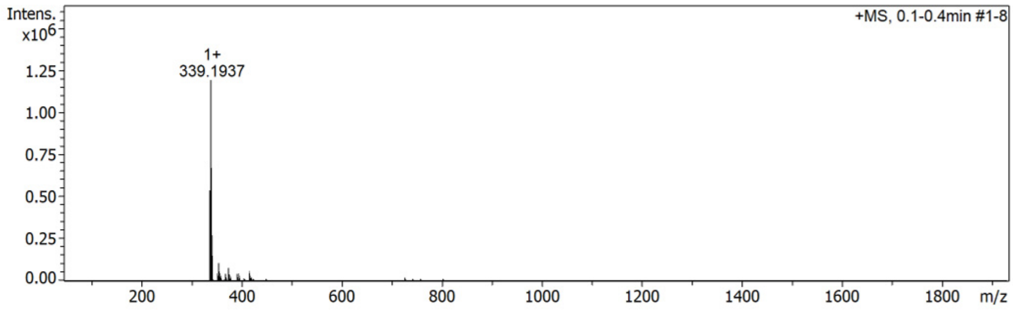


| Meas. m/z | # | Ion Formula | m/z | err [ppm] | mSigma | # mSigma | Score | rdb | eÂ¯ Conf | N-Rule |
|---|---|---|---|---|---|---|---|---|---|---|
| 337.1782 | 1 | C18H21N6O | 337.1771 | -3.1 | 15.0 | 1 | 100.00 | 12.0 | even | ok |
| | 2 | C22H25O3 | 337.1798 | 4.9 | 645.5 | 2 | 0.00 | 11.0 | even | ok |
| | 1 | C18H21N6O | 337.1771 | -3.1 | 15.0 | 1 | 100.00 | 12.0 | even | ok |
| | 2 | C22H25O3 | 337.1798 | 4.9 | 645.5 | 2 | 0.00 | 11.0 | even | ok |
| | 1 | C18H21N6O | 337.1771 | -3.1 | 15.0 | 1 | 100.00 | 12.0 | even | ok |
| | 1 | C6H18N16Na | 337.1793 | 3.2 | 58.4 | 1 | 100.00 | 6.0 | even | ok |
| | 2 | C20H26NaO3 | 337.1774 | -2.2 | 646.5 | 2 | 0.00 | 8.0 | even | ok |
| | 1 | C18H26KN4 | 337.1789 | 2.2 | 48.1 | 1 | 100.00 | 8.0 | even | ok |
| | 2 | C17H30KO4 | 337.1776 | -1.8 | 52.7 | 2 | 93.12 | 3.0 | even | ok |
| 339.1937 | 1 | C18H23N6O | 339.1928 | -2.8 | 6.3 | 1 | 100.00 | 11.0 | even | ok |
| | 1 | C18H23N6O | 339.1928 | -2.8 | 6.3 | 1 | 100.00 | 11.0 | even | ok |
| | 1 | C18H23N6O | 339.1928 | -2.8 | 6.3 | 1 | 100.00 | 11.0 | even | ok |
| | 1 | C20H28NaO3 | 339.1931 | -2.0 | 5.1 | 1 | 100.00 | 7.0 | even | ok |
| | 1 | C18H28KN4 | 339.1946 | 2.4 | 33.8 | 1 | 100.00 | 7.0 | even | ok |
| | 2 | C17H32KO4 | 339.1932 | -1.5 | 39.9 | 2 | 99.33 | 2.0 | even | ok |

53999000002.d

Bruker Compass DataAnalysis 5.1 printed: 17/03/2023 11:36:35 by: admin Page 1 of 1

**Figure S8**: Predicted sum formulas of the most prominent peaks by the Bruker Compass DataAnalysis 5.1 Software. Prediction was performed with the following Ions $[M]^+$, $[M+H]^+$, $[M+NH_3]^+$, $[M+Na]^+$, $[M+K]^+$, CHNO as possible elements and at least 5 carbons.

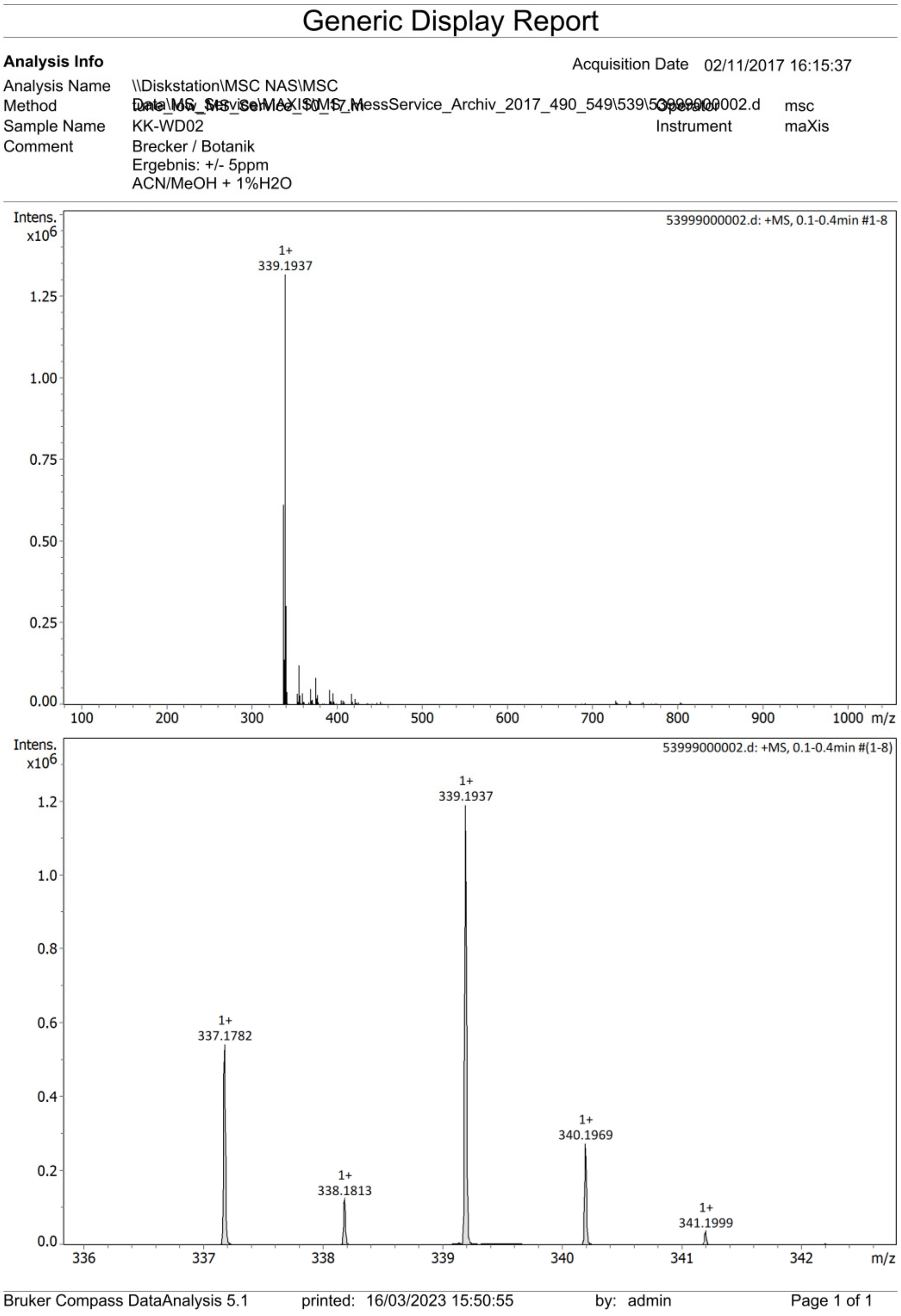


**Figure S9**: Zoom into the region of the [M]+ peak of **2** (measured 337.1782 m/z; $[M]^+$ calculated: 337.1916 m/z).

# 3. Mass spectra of 1 and 6

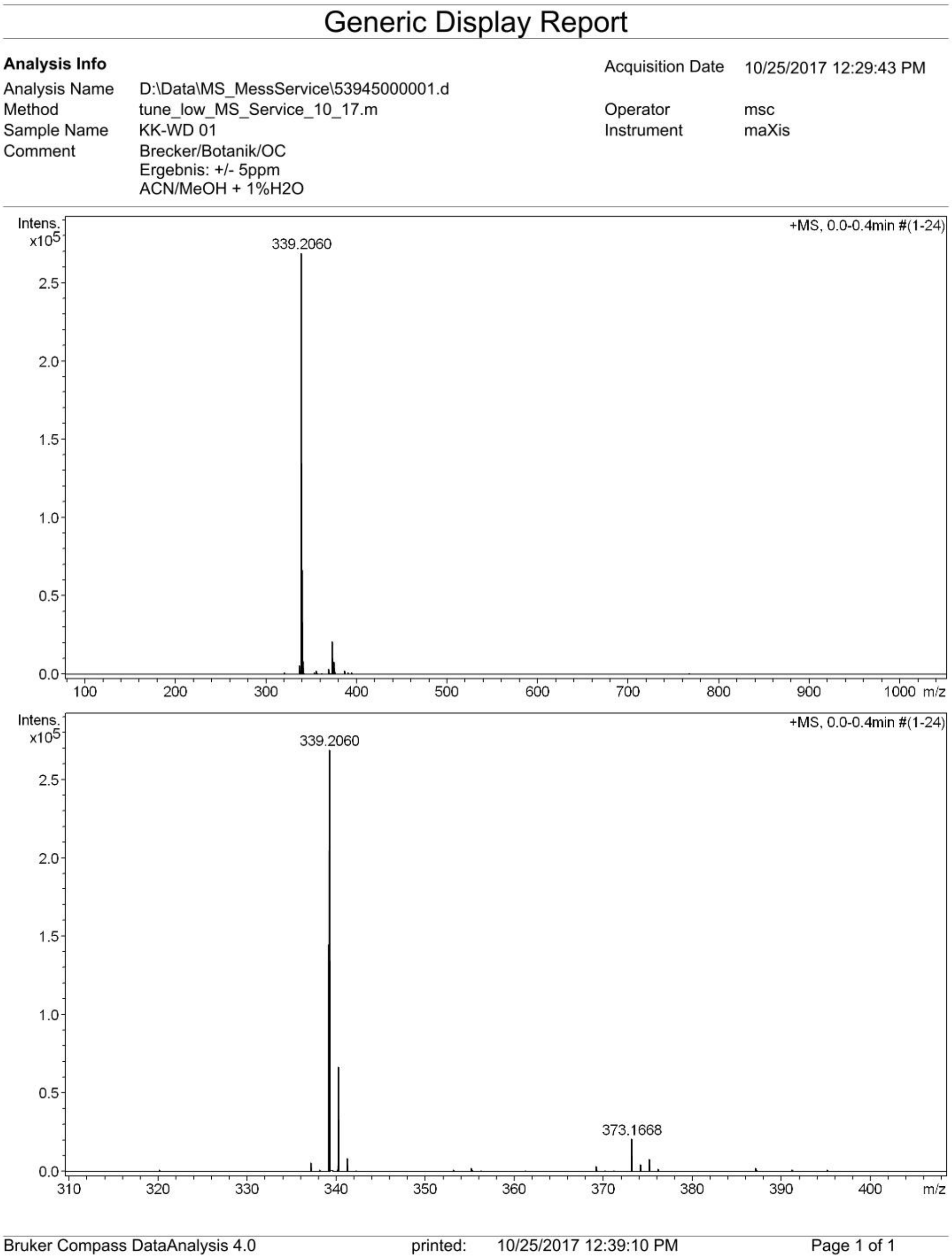


**Figure S10**: pos. mode HR-ESI-MS spectrum of **1**.

# Generic Display Report

**Analysis Info**

| | | | |
|---|---|---|---|
| Analysis Name | E:\Data\MS_MessService\55997000001.d | Acquisition Date | 2/21/2018 9:06:40 AM |
| Method | tune_low_MS_Service_02_18.m | Operator | msc |
| Sample Name | HZ-WDo4 | Instrument | maXis |
| Auftraggeber/Com | Brecker/Botanik<br>Ergebnis: +/- 5ppm<br>ACN/MeOH + 1% H2O | | |

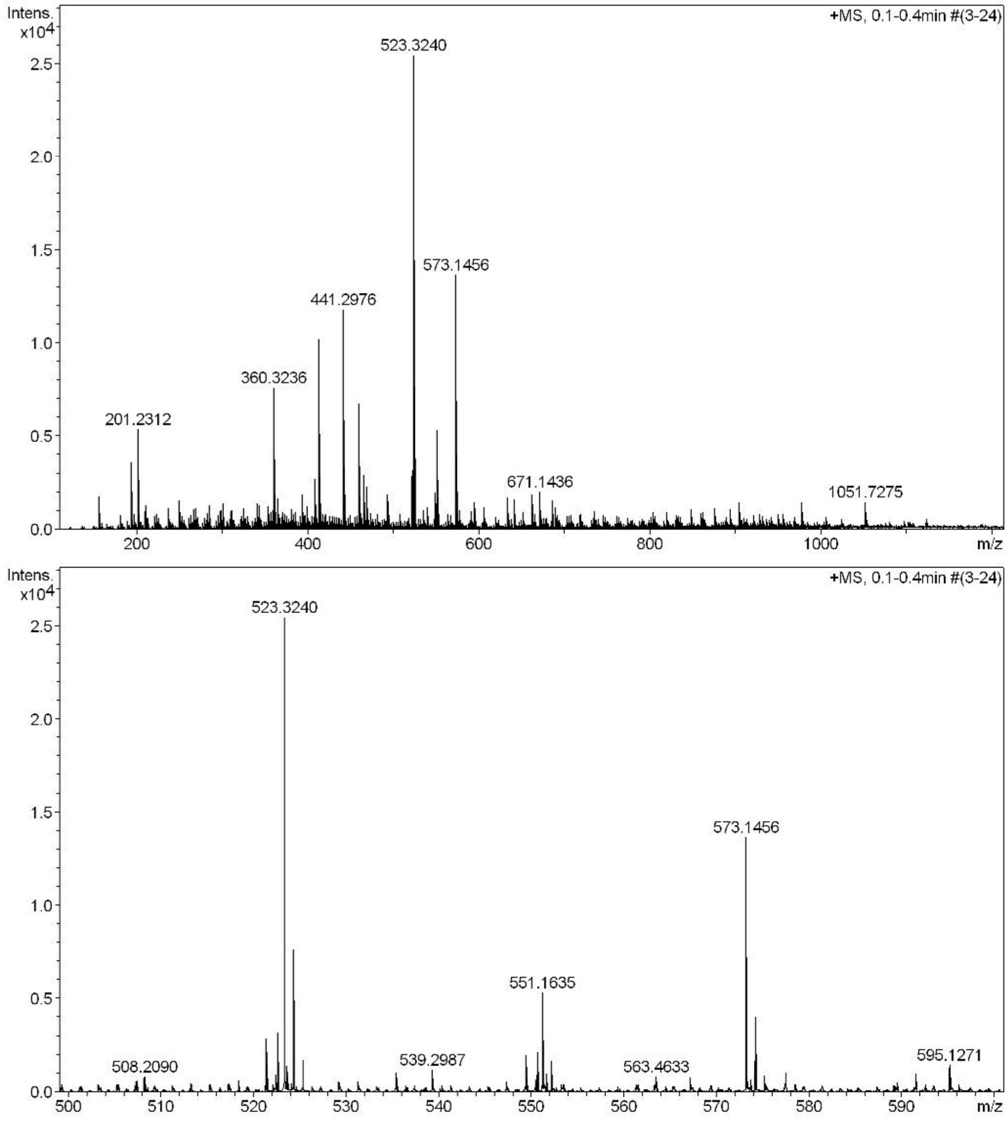


Bruker Compass DataAnalysis 4.0 printed: 2/21/2018 9:15:30 AM Page 1 of 1

**Figure S11***:* pos. mode HR-ESI-MS spectrum of **6**.

## Generic Display Report

**Analysis Info**

| | | | |
|---|---|---|---|
| Analysis Name | E:\Data\MS_MessService\55997000001.d | Acquisition Date | 2/21/2018 9:06:40 AM |
| Method | tune_low_MS_Service_02_18.m | Operator | msc |
| Sample Name | HZ-WDo4 | Instrument | maXis |
| Auftraggeber/Com | Brecker/Botanik<br>Ergebnis: +/- 5ppm<br>ACN/MeOH + 1% H2O | | |

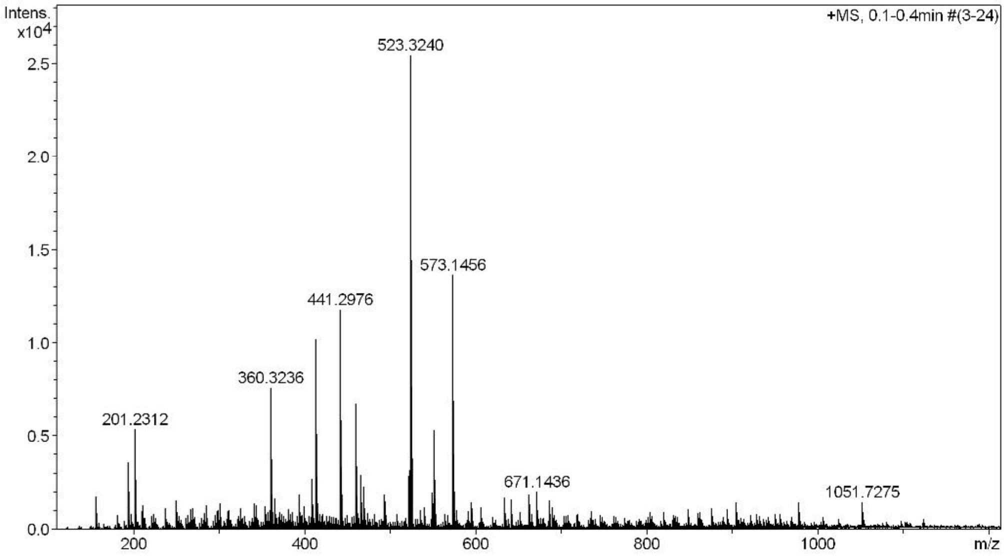


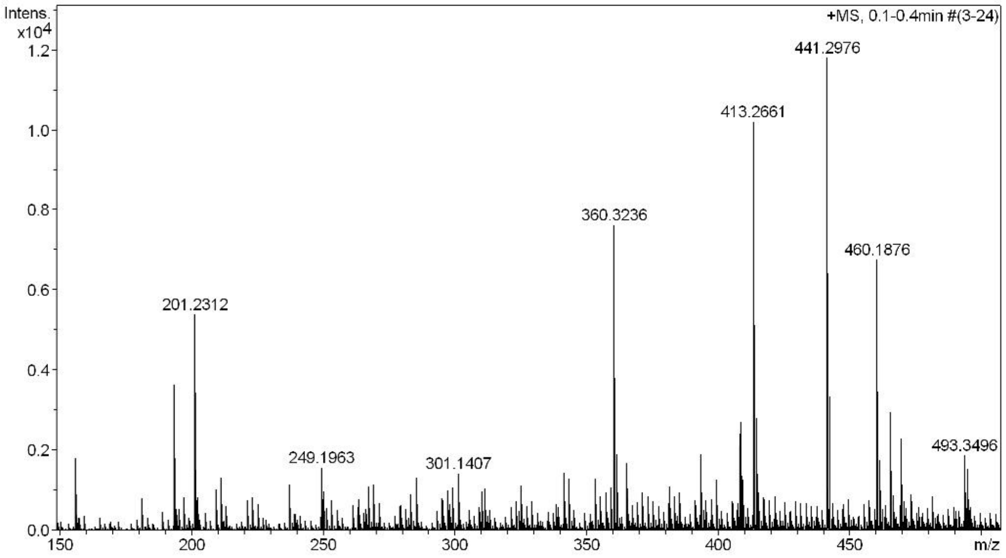


Bruker Compass DataAnalysis 4.0 printed: 2/21/2018 9:14:51 AM Page 1 of 1

**Figure S12***:* pos. mode HR-ESI-MS spectrum of **6** with zoom into the region <500 *m/z*.

# Generic Display Report

**Analysis Info**

| | | | |
|---|---|---|---|
| Analysis Name | E:\Data\MS_MessService\55997000002.d | Acquisition Date | 2/21/2018 9:10:55 AM |
| Method | tune_low_MS_Service_02_18.m | Operator | msc |
| Sample Name | HZ-WDo4 | Instrument | maXis |
| Auftraggeber/Com | Brecker/Botanik<br>Ergebnis: +/- 5ppm<br>ACN/MeOH + 1% H2O | | |

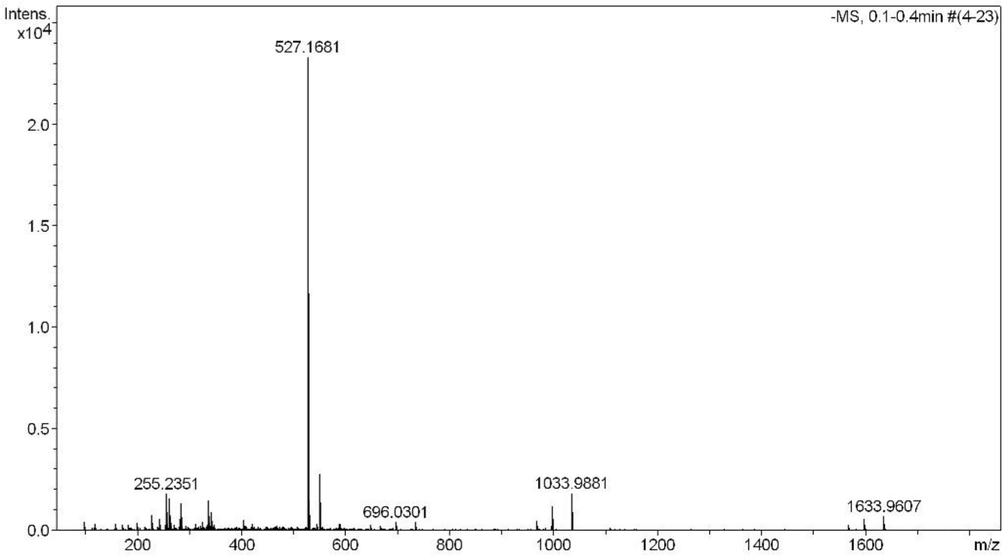


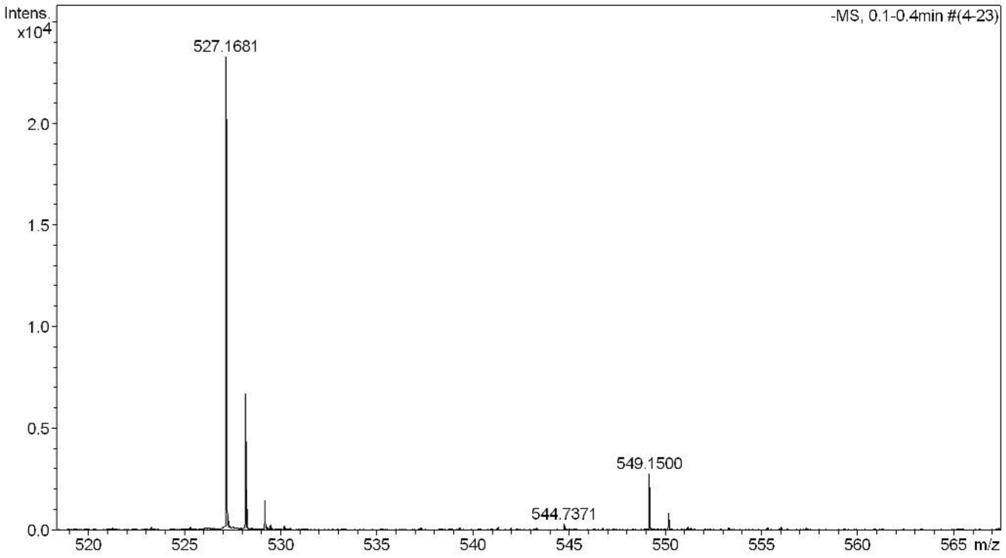




**Figure S13** neg. mode HR-ESI-MS spectrum of **6**.

## 4. NMR Tables

**Table S2**: NMR spectroscopic data of 3-oxocoronaridine (**3**) in $CDCl_3$. Positions of the carbon atoms are indicated in Figure S14. All $^{1}H$ NMR chemical shifts [ppm] are listed together with the relative integral, the multiplicity as well as the coupling constants [Hz]. In addition, the $^{13}C$ NMR chemical shifts and multiplicities are given.

| position | $\delta_C$ [ppm] | $\delta_H$ [ppm] |
|---|---|---|
| 2 | 133.7, s | |
| 3 | 176, s | |
| 5 | 42, 7, t | 4.47 (1H, m) |
| | | 3.25 (1H, dd, *J* = 6.9, 6.9 Hz) |
| 6 | 21.0, t | 3.21 (2H, m) |
| 7 | 109.3, s | |
| 8 | 127.7, s | |
| 9 | 118.3, d | 7.48 (1H, d, *J* = 7.7 Hz) |
| 10 | 119.6, d | 7.09 (1H, dd, *J* = 7.7, 7.7 Hz) |
| 11 | 122.4, d | 7.15 (1H, dd, *J* = 7.4, 7.4 Hz) |
| 12 | 110.6, d | 7.25 (1H, m) |
| 13 | 135.6, s | |
| 14 | 38.0, d | 2.66 (1H, m) |
| 15 | 30.9, t | 2.0 (1H, ddd, *J* = 13.2, 7.7, 3.6 Hz) |
| | | 1.39 (1H, m) |
| 16 | 55.5*, s | |
| 17 | 35.8, t | 2.65 (1H, m) |
| | | 2.32 (1H, m) |
| 18 | 11.4, q | 0.98 (3H, dd, *J* = 7.3, 7.3 Hz) |
| 19 | 27.6, t | 1.52 (1H, m) |
| | | 1.42 (1H, m) |
| 20 | 35.4, d | 1.76 (1H, m) |
| 21 | 56.1, d | 4.52 (1H, s) |
| 22 | 173.0, s | |
| OMe | 53.1, q | 3.73 (3H, s) |

* $^{13}C$ NMR shift indirectly determined through HMBC correlations

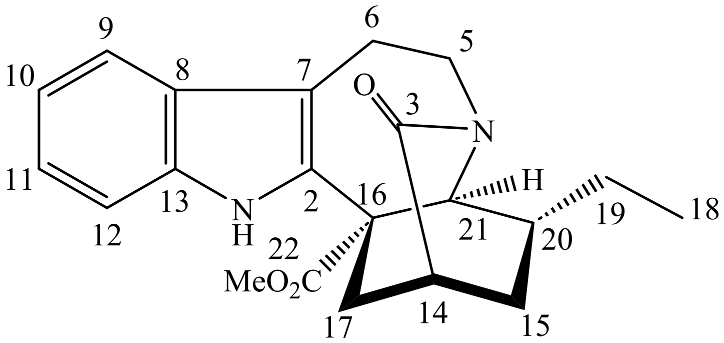


**Figure S14**: 3-oxocoronaridine (**3**).

**Table S3**: NMR spectroscopic data of javaniside (**4**) in $CD_3OD$. Positions of the carbon atoms are indicated in Figure S15. All $^{1}H$ NMR chemical shifts [ppm] are listed together with the relative integral, the multiplicity as well as the coupling constants [Hz]. In addition, the $^{13}C$ NMR chemical shifts and multiplicities are given.

| position | $\delta_C$ [ppm] | $\delta_H$ [ppm] |
|---|---|---|
| 2 | 180.9, s | |
| 3 | 65.5, d | 4.10 (1H, dd, *J* = 11.4, 3.2 H) |
| 5 | 45.6, t | 4.04 (1H, td, *J* = 11.6, 7.8 Hz) |
| | | 3.76 (1H, m) |
| 6 | 33.4, t | 2.41 (1H, ddd, *J* = 13.1, 10.7, 9.6 Hz) |
| | | 2.24 (1H, ddd, *J* = 13.1, 7.8, 1.1 Hz) |
| 7 | 58, s | |
| 8 | 129.5, s | |
| 9 | 123.9, d | 7.32 (1H, dd, *J* = 7.6, 0.6 Hz) |
| 10 | 123.7, d | 7.08 (1H, ddd, *J* = 7.6, 7.6, 1.0 Hz) |
| 11 | 130, d | 7.26 (1H, ddd, *J* = 7.7, 7.6, 1.2 Hz) |
| 12 | 111, d | 6.92 (1H, d, *J* = 7.7 Hz) |
| 13 | 143.5, s | |
| 14 | 26.8, t | 1.38 (1H, ddd, *J* = 12.2, 3.7, 3.7 Hz) |
| | | 1.29 (1H, m) |
| 15 | 28.7, d | 2.99 (1H, m) |
| 16 | 44.7, d | 2.56 (1H, ddd, *J* = 9.8, 5.5, 1.7 Hz) |
| 17 | 97.3, d | 5.43 (1H, d, *J* = 1.9 hz) |
| 19 | 148.2, d | 7.39 (1H, d, *J* = 2.5 Hz) |
| 20 | 108.9, s | |
| 21 | 165.9, s | |
| 22 | 133.9, d | 5.48 (1H, ddd (17.1, 10.1, 9.8 Hz) |
| 23 | 120.5, t | 5.19 (1, H, ddd, *J* = 17.1, 1.8, 0.6 Hz) |
| | | 5.16 (1H, dd, *J* = 10.3, 1.8 Hz) |
| 1' | 99.5, d | 4.65, (1H, d, *J* = 7.9 Hz) |
| 2' | 74.8, d | 3.14 (1H, dd, *J* = 9.3, 7.9 Hz) |
| 3' | 77.9, d | 3.35 (1H, dd, *J* = 8.9, 8.8 Hz) |
| 4' | 71.5, d | 3.25 (1H, dd, *J* = 9.6, 8.7 hz) |
| 5' | 78.3, d | 3.29 (1H, m) |
| 6' | 62.6, t | 3.86 (1H, dd, *J* = 12.0, 2.3 Hz) |
| | | 3.64 (1H, dd, *J* = 12.0, 5.9 Hz) |

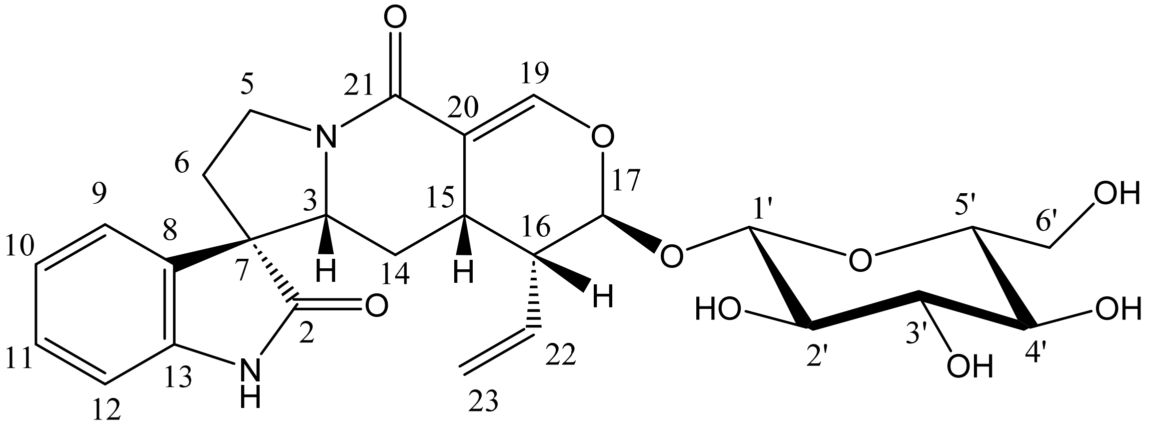

**Figure S15**: javaniside (**4**).

**Table S4**: NMR spectroscopic data of 7-epi javaniside (**5**) in $CD_3OD$. Positions of the carbon atoms are indicated in Figure S16. All $^1H$ NMR chemical shifts [ppm] are listed together with the relative integral, the multiplicity as well as the coupling constants [Hz]. In addition, the $^{13}C$ NMR chemical shifts and multiplicities are given.

| position | $\delta_C$ [ppm] | $\delta_H$ [ppm] |
|---|---|---|
| 2 | 179.1, s | |
| 3 | 64.8, d | 4.08 (1H, dd, *J* = 11.4, 3.3 Hz) |
| 5 | 45.5, t | 3.95 (1H, ddd, *J* = 12.4, 12.1, 7.8 Hz) |
| | | 3.83 (1H, m) |
| 6 | 34.3, t | 2.48 (1H, m) |
| | | 2.03 (1H, ddd, *J* = 12.7, 7.7, 1.1 Hz) |
| 7 | 58.8, s | |
| 8 | 131.2, s | |
| 9 | 124.8, d | 6.9 (1H, d, *J* = 7.3 Hz) |
| 10 | 123.7, d | 7.02 (1H, ddd, *J* = 7.6, 7.3, 0.8 Hz) |
| 11 | 129.8, d | 7.26 (1H, ddd, *J* = 7.8, 7.6, 1.1 Hz) |
| 12 | 111.5, d | 6.97 (1H, d, *J* = 7.8 Hz) |
| 13 | 142.5, s | |
| 14 | 27.2, t | 1.40 (1H, ddd, *J* = 12.7, 3.8, 3.7 Hz) |
| | | 0.89 (1H, m) |
| 15 | 28.2, d | 3.07 (1H, m) |
| 16 | 44.5, d | 2.54 (1H, ddd, *J* = 9.6, 5.5, 1.5 Hz) |
| 17 | 97.3, d | 5.42 (1H, d, *J* = 1.6 Hz) |
| 19 | 148.5, d | 7.41 (1H, d, *J* = 2.4 Hz) |
| 20 | 108.7, s | |
| 21 | 166.1, s | |
| 22 | 133.5, d | 5.3 (1H, ddd, *J* = 17.2, 10.2, 9.6 Hz) |
| 23 | 120.4, t | 5.07 (1H, dd, *J* = 17.2, 1.5 Hz) |
| | | 4.98 (1H, dd, *J* = 10.2, 1.5 Hz) |
| 1’ | 99.5, d | 4.64 (1H, d, *J* = 7.9 Hz) |
| 2’ | 74.7, d | 3.17 (1H, dd, *J* = 9.3, 8.0 Hz) |
| 3’ | 77.9, d | 3.35 (1H, m) |
| 4’ | 71.5, d | 3.27 (1H, m) |
| 5’ | 78.3, d | 3.29 (1H, m) |
| 6’ | 62.7, t | 3.87 (1H, dd, *J* = 12.0, 2.2 Hz) |
| | | 3.64 (1H, dd, *J* = 12.0, 6.0 Hz) |

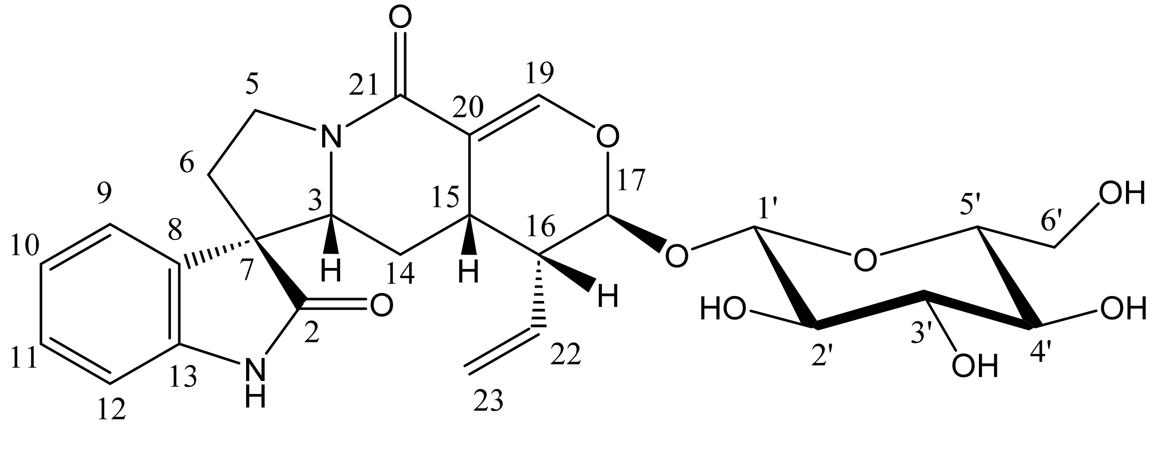


**Figure S16**: 7-epi javaniside (**5**).

**Table S5**: NMR spectroscopic data of voacristine (**7**) in $CD_3OD$. Positions of the carbon atoms are indicated in Figure S17. All $^{1}H$ NMR chemical shifts [ppm] are listed together with the relative integral, the multiplicity as well as the coupling constants [Hz]. In addition, the $^{13}C$ NMR chemical shifts and multiplicities are given.

| position | $\delta_C$ [ppm] | $\delta_H$ [ppm] |
|---|---|---|
| 2 | 138.7, s | |
| 3 | 53.7, t | 2.97 (1H, m) |
| | | 2.82 (1H, br d, *J* = 9.1 Hz) |
| 5 | 53.9, t | 3.40 (1H, m) |
| | | 3.03 (1H, m) |
| 6 | 22.4, t | 3.07 (2H, m) |
| 7 | 110.2, s | |
| 8 | 129.8, s | |
| 9 | 101.1, d | 6.91 (1H, d, *J* = 2.4 Hz) |
| 10 | 155.1, s | |
| 11 | 112.5, d | 6.71 (1H, dd, *J* = 8.7, 2.4 Hz) |
| 12 | 112.4, d | 7.14 (1H, d, *J* = 8.7 Hz) |
| 13 | 132.9, s | |
| 14 | 28.3, d | 1.98 (1H, m) |
| 15 | 25.3, t | 1.78 (1H, m) |
| | | 1.63 (1H, br t, *J* = 11.5 Hz) |
| 16 | 55.5, s | |
| 17 | 37.4, t | 2.74 (1H, m) |
| | | 1.97 (1H, m) |
| 18 | 20.6, q | 1.11 (3H, d, *J* = 6.4 Hz) |
| 19 | 72.7, d | 4.05 (1H, m) |
| 20 | 41.3, d | 1.50 (1H, m) |
| 21 | 59.7, d | 3.83, (1H, m) |
| 22 | 175.5, s | |
| $CO_2Me$ | 53.1, q | 3.71 (3H, s) |
| OMe | 56.3, q | 3.80 (3H, s) |

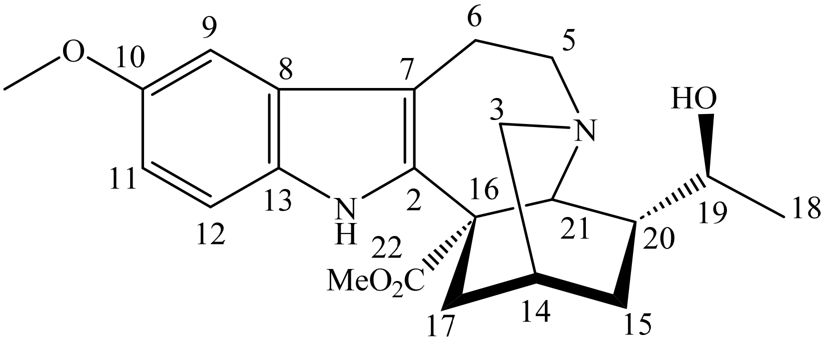


**Figure S17**: voacristine (**7**).

**Table S6**: NMR spectroscopic data of mehranine (**8**) in $CD_3OD$. Positions of the carbon atoms are indicated in Figure S18. All $^1$H NMR chemical shifts [ppm] are listed together with the relative integral, the multiplicity as well as the coupling constants [Hz]. In addition, the $^{13}$C NMR chemical shifts and multiplicities are given.

| position | $\delta_C$ [ppm] | $\delta_H$ [ppm] |
|---|---|---|
| 2 | 74.7, d | 3.30 (1H, m) |
| 3 | 54.0, t | 3.49 (1H, dd, *J* = 13.1, 1.8 Hz) |
| | | 2.44 (1H, d, *J* = 13.1 Hz) |
| 5 | 54.6, t | 3.08 (1H, m) |
| | | 2.25 (1H, m) |
| 6 | 42.1, t | 2.25 (1H, m) |
| | | 1.59 (1H, m) |
| 7 | 52.4*, s | |
| 8 | 138.0, s | |
| 9 | 122.4, d | 7.06 (1H, d, *J* = 7.4 Hz) |
| 10 | 118.5, d | 6.65 (1H, ddd, *J* = 7.4, 7.4, 0.8 Hz) |
| 11 | 128.9, d | 7.04 (1H, ddd, *J* = 7.6, 76, 1.3 Hz) |
| 12 | 108.0, d | 6.41 (1H, d, *J* = 7.5 Hz) |
| 13 | 151.4, s | |
| 14 | 54.1, d | 3.38 (1H, m) |
| 15 | 58.4, d | 2.98 (1H, d, *J* = 4.0 Hz) |
| 16 | 20.8, t | 1.79 (1H, m) |
| | | 1.11 (1H, m) |
| 17 | 24.7, t | 1.79 (1H, m) |
| | | 1.43 (1H, m) |
| 18 | 7.8, q | 0.84 (3H, t, *J* = 7.5 Hz) |
| 19 | 28.9, t | 1.29 (2H, q, *J* = 7.5 Hz) |
| 20 | 36.0, s | |
| 21 | 68.4, d | 2.38 (1H, s) |
| NMe | 31.8, s | 2.74 (3H, s) |

* $^{13}$C NMR shift indirectly determined through HMBC correlations

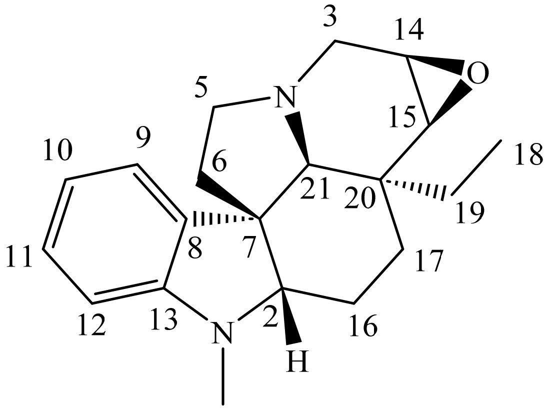


**Figure S18**: mehranine (**8**).

**Table S7**: NMR spectroscopic data of voafinidine epoxide (**9**) in $CD_3OD$. Positions of the carbon atoms are indicated in Figure S19. All $^1H$ NMR chemical shifts [ppm] are listed together with the relative integral, the multiplicity as well as the coupling constants [Hz]. In addition, the $^{13}C$ NMR chemical shifts and multiplicities are given.

| position | $\delta_C$ [ppm] | $\delta_H$ [ppm] |
|---|---|---|
| 2 | 141.6, s | |
| 3 | 54.9, t | 3.22 (1H, d, *J* = 12.6 Hz) |
| | | 2.64 (1H, d, *J* = 12.6 Hz) |
| 5 | 54.6, t | 2.59 (1H, ddd, *J* = 13.2, 4.1, 4.1 Hz) |
| | | 2.30 (1H, ddd, *J* = 13.1, 9.8, 3.0 Hz) |
| 6 | 27.1, t | 2.84 (1H, m) |
| | | 2.81 (1H, m) |
| 7 | 110.1, s | |
| 8 | 129, s | |
| 9 | 118.3, d | 7.37 (1H, d, *J* = 7.6 Hz) |
| 10 | 119.5, d | 6.96 (1H, ddd, *J* = 8.0, 7.7, 1.0 Hz) |
| 11 | 121.3, d | 7.05 (1H, ddd, *J* = 7.6, 7.6, 1.0 Hz) |
| 12 | 109.4, d | 7.24 (1H, d, *J* = 8.2 Hz) |
| 13 | 138.5, s | |
| 14 | 53.8, d | 3.16 (1H, d, *J* = 4.0 Hz) |
| 15 | 60.9, d | 2.92 (1H, dd, *J* = 4.0, 1.3 Hz) |
| 16 | 21.7, t | 4.13 (1H, dd, *J* = 12.4, 12.4 Hz) |
| | | 2.77 (1H, dd, *J* = 14.6, 6.9 Hz) |
| 17 | 36, t | 2.16 (1H, dd, *J* = 14.6, 7.9 Hz) |
| | | 1.79 (1H, ddd, *J* = 14.6, 11.6, 1.2 Hz) |
| 18 | 7.7, q | 0.76 (3H, dd, *J* = 7.5, 7.5 Hz) |
| 19 | 33.4, t | 1.18 (2H, m) |
| 20 | 35.1, s | |
| 21 | 58.8, t | 2.48 (1H, d, *J* = 11.4 Hz) |
| | | 1.70 (1H, d, *J* = 12.0 Hz) |
| NMe | 30.2, q | 3.70 (3H, s) |

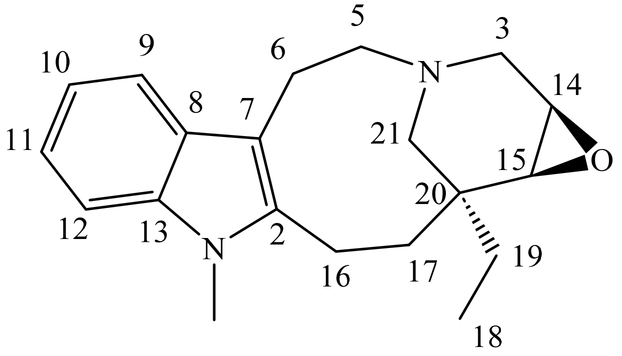


**Figure S19**: voafinidine epoxide (**9**).

**Table S8**: NMR spectroscopic data of voaphylline (**10**) in $CD_3OD$. Positions of the carbon atoms are indicated in Figure S20. All $^1H$ NMR chemical shifts [ppm] are listed together with the relative integral, the multiplicity as well as the coupling constants [Hz]. In addition, the $^{13}C$ NMR chemical shifts and multiplicities are given.

| position | $\delta_C$ [ppm] | $\delta_H$ [ppm] |
|---|---|---|
| 2 | 140.7, s | |
| 3 | 55.0, t | 3.25 (1H, d, *J* = 12.5 Hz) |
| | | 2.63 (1H, d, *J* =12.5 Hz) |
| 5 | 54.8, t | 2.59 (1H, ddd, *J* = 13.2, 3.8, 3.8 Hz) |
| | | 2.24 (1H, ddd, *J* = 13.2, 9.1, 4.3 Hz) |
| 6 | 27.0, t | 2.80 (2H, m) |
| 7 | 109.6, s | |
| 8 | 129.6, s | |
| 9 | 118.1, d | 7.34 (1H, d, *J* = 7.7 Hz) |
| 10 | 119.1, d | 6.91 (1H, ddd, *J* = 8.0, 6.8, 1.2 Hz) |
| 11 | 121.0, s | 6.97 (1H, ddd, *J* = 8.1, 6.9, 1.1 Hz) |
| 12 | 111.1, d | 7.23 (1H, d, *J* = 7.9 Hz) |
| 13 | 137.4, s | |
| 14 | 53.7, d | 3.15 (1H, d, *J* = 4.1 Hz) |
| 15 | 61.0, d | 2.91 (1H, dd, *J* = 4.1, 1.4 Hz) |
| 16 | 24.0, t | 4.07 (1H, dd, *J* = 13.4, 12.9 Hz) |
| | | 2.69 (1H, ddd, *J* = 13.8, 7.3, 4.1 Hz) |
| 17 | 37.1, t | 2.14 (1H, dddd, *J* = 14.5, 7.2, 1.3, 1.3 Hz) |
| | | 1.91 (1H, ddd, *J* = 14.5, 12.2, 1.5 Hz) |
| 18 | 7.6, q | 0.74 (1H, dd, *J* = 7.5, 7.5 Hz) |
| 19 | 33.4, t | 1.15 (2H, m) |
| 20 | 34.8, s | |
| 21 | 59.4, t | 2.32 (1H, d, *J* = 12.0 Hz) |
| | | 1.66 (1H, d, *J* = 12.0 Hz) |

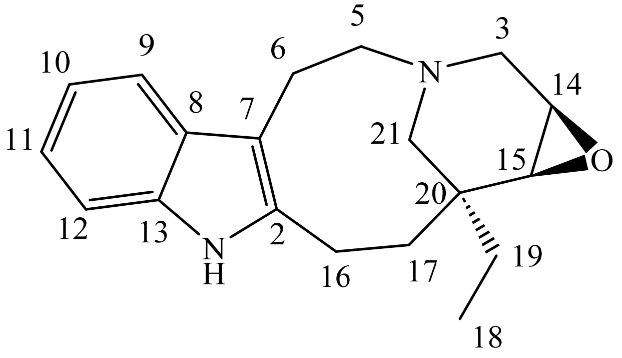


**Figure S20**: voaphylline (**10**).

**Table S9**: NMR spectroscopic data of apparicine (**11**) in $CD_3OD$. Positions of the carbon atoms are indicated in Figure S21. All $^1H$ NMR chemical shifts [ppm] are listed together with the relative integral, the multiplicity as well as the coupling constants [Hz]. In addition, the $^{13}C$ NMR chemical shifts and multiplicities are given.

| position | $\delta_C$ [ppm] | $\delta_H$ [ppm] |
|---|---|---|
| 2 | 133.5, s | - |
| 3 | 46.1, t | 3.37 (1H, m) |
| | | 3.12 (1H, m) |
| 6 | 54.3, t | 4.52 (1H, d, *J* = 17.0 Hz) |
| | | 4.25 (1H, d, *J* = 17.0 Hz) |
| 7 | 108.1, s | - |
| 8 | 129.9, s | - |
| 9 | 118.8, d | 7.35 (1H, d, *J* = 8.1 Hz) |
| 10 | 120, d | 6.97 (1H, m) |
| 11 | 123.6, d | 7.11 (1H, m) |
| 12 | 111.6, d | 7.29 (1H, d, *J* = 8.1 Hz) |
| 13 | 137.6, s | - |
| 14 | 29.3, t | 2.27 (1H, m) |
| | | 1.93 (1H, m) |
| 15 | 42.3, d | 4.01 (1H, m) |
| 16 | 145.1, S | - |
| 17 | 114.2, t | 5.62 (1H, s) |
| | | 5.34 (1H, s) |
| 18 | 13, q | 1.53 (3H, dd, *J* = 6.8, 2.1 Hz) |
| 19 | 123.6, d | 5.42 (1H, q, *J* = 6.7 Hz) |
| 20 | 136.3, s | - |
| 21 | 55.1, t | 3.87 (1H, d, *J* = 15.3 Hz) |
| | | 3.33 (1H, m) |

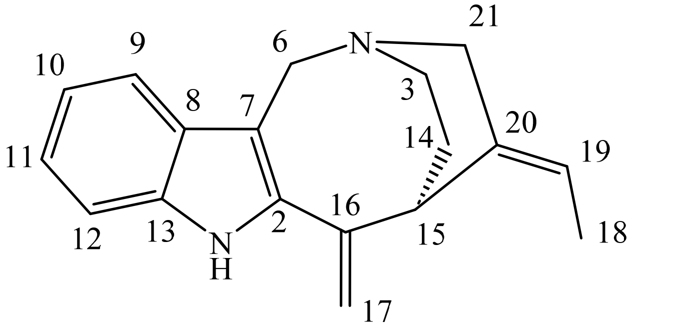


**Figure S21**: apparicine (**11**).

**Table S10**: NMR spectroscopic data of tabernaemontanine (**12**) in $CD_3OD$. Positions of the carbon atoms are indicated in Figure S22. All $^1H$ NMR chemical shifts [ppm] are listed together with the relative integral, the multiplicity as well as the coupling constants [Hz]. In addition, the $^{13}C$ NMR chemical shifts and multiplicities are given.

| position | $\delta_C$ [ppm] | $\delta_H$ [ppm] |
|---|---|---|
| 2 | 135.2, s | |
| 3 | 192.9, S | |
| 5 | 58.2, d | 3.89 (1H, m) |
| 6 | 19.6, t | 3.51 (1H, m) |
| | | 3.38 (1H, dd, *J* = 14.8, 8.0 Hz) |
| 7 | 122.3, s | |
| 8 | 129.7, s | |
| 9 | 121.9, d | 7.73 (1H, d, *J* = 8.1 Hz) |
| 10 | 121, d | 7.10 (1H, m) |
| 11 | 127.5, d | 7.29 (1H, m) |
| 12 | 113.1, d | 7.38 (1H, d, *J* = 8.1 Hz) |
| 13 | 138.8, s | |
| 14 | 46.8, t | 3.52 (1H, m) |
| | | 2.63 (1H, m) |
| 15 | 33.1, s | 2.62 (1H, m) |
| 16 | 44.6, s | 2.93 (1H, dd, *J* = 3.2, 3.2 Hz) |
| 18 | 12.9, q | 0.99 (3H, dd, *J* = 7.3, 7.0 Hz) |
| 19 | 26.4, t | 1.72 (1H, m) |
| | | 1.53 (1H, m) |
| 20 | 43.9, d | 1.54 (1H, m) |
| 21 | 47.6, t | 3.26 (1H, dd, *J* = 13.0, 3.9 Hz) |
| | | 2.53 (1H, d, *J* = 13.2 Hz) |
| 22 | 173.4, s | |
| NMe | 43.2, q | 2.56 (3H, s) |
| OMe | 50.9, q | 2.64 (3H, br s) |

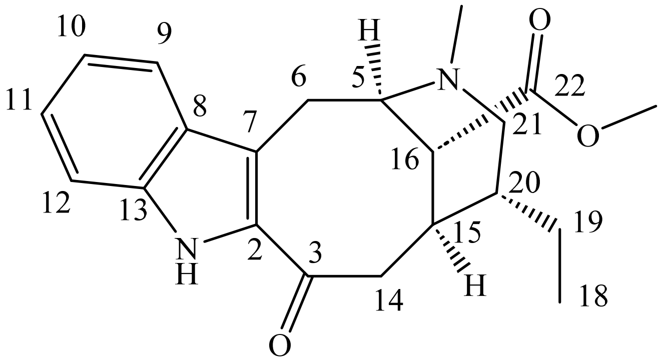


**Figure 22**: tabernaemontanine (**12**).

**Table S11**: NMR spectroscopic data of dregamine (**13**) in $CD_3OD$. Positions of the carbon atoms are indicated in Figure S23. All $^1H$ NMR chemical shifts [ppm] are listed together with the relative integral, the multiplicity as well as the coupling constants [Hz]. In addition, the $^{13}C$ NMR chemical shifts and multiplicities are given.

| position | $\delta_C$ [ppm] | $\delta_H$ [ppm] |
|---|---|---|
| 2 | 135.4, s | |
| 3 | 193.7, s | |
| 5 | 58.0, d | 3.92 (1H, ddd, *J* = 7.9, 7.9, 3.3 Hz) |
| 6 | 21.3, t | 3.44 (1H, m) |
| | | 3.38 (1H, m) |
| 7 | 121.8*, s | |
| 8 | 129.6, s | |
| 9 | 121.9, d | 7.74 (1H, d, *J* = 8.2 Hz) |
| 10 | 121.1, d | 7.11 (1H, dd, *J* = 7.6, 7.6 Hz) |
| 11 | 127.5, d | 7.30 (1H, ddd, *J* = 6.8, 6.8, 0.8 Hz) |
| 12 | 113.2, d | 7.38 (1H, d, *J* = 8.3 Hz) |
| 13 | 138.8, s | |
| 14 | 40.2, t | 3.22 (1H, m) |
| | | 2.54 (1H, m) |
| 15 | 31.8, d | 2.81 (1H, m) |
| 16 | 49.9, d | 2.80 (1H, m) |
| 18 | 11.6, q | 1.04 (3H, dd, *J* = 7.4, 7.4 Hz) |
| 19 | 24.4, t | 1.38 (2H, m) |
| 20 | 44.3, d | 1.88 (1H, m) |
| 21 | 49.7, t | 2.85 (1H, m) |
| | | 2.57 (1H, m) |
| 22 | 172.7, s | |
| NMe | 42.6, q | 2.61 (3H, s) |
| OMe | 51.0, q | 2.66 (1H, s) |

* $^{13}C$ NMR shift indirectly determined through HMBC correlations

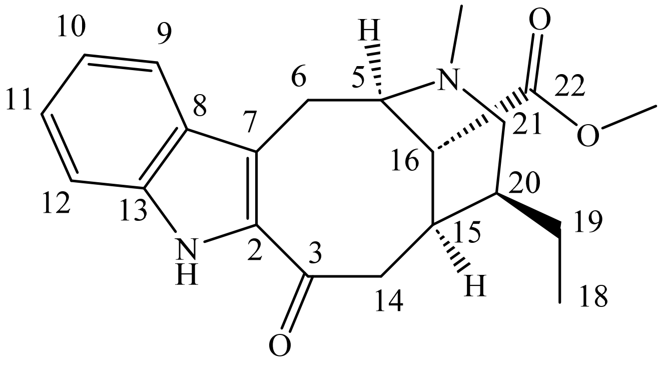


**Figure S23**: dregamine (**13**).

**Table S12**: NMR spectroscopic data of 3-hydroxy coronaridine (**14**) in $CD_3OD$. Positions of the carbon atoms are indicated in Figure S24. All $^{1}H$ NMR chemical shifts [ppm] are listed together with the relative integral, the multiplicity as well as the coupling constants [Hz]. In addition, the $^{13}C$ NMR chemical shifts and multiplicities are given.

| position | $\delta_C$ [ppm] | $\delta_H$ [ppm] |
|---|---|---|
| 2 | 138.4, s | |
| 3 | 98.5, d | 4.00 (1H, d, *J* = 2.1 Hz) |
| 5 | 54.7, t | 3.56 (1H, m) |
| | | 3.04 (1H, m) |
| 6 | 22.7, t | 3.05 (2H, m) |
| 7 | 110.4, s | |
| 8 | 129.2, s | |
| 9 | 118.7, d | 7.42 (1H, d, *J* = 7.9 Hz) |
| 10 | 119.6, d | 6.97 (1H, ddd, *J* = 7.9, 7.6, 1.0 Hz) |
| 11 | 122.2, d | 7.03 (1H, ddd, *J* = 7.9, 7.6, 1.1 Hz) |
| 12 | 111.6, d | 7.23 (1H, d, *J* = 8.0 Hz) |
| 13 | 137.7, s | |
| 14 | 31.3, d | 2.05 (1H, m) |
| 15 | 26.1, t | 1.51 (1H, m) |
| | | 1.43 (1H, m) |
| 16 | 55.7*, s | |
| 17 | 35.8, t | 2.92 (1H, dd, *J* = 14.1, 2.5 Hz) |
| | | 1.89 (1H, ddd, *J* = 14.1, 2.7, 2.5 Hz) |
| 18 | 12.1, q | 0.91 (3 H, m) |
| 19 | 27.7, t | 1.63, (1H, m) |
| | | 1.50 (1H, m) |
| 20 | 38.8, d | 1.37 (1H, m) |
| 21 | 56.4, d | 3.86 (1H, d, *J* = 0.8 Hz) |
| 22 | 175.7, s | |
| OMe | 52.9, q | 3.67 (3H, s) |

* $^{13}C$ NMR sift indirectly determined through HMBC correlations

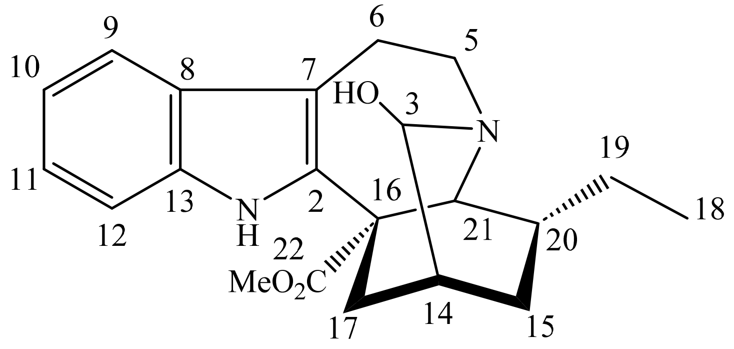


**Figure S24**: 3-hydroxy coronaridine (**14**).

**Table S13**: NMR spectroscopic data of ervatamine (**15**) in $CD_3OD$. Positions of the carbon atoms are indicated in Figure S25. All $^1H$ NMR chemical shifts [ppm] are listed together with the relative integral, the multiplicity as well as the coupling constants [Hz]. In addition, the $^{13}C$ NMR chemical shifts and multiplicities are given.

| position | $\delta_C$ [ppm] | $\delta_H$ [ppm] |
|---|---|---|
| 2 | 134.2, s | |
| 3 | 196.2, s | |
| 5 | 61, t | 3.51 (1H, d, *J* = 11.6 Hz) |
| | | 2.29 (1H, d, *J* = 12.3 Hz) |
| 6 | 32.6, t | 3.51 (1H, d, *J* = 15.7 Hz) |
| | | 2.94 (1H, d, *J* = 15.7 Hz) |
| 7 | 120.6, s | |
| 8 | 128.4, s | |
| 9 | 120.9, d | 7.56 (1H, d, *J* = 8.2 Hz) |
| 10 | 121.3, d | 7.09 (1H, dd, *J* = 7.5, 7.5 Hz) |
| 11 | 127.3, d | 7.29 (1H, dd, *J* = 8.4, 6.9 Hz) |
| 12 | 113.6, d | 7.42 (1H, d, *J* = 8.4 Hz) |
| 13 | 138.9, s | |
| 14 | 37.3, t | 2.67 (1H, dd, *J* = 15.8, 10.3 Hz) |
| | | 2.52 (1H, m) |
| 15 | 37.4, d | 2.52 (1H, m) |
| 16 | 50.5, s | |
| 18 | 11.6, q | 0.88 (3H, dd, *J* = 7.4, 7.4 Hz) |
| 19 | 24.8, t | 1.39 (1H, m) |
| | | 1.36 (1H, m) |
| 20 | 40, d | 1.79 (1H, m) |
| 21 | 58.3, t | 2.65 (1H, m) |
| | | 1.73 (1H, dd, *J* = 11.4, 11.4 Hz) |
| 22 | 177, s | |
| NMe | 46.3, q | 2.34 (3H, s) |
| OMe | 53, q | 3.64 (3H, s) |

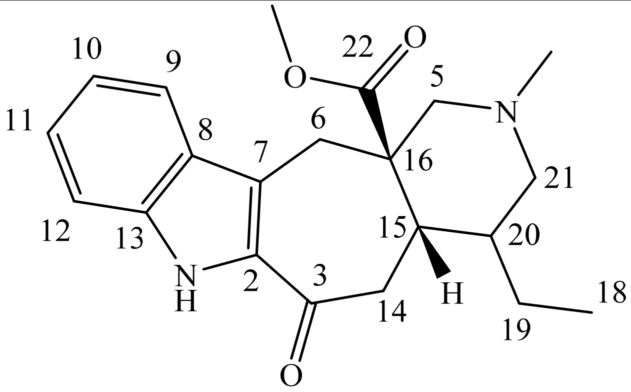


**Figure S25**: ervatamine (**15**).

**Table S14**: NMR spectroscopic data of 19, 20-didehydro ervatamine (**16**) in $CD_3OD$. Positions of the carbon atoms are indicated in Figure S26. All $^1H$ NMR chemical shifts [ppm] are listed together with the relative integral, the multiplicity as well as the coupling constants [Hz]. In addition, the $^{13}C$ NMR chemical shifts and multiplicities are given.

| position | $\delta_C$ [ppm] | $\delta_H$ [ppm] |
|---|---|---|
| 2 | 134.1, s | |
| 3 | 195.5, s | |
| 5 | 61.6, t | 3.55 (1H, d, *J* = 11.1 Hz) |
| | | 2.49 (1H, d, *J* = 12.4 Hz) |
| 6 | 32, t | 3.58 (1H, d, *J* = 15.4 Hz) |
| | | 3.02 (1H, d, *J* = 15.4 Hz) |
| 7 | 120.7, s | |
| 8 | 128.4, s | |
| 9 | 121, d | 7.59 (1H, d, *J* = 8.2 Hz) |
| 10 | 121.4, d | 7.1 (1H, dd, *J* = 8.2, 6.7 Hz) |
| 11 | 127.4, d | 7.31 (1H, dd, *J* = 8.4, 6.7 Hz) |
| 12 | 113.6, d | 7.44 (1H, d, *J* = 8.4 Hz) |
| 13 | 139, s | |
| 14 | 45, t | 3.29 (1H, d, *J* = 11.3 Hz) |
| | | 2.31 (1H, d, *J* = 16.0 Hz) |
| 15 | 35.5, d | 3.52 (1H, s) |
| 16 | 50.3, s | |
| 18 | 12.7, q | 1.57 (3H, dd, *J* = 6.7, 1.8 Hz) |
| 19 | 123.5, d | 5.47 (1H, q, *J* = 6.7 Hz) |
| 20 | 137.2, s | |
| 21 | 61.8, t | 3.15 (1H, d, *J* = 11.9 Hz) |
| | | 2.74 (1H, d, *J* = 11.9 Hz) |
| 22 | 176.6, s | |
| NMe | 45.9, q | 2.35 (3H, s) |
| OMe | 53, q | 3.58 (3H, s) |

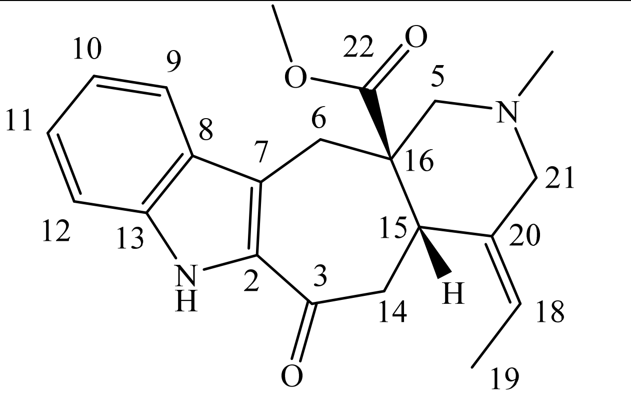


**Figure S26:** 19, 20-didehydro ervatamine (**16**).

**Table S15**: NMR spectroscopic data of secologanoside (**17**) in $CD_3OD$. Positions of the carbon atoms are indicated in Figure S27. All $^1H$ NMR chemical shifts [ppm] are listed together with the relative integral, the multiplicity as well as the coupling constants [Hz]. In addition, the $^{13}C$ NMR chemical shifts and multiplicities are given.

| position | $\delta_C$ [ppm] | $\delta_H$ [ppm] |
|---|---|---|
| 1 | 97.3, d | 5.43 (1 H, d, *J* = 3.6 Hz) |
| 2 | | |
| 3 | 151.3, d | 7.33 (1H, s) |
| 4 | 112.5, s* | |
| 5 | 28.8, d | 3.28 (1H, m) |
| 6 | 35.7, t | 3.04 (1H, m) |
| | | 2.21 (1H, dd, *J* = 16.4, 10.0 Hz) |
| 7 | n.d. | |
| 8 | 135, d | 5.66 (1H, ddd, *J* = 17.1, 10.0, 10.0 Hz) |
| 9 | 45.3, d | 2.83 (1H, m) |
| 10 | 120.2, t | 5.24 (1H, dd, *J* = 17.8, 1.8 Hz) |
| | | 5.21 (1H, dd, *J* = 10.4, 1.8 Hz) |
| 11 | 171.8, s* | |
| 1' | 99.8, d | 4.65 (1H, d, *J* = 8.0 Hz) |
| 2' | 74.9, d | 3.22 (1H, dd, *J* = 9.0, 8.0 Hz) |
| 3' | 77.9, d | 3.36 (1H, dd, *J* = 9.0, 9.0 Hz) |
| 4' | 71.6, d | 3.29 (1H, m) |
| 5' | 78.3, d | 3.30 (1H, m) |
| 6' | 62.7, t | 3.88 (1H, dd, *J* = 12.1, 1.7 Hz) |
| | | 3.67 (1H, dd, *J* = 12.1, 5.5 Hz) |

* $^{13}C$ NMR sift indirectly determined through HMBC correlations

n.d. not determined

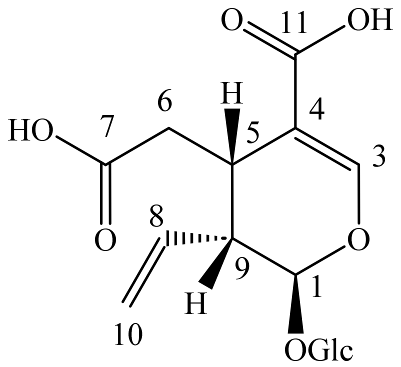


**Figure S27**: secologanoside (**17**).

**Table S16**: NMR spectroscopic data of loganic acid (**18**) in $CD_3OD$. Positions of the carbon atoms are indicated in Figure S28. All $^1H$ NMR chemical shifts [ppm] are listed together with the relative integral, the multiplicity as well as the coupling constants [Hz]. In addition, the $^{13}C$ NMR chemical shifts and multiplicities are given.

| position | $\delta_C$ [ppm] | $\delta_H$ [ppm] |
|---|---|---|
| 1 | 97.6, d | 5.27 (1H, d, *J* = 4.4 Hz) |
| 3 | 151.7, d | 7.38 (1H, d, *J* = 1.1 Hz) |
| 4 | 114.6, s | |
| 5 | 32.2, d | 3.10 (1H, ddd, *J* = 8.0, 8.0, 8.0 Hz) |
| 6 | 42.7, t | 1.66 (1 H, m) |
| | | 2.24 (1 H, m) |
| 7 | 75.1, d | 4.04 (1 H, m) |
| 8 | 42.1, d | 1.88 (1H, m) |
| 9 | 46.6, d | 2.03 (1H, m) |
| 10 | 13.4, q | 1.10 (3H, d, *J* = 7.0 Hz) |
| 11 | 171.3, s | |
| 1' | 100, d | 4.65 (1H, d, *J* = 8.1 Hz) |
| 2' | 74.8, d | 3.20 (1H, dd, *J* = 9.1, 8.1 Hz) |
| 3' | 78.0, d | 3.36 (1H, m) |
| 4' | 71.6, d | 3.28 (1H, m) |
| 5' | 78.4, d | 3.30 (1H, m) |
| 6' | 62.8, t | 3.67 (1H, dd, *J* = 11.8, 5.7 Hz) |
| | | 3.89 (1H, dd, *J* = 12.0, 1.9 Hz) |

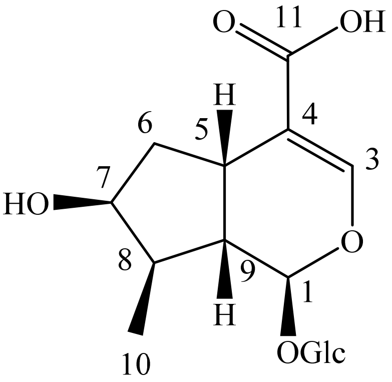


Figure S28: loganic acid (**18**).

## 5. Comparison of 13C NMR spectra of compounds 4, 5 and 6

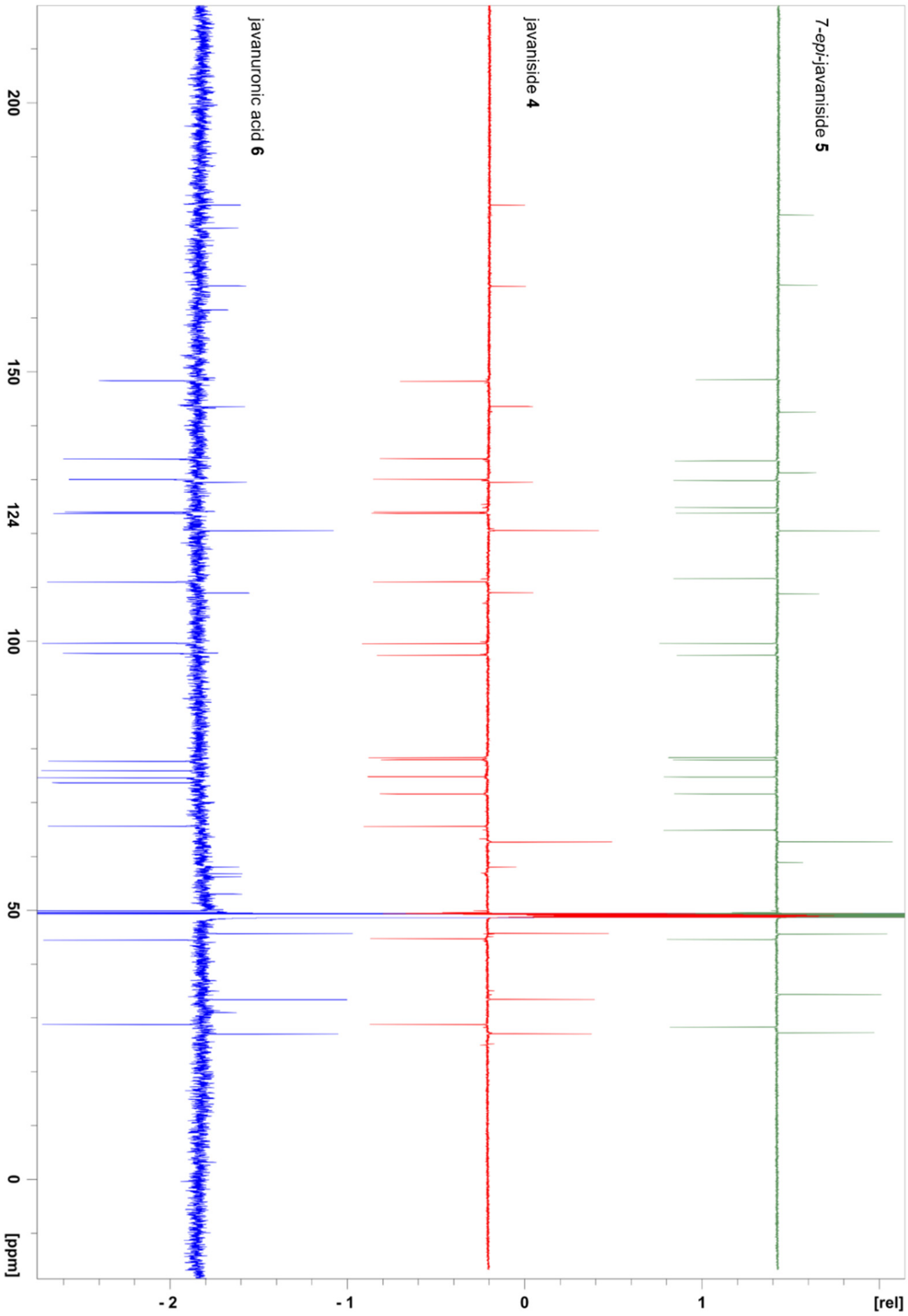


**Figure S29**: Comparison of $^{13}C$ NMR spectra of javanuronic acid (**6**), javaniside (**4**) and 7-epi-javaniside (**5**).

# 6. $^{1}H$ and $^{13}C$ NMR spectra of isolated compounds

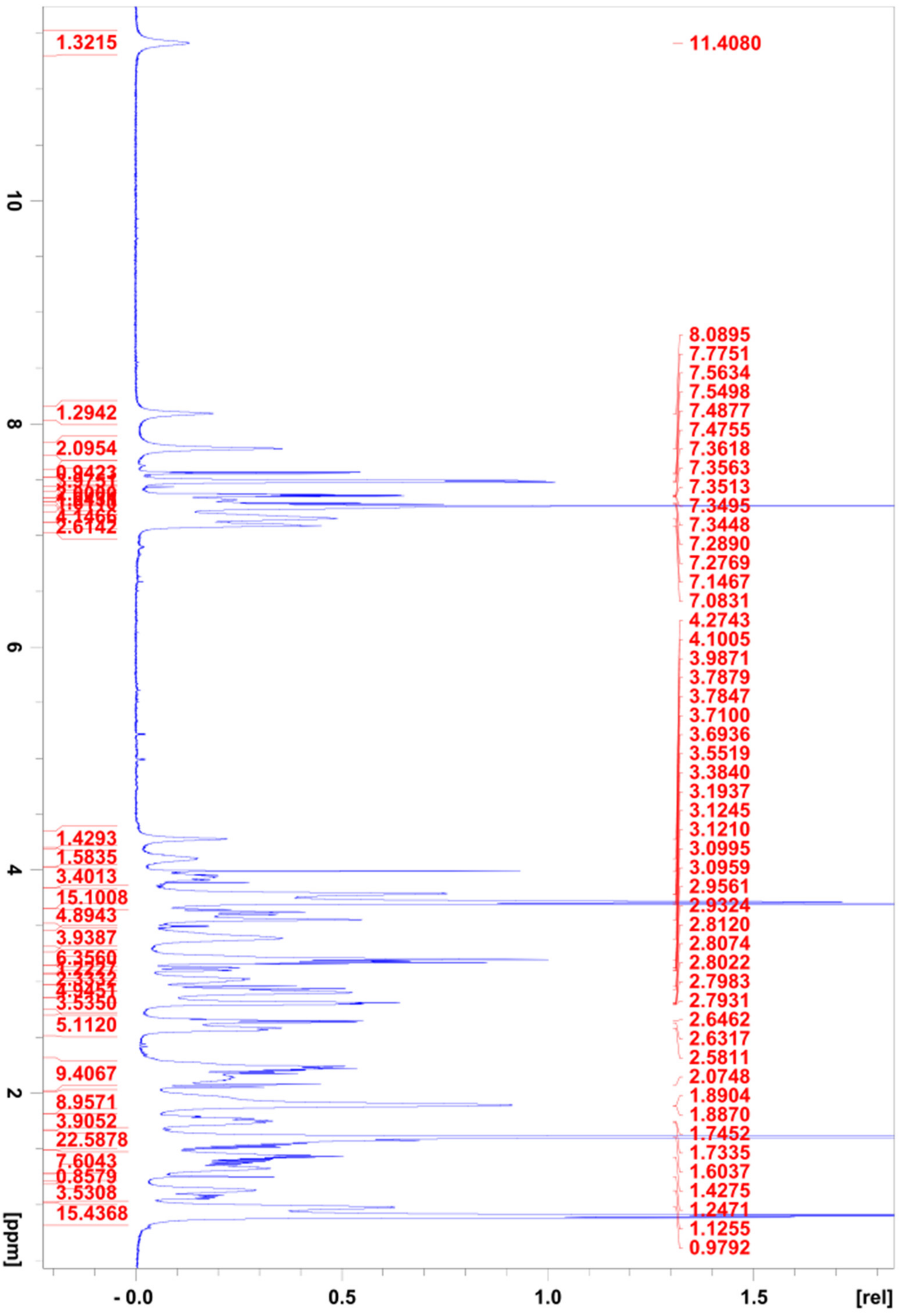


**Figure S30**: $^{1}H$ NMR of 3,7-coronaridine isoindolenine (**1**) in $CDCl_3$.

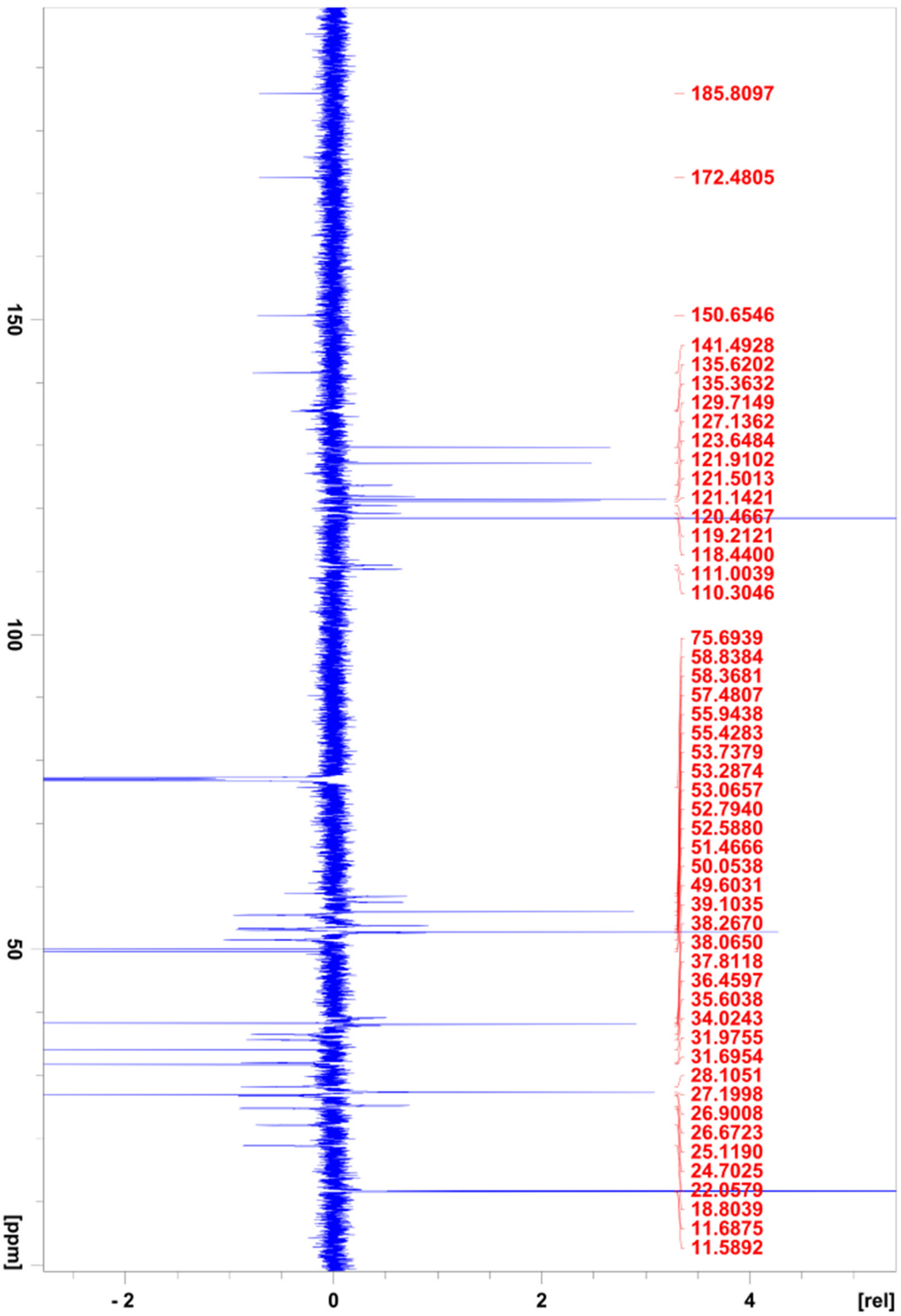


**Figure S31**: $^{13}C$ NMR of 3,7-coronaridine isoindolenine (**1**) in $CDCl_3$.

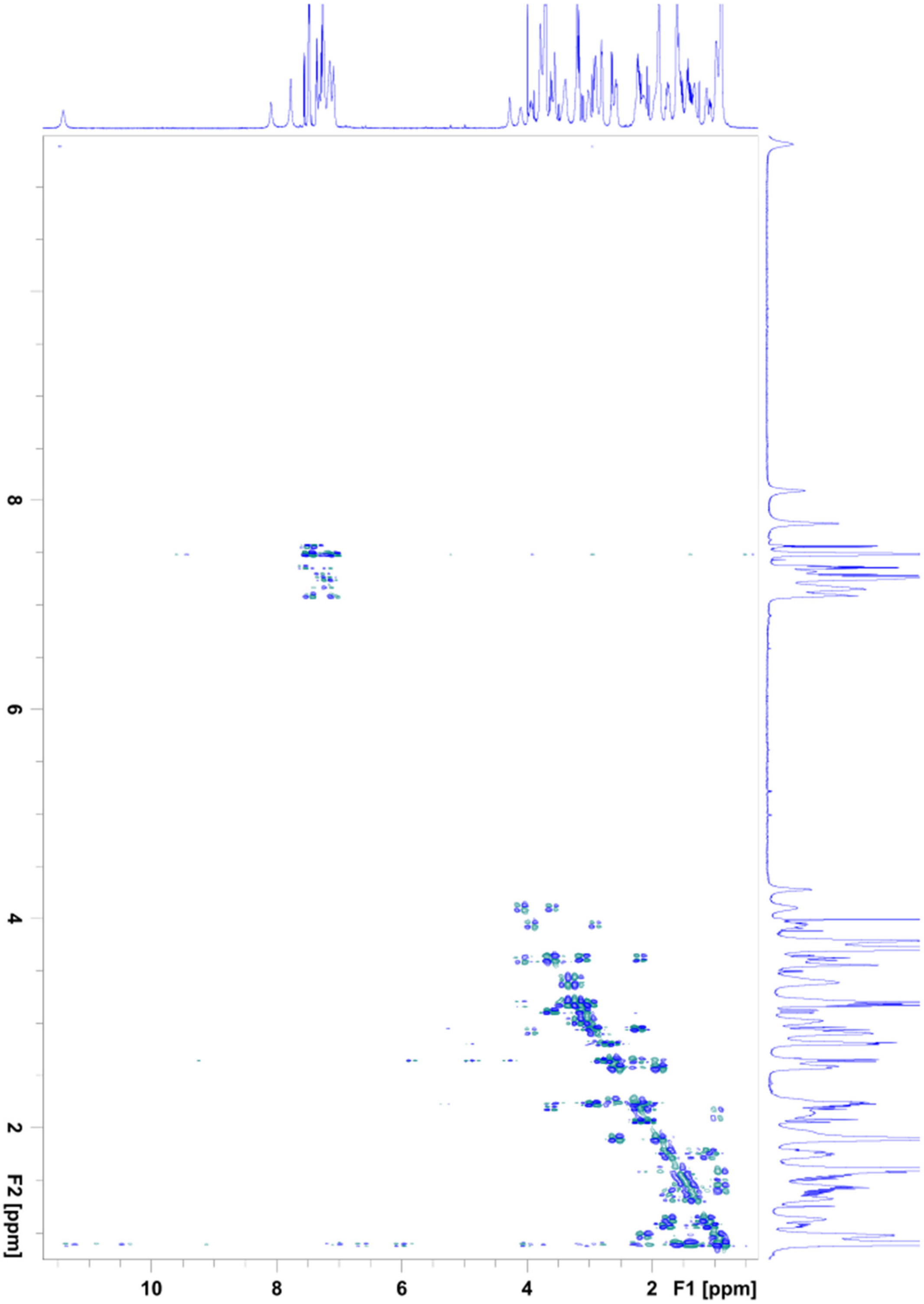


**Figure S32**: COSY spectrum of 3,7-coronaridine isoindolenine (**1**) in $CDCl_3$.

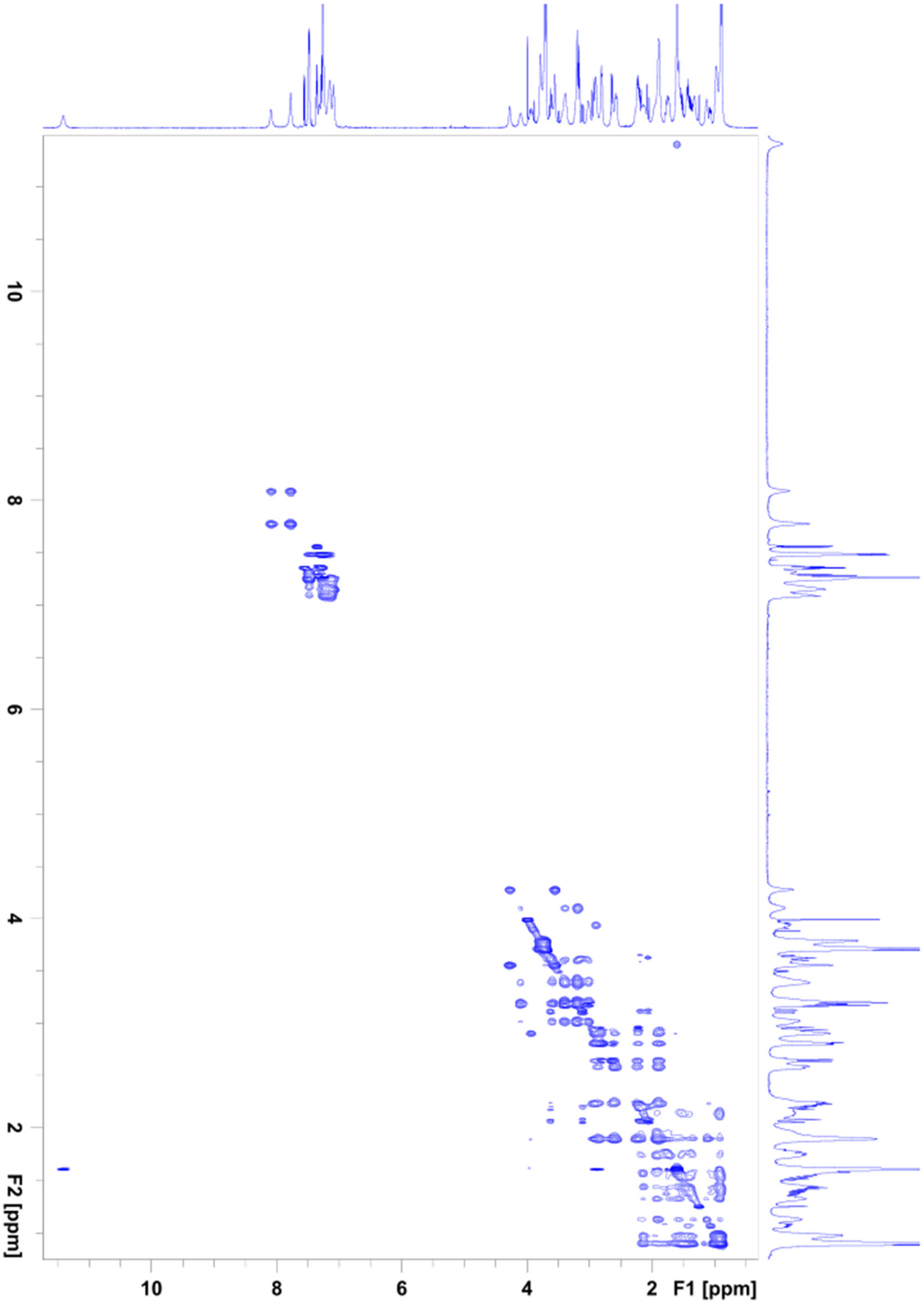


**Figure S33**: TOCSY spectrum of 3,7-coronaridine isoindolenine (**1**) in $CDCl_3$.

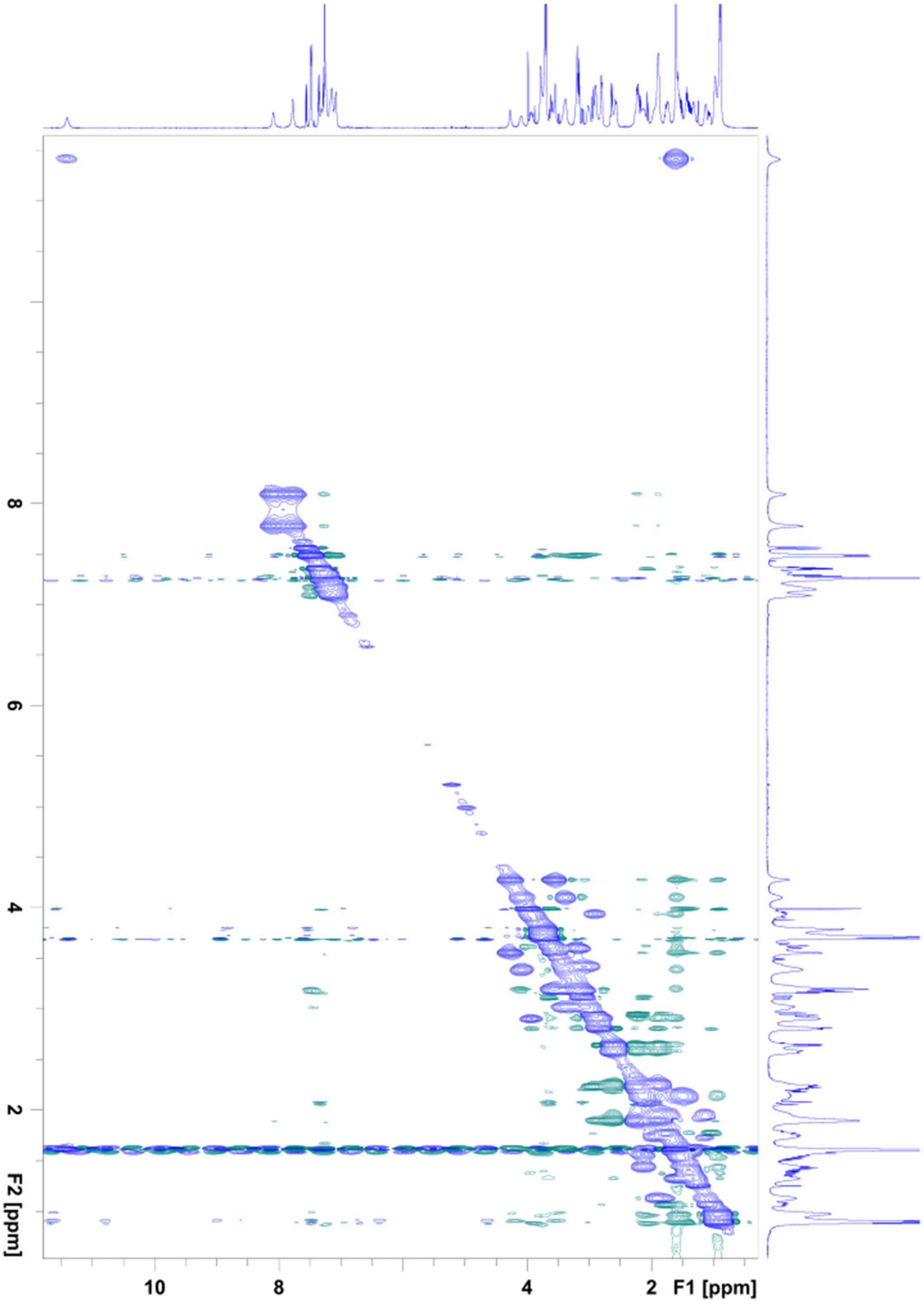


**Figure S34**: NOESY spectrum of 3,7-coronaridine isoindolenine (**1**) in $CDCl_3$.

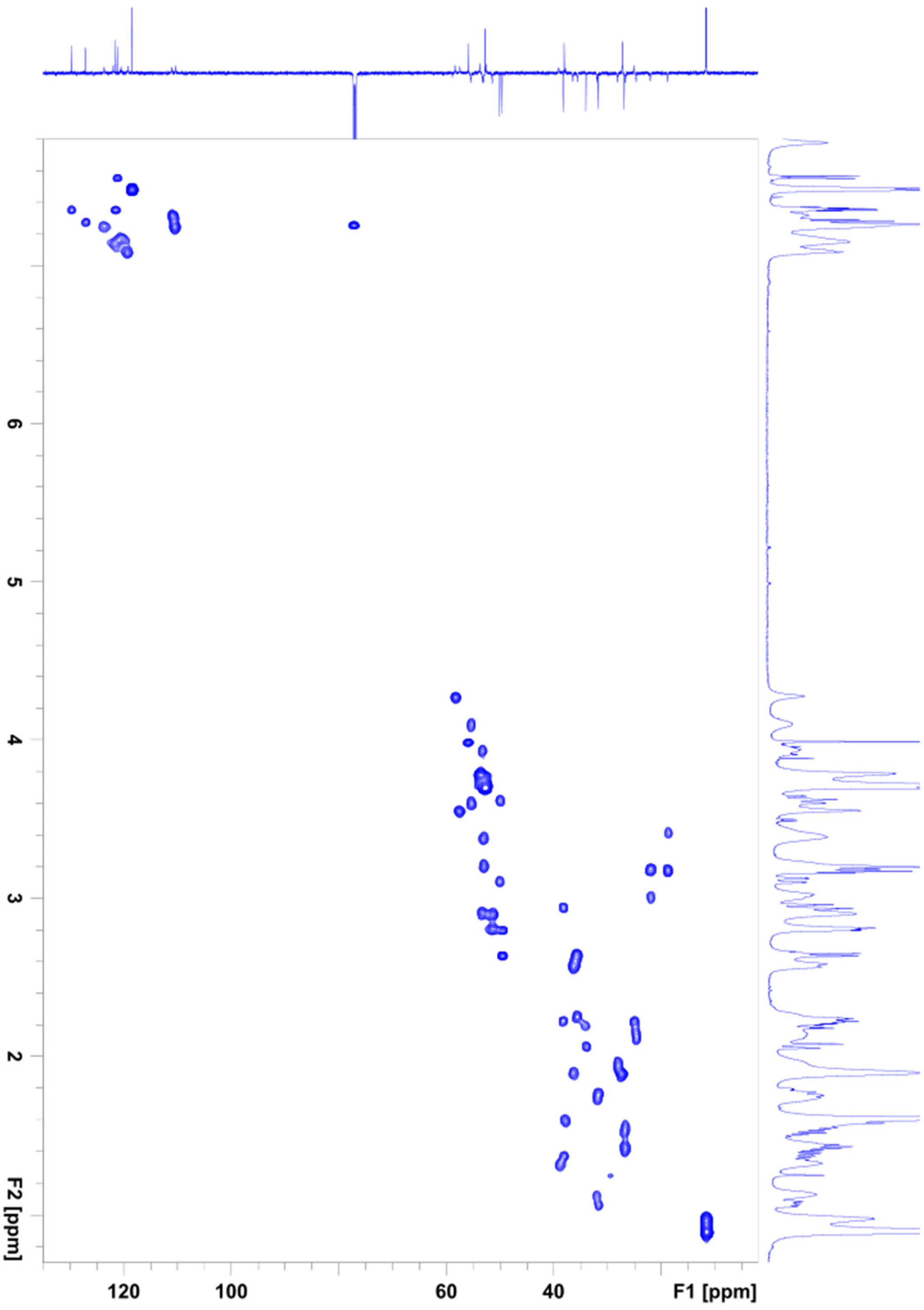


**Figure S35**: HSQC spectrum of 3,7-coronaridine isoindolenine (**1**) in $CDCl_3$.

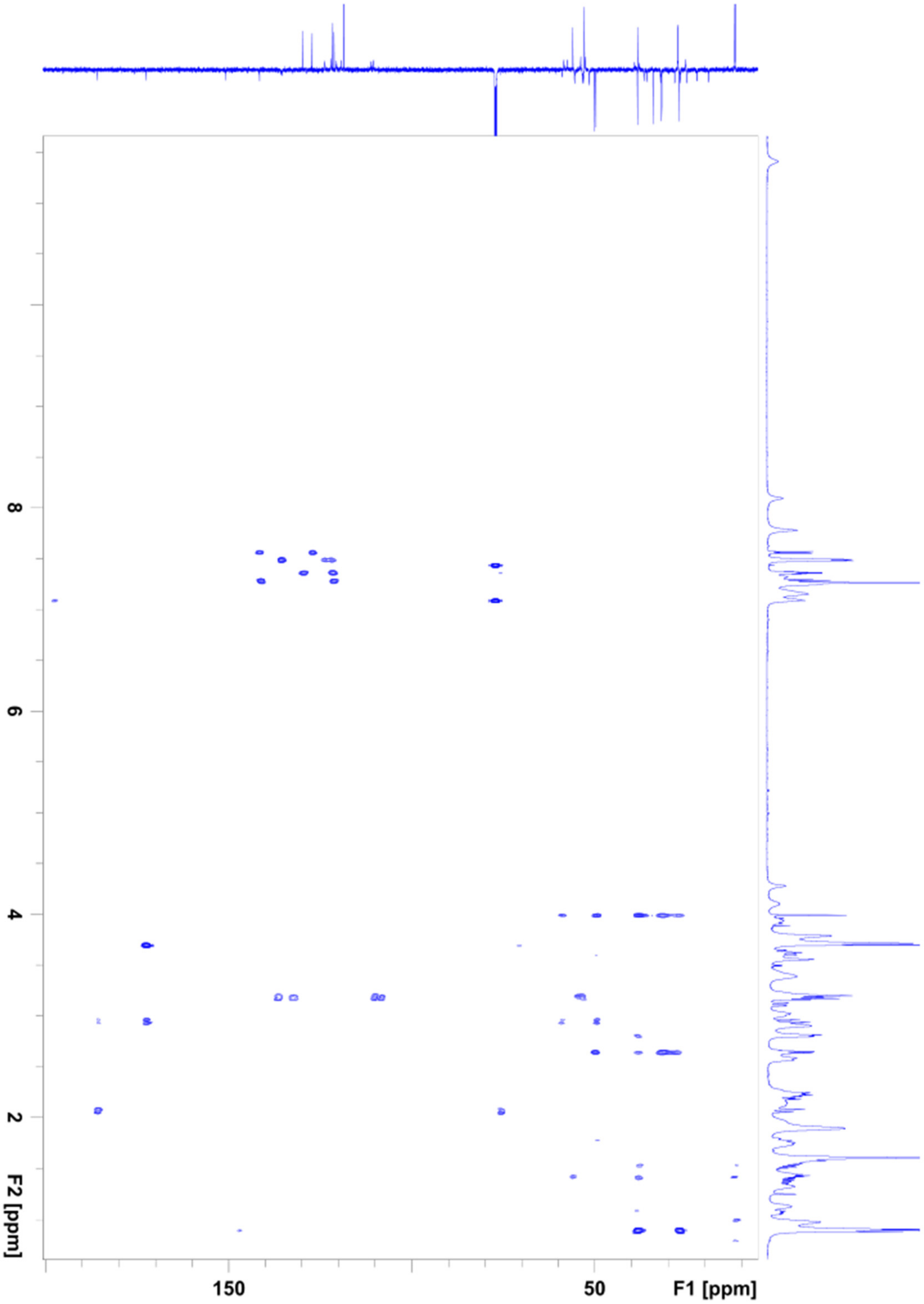


**Figure S36**: HMBC spectrum of 3,7-coronaridine isoindolenine (**1**) in $CDCl_3$.

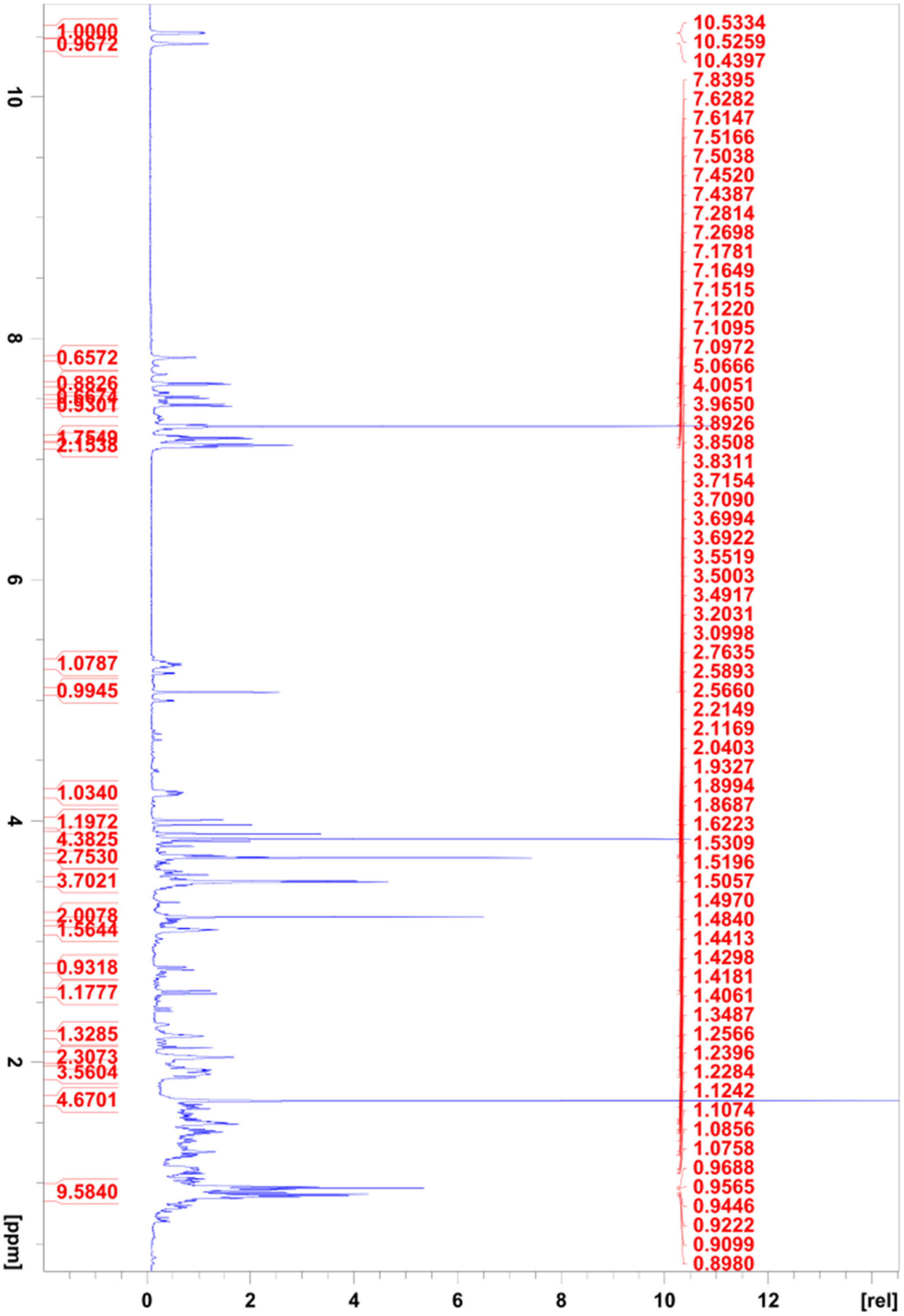


**Figure 37**: $^{1}$H NMR of coronaridine 3,4-iminium (**2**) in $CDCl_3$.

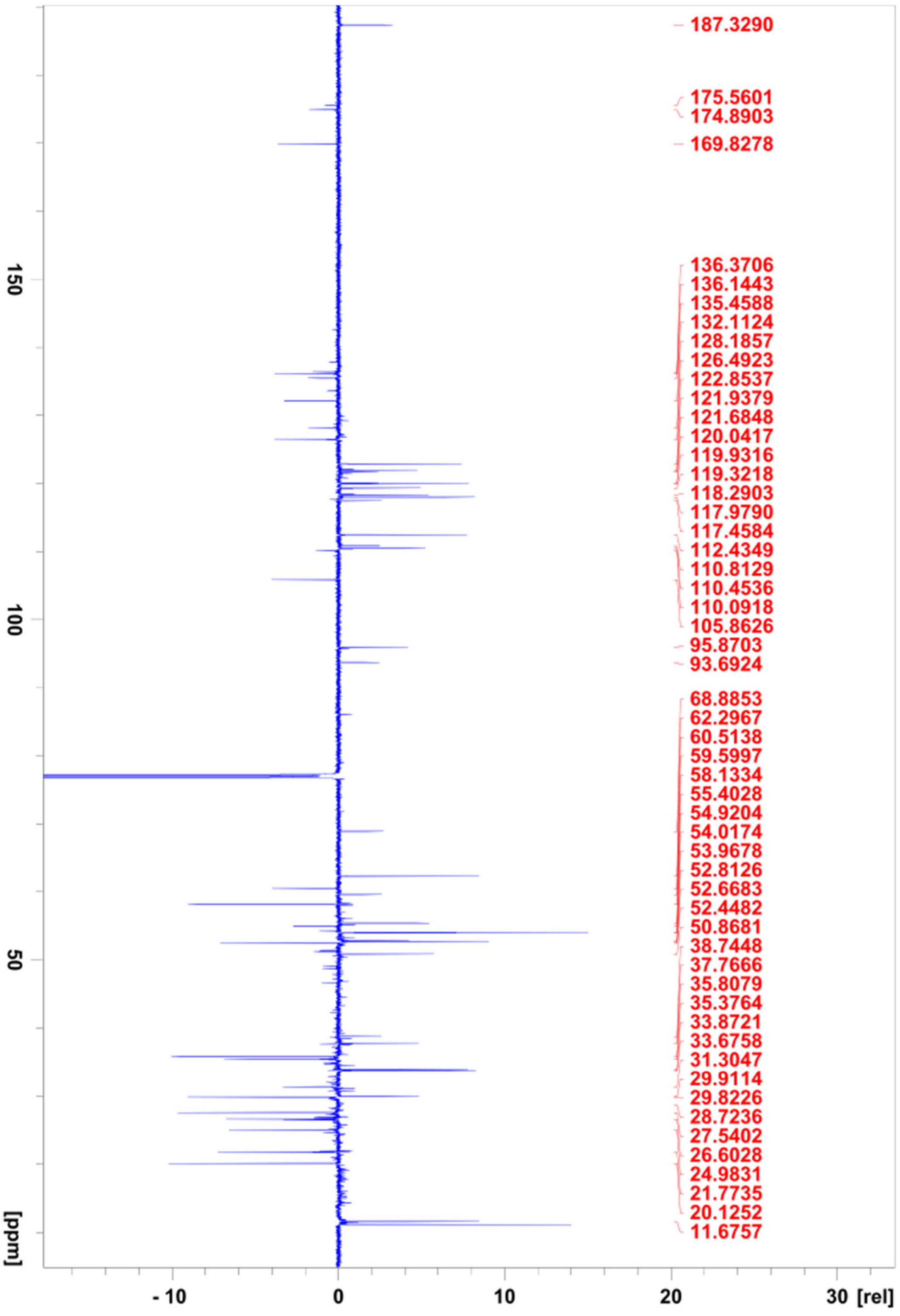


**Figure S38**: $^{13}$C NMR of coronaridine 3,4-iminium (**2**) in $CDCl_3$.

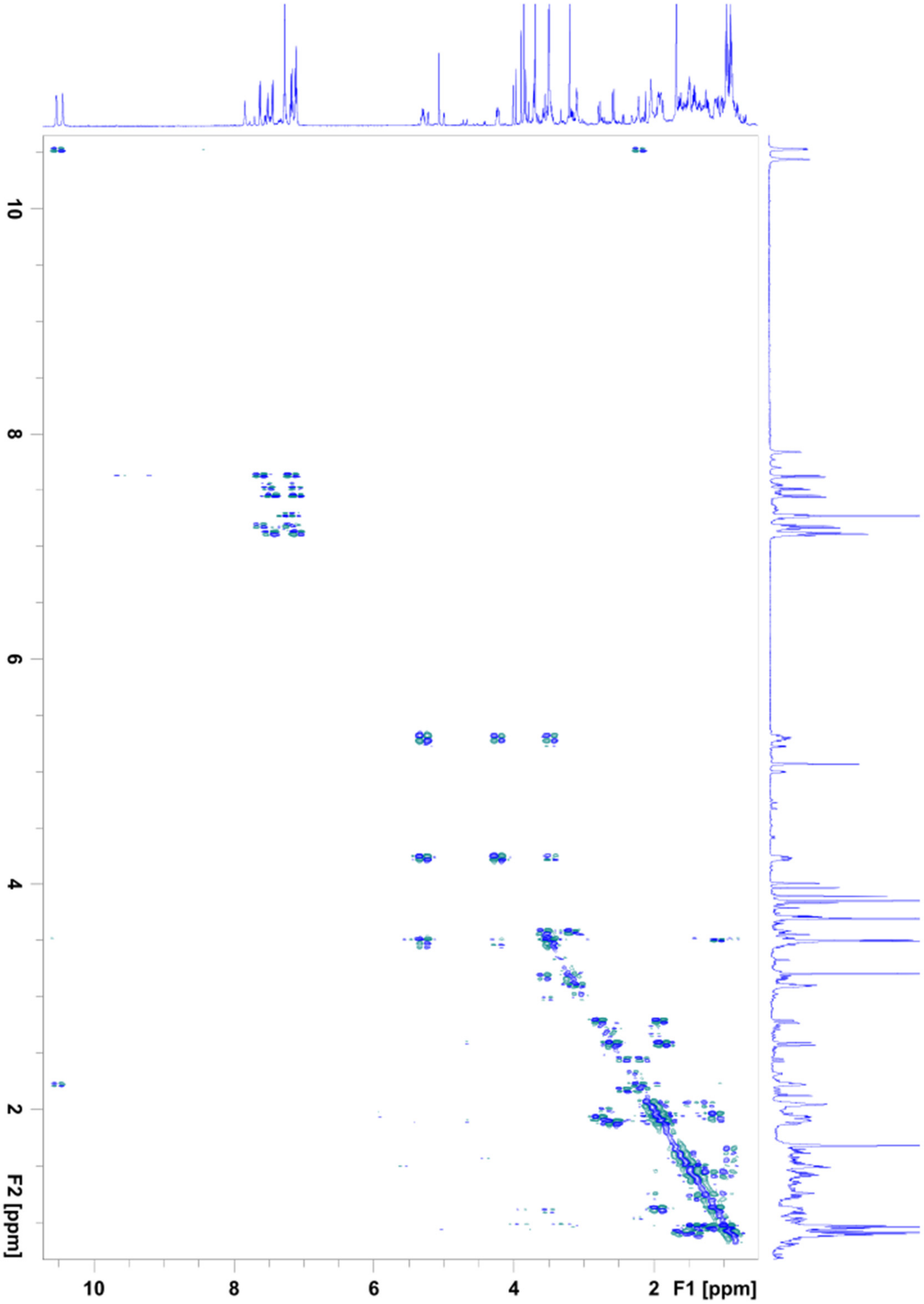


**Figure S39**: COSY spectrum of coronaridine 3,4-iminium (**2**) in $CDCl_3$.

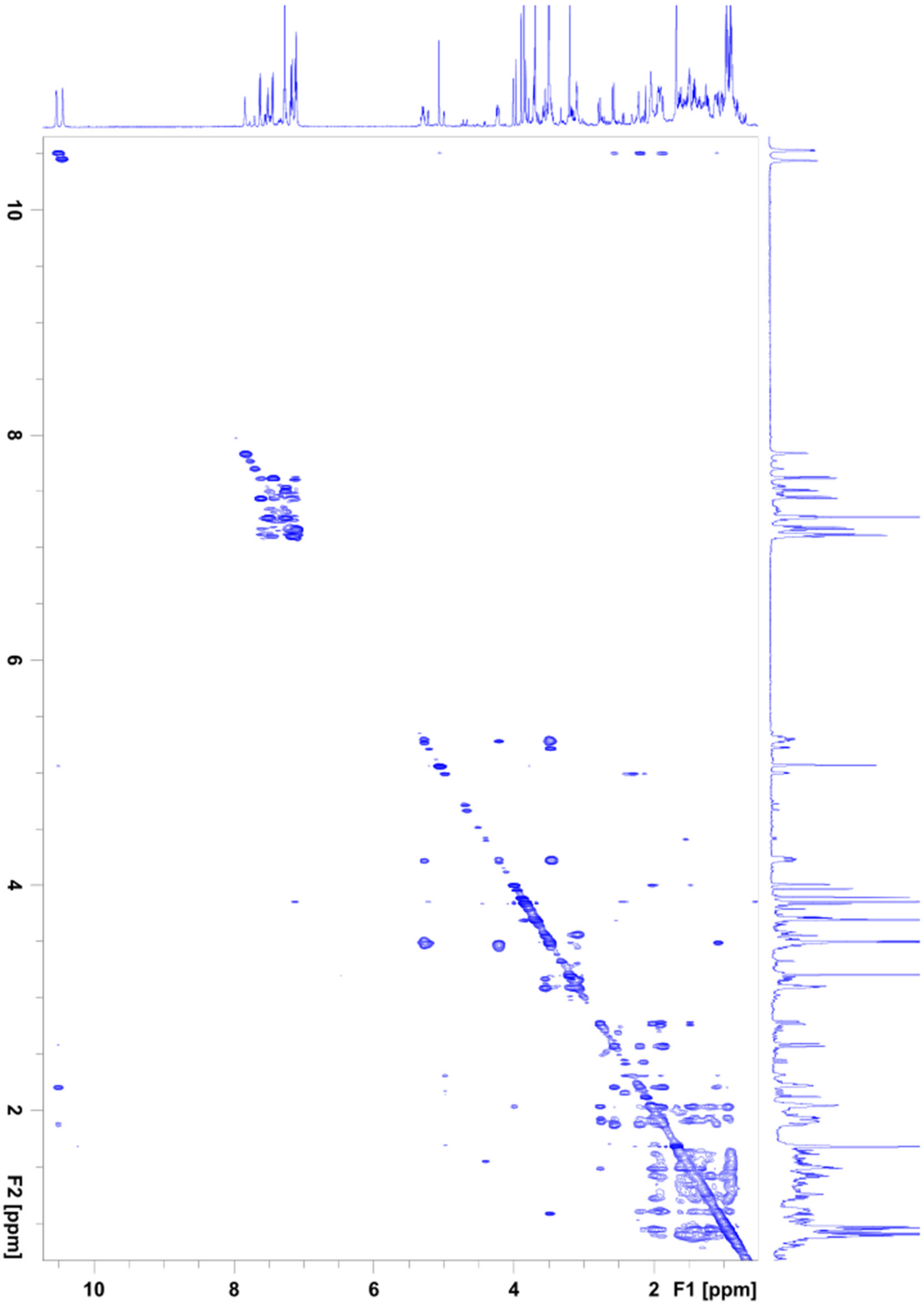


**Figure S40**: TOCSY spectrum of coronaridine 3,4-iminium (**2**) in $CDCl_3$.

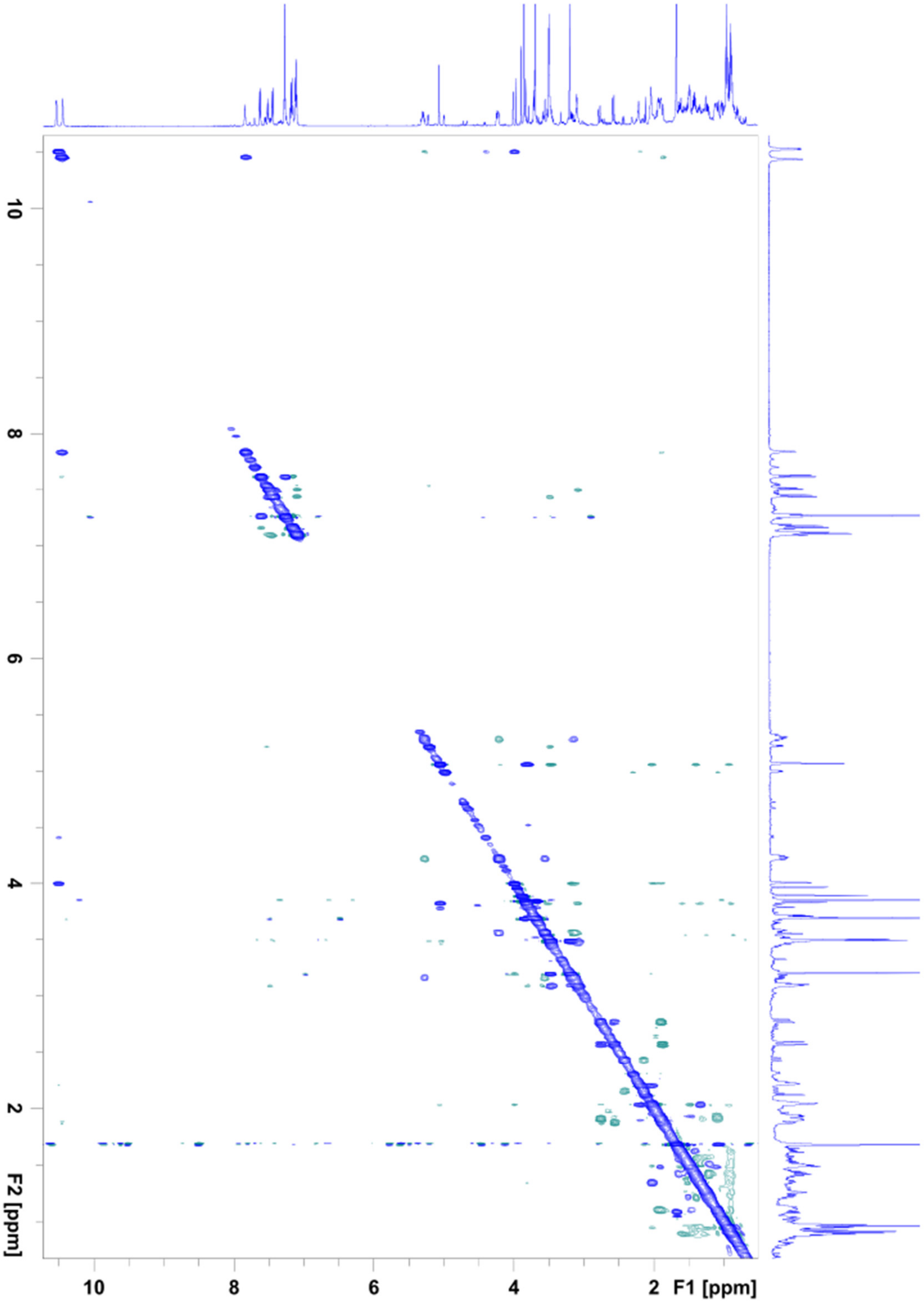


**Figure S41**: NOESY spectrum of coronaridine 3,4-iminium (**2**) in $CDCl_3$.

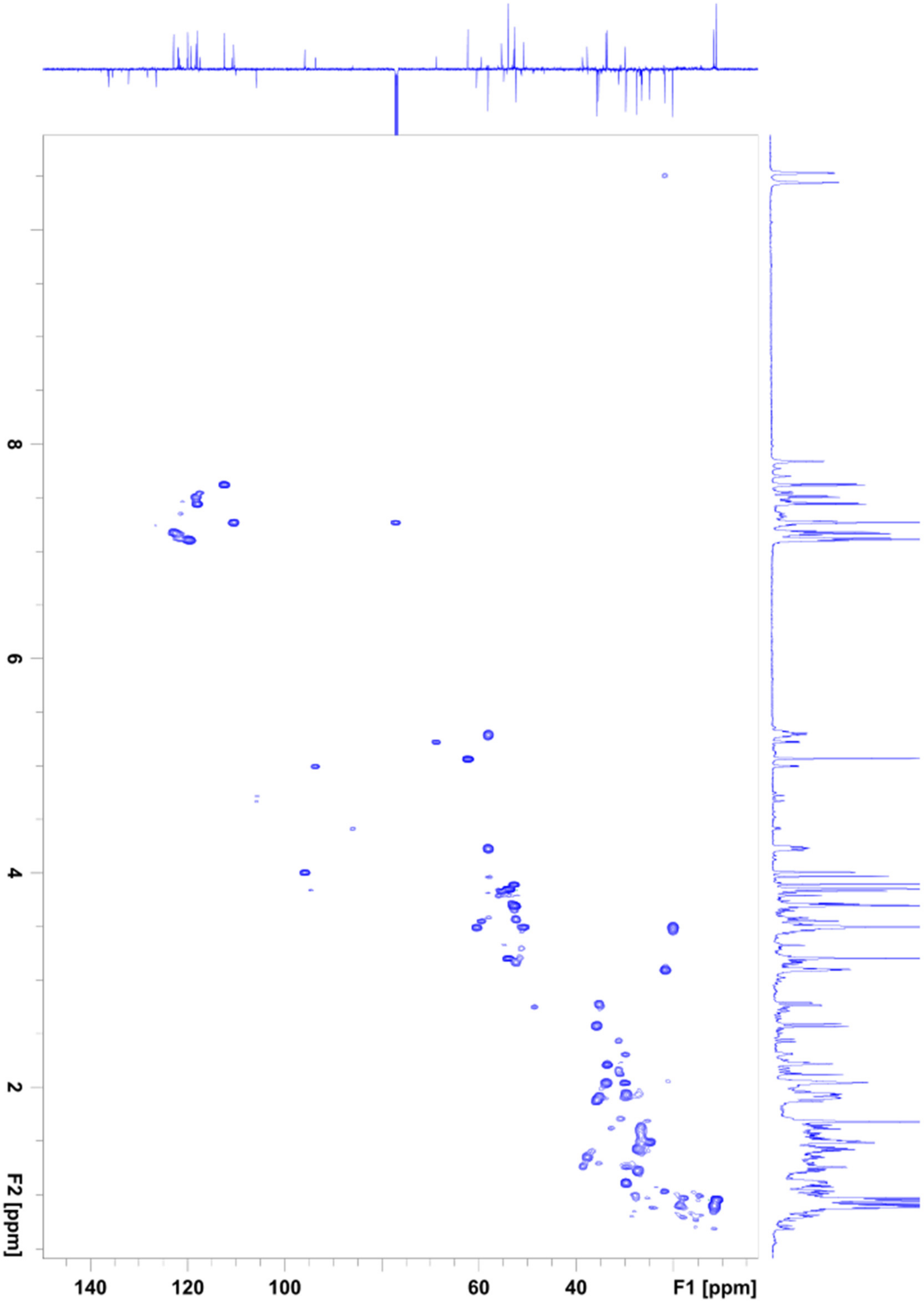


**Figure S42**: HSQC spectrum of coronaridine 3,4-iminium (**2**) in $CDCl_3$.

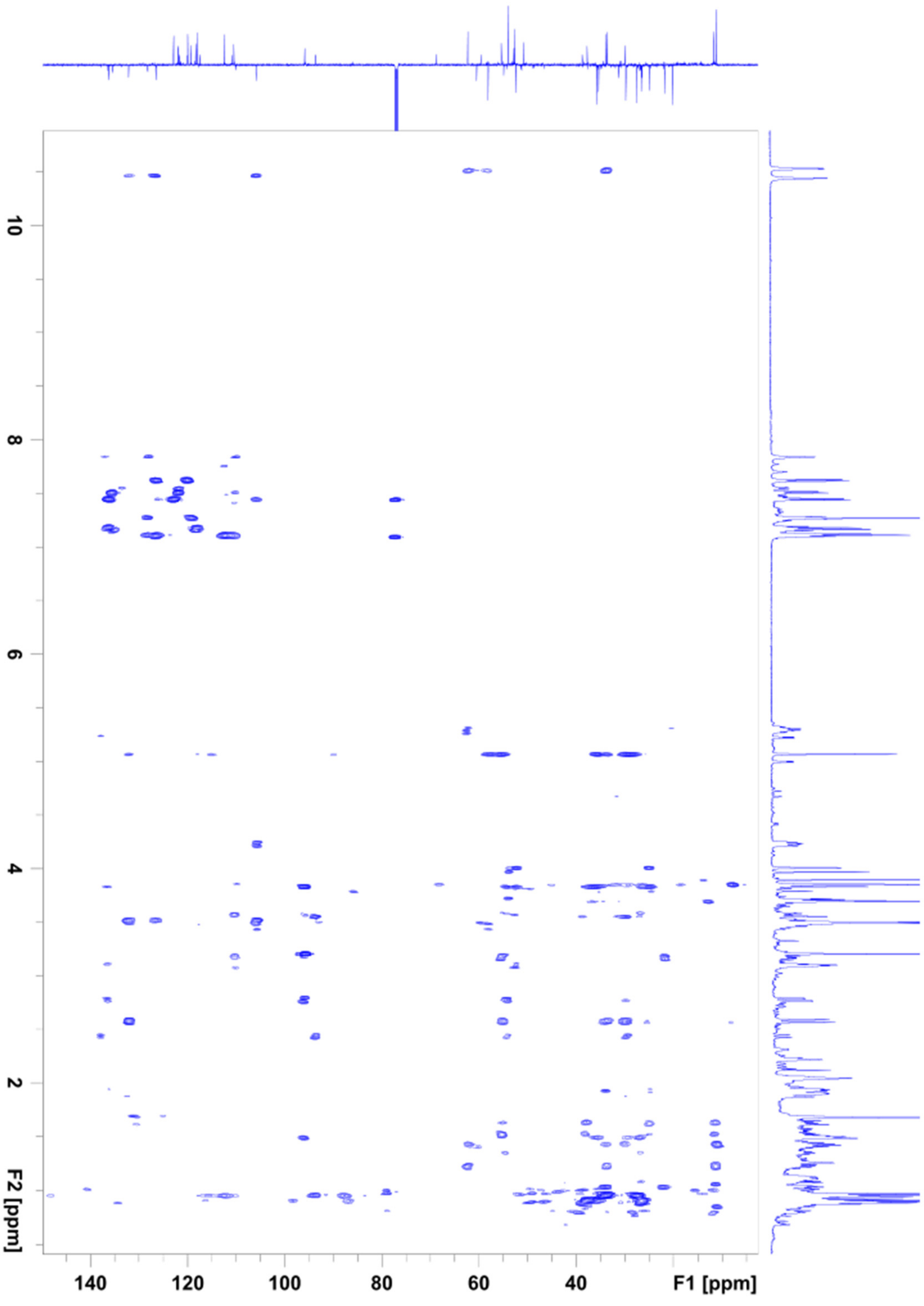


**Figure S43**: HMBC spectrum of coronaridine 3,4-iminium (**2**) in $CDCl_3$.

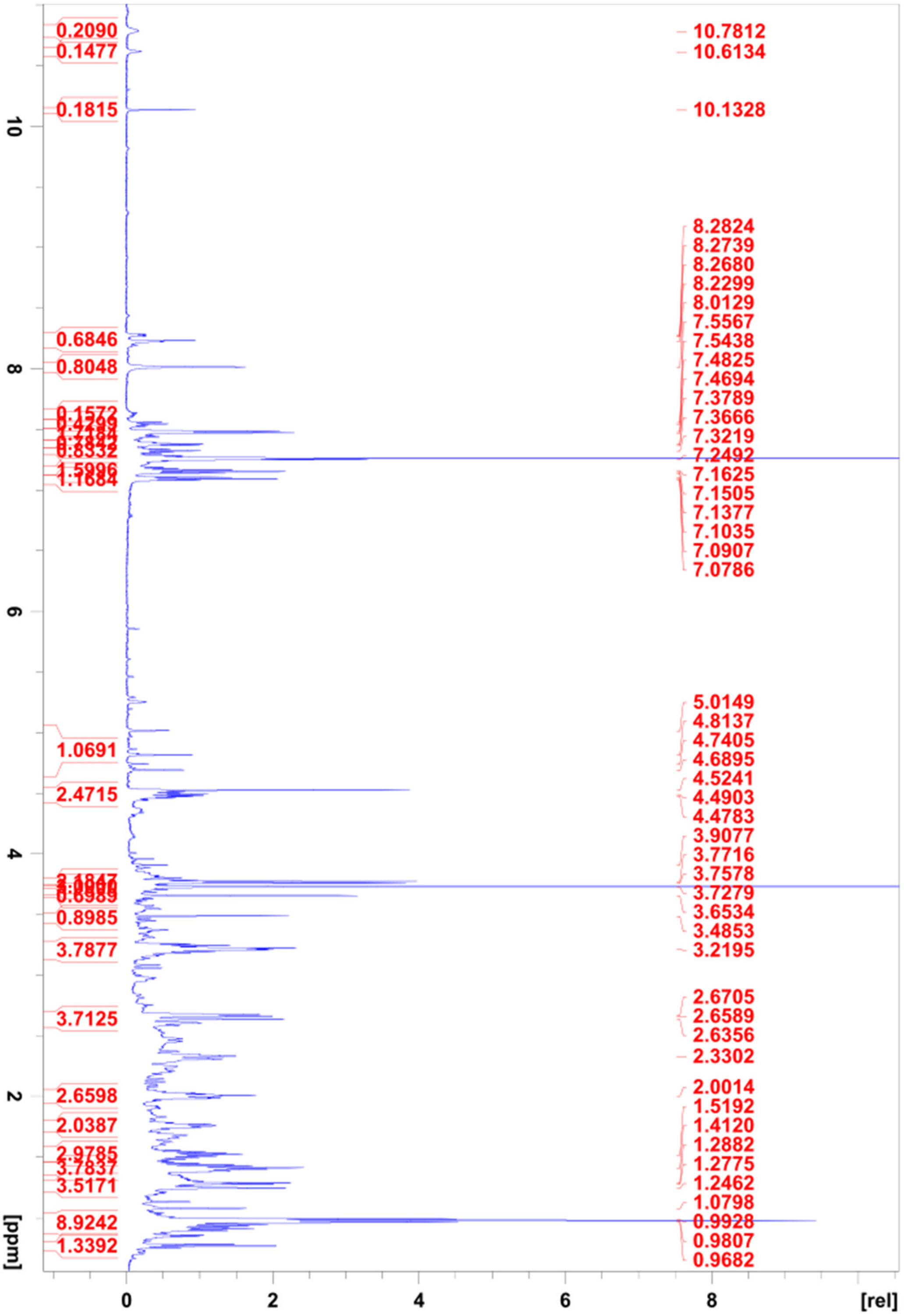


**Figure S44**: $^{1}H$ NMR of 3-oxocoronaridine (**3**) in $CDCl_3$.

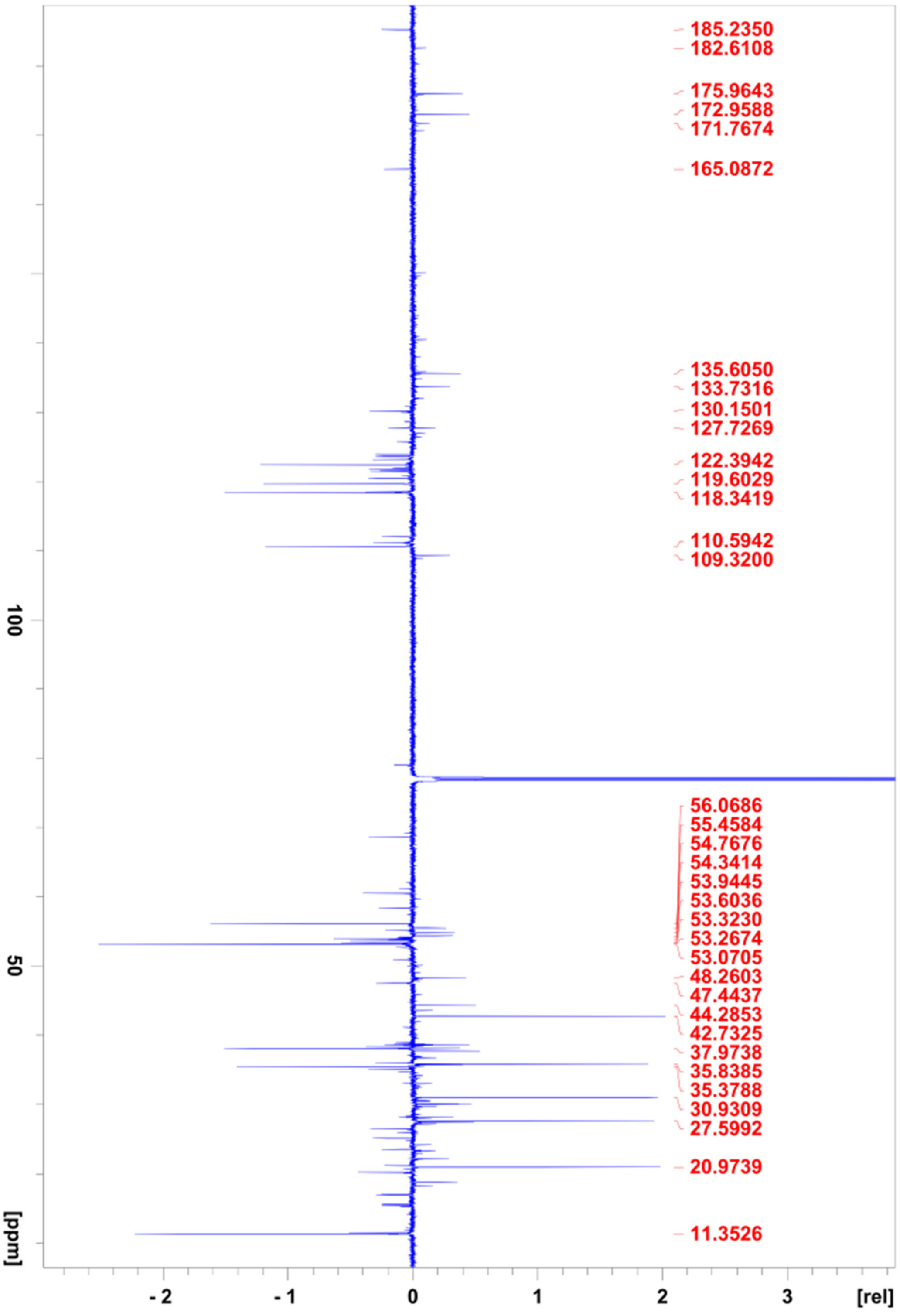


**Figure S45**: $^{13}C$ NMR of 3-oxocoronaridine (**3**) in $CDCl_3$.

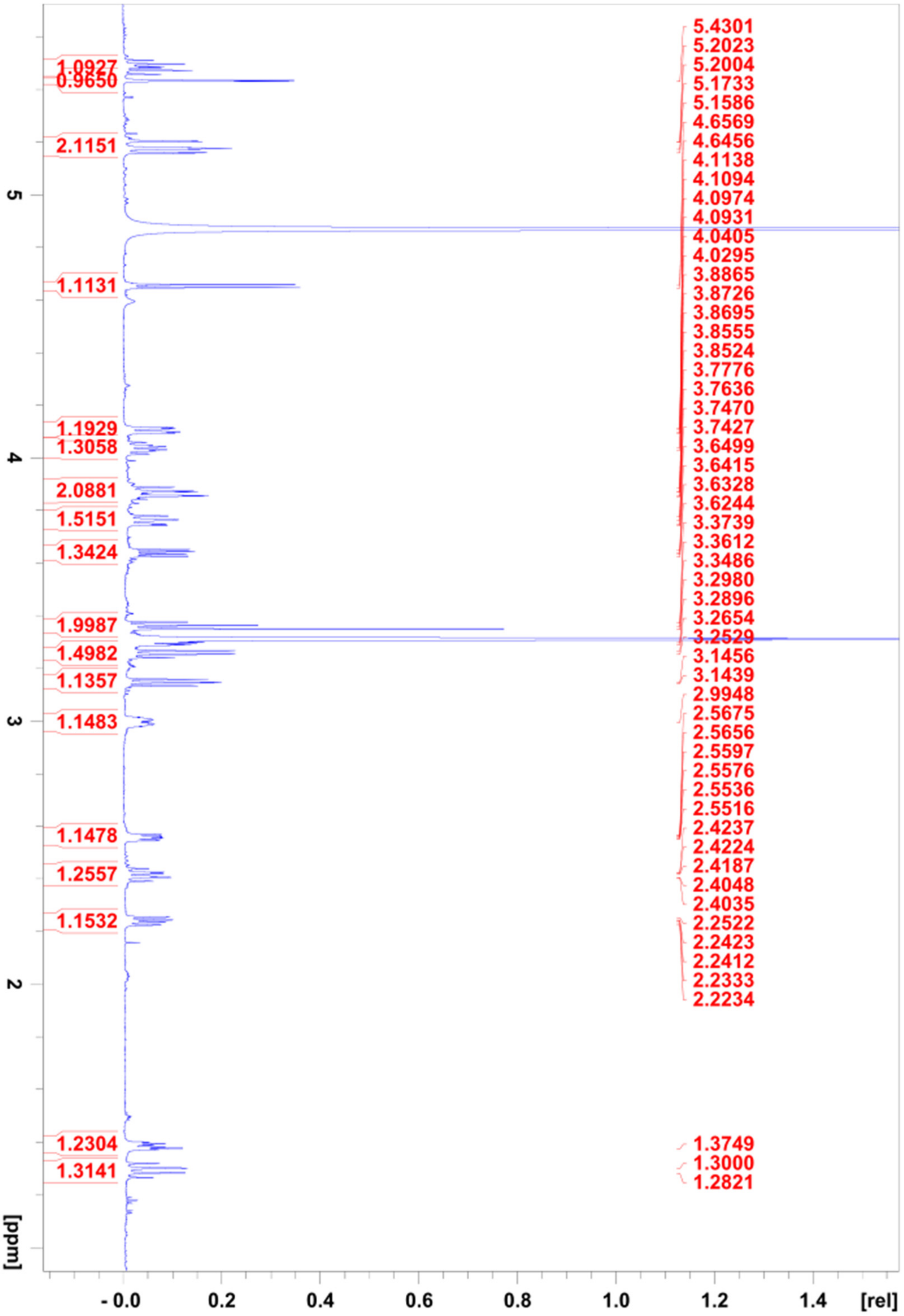


**Figure S46**: $^{1}H$ NMR of javaniside (**4**) in $CD_3OD$.

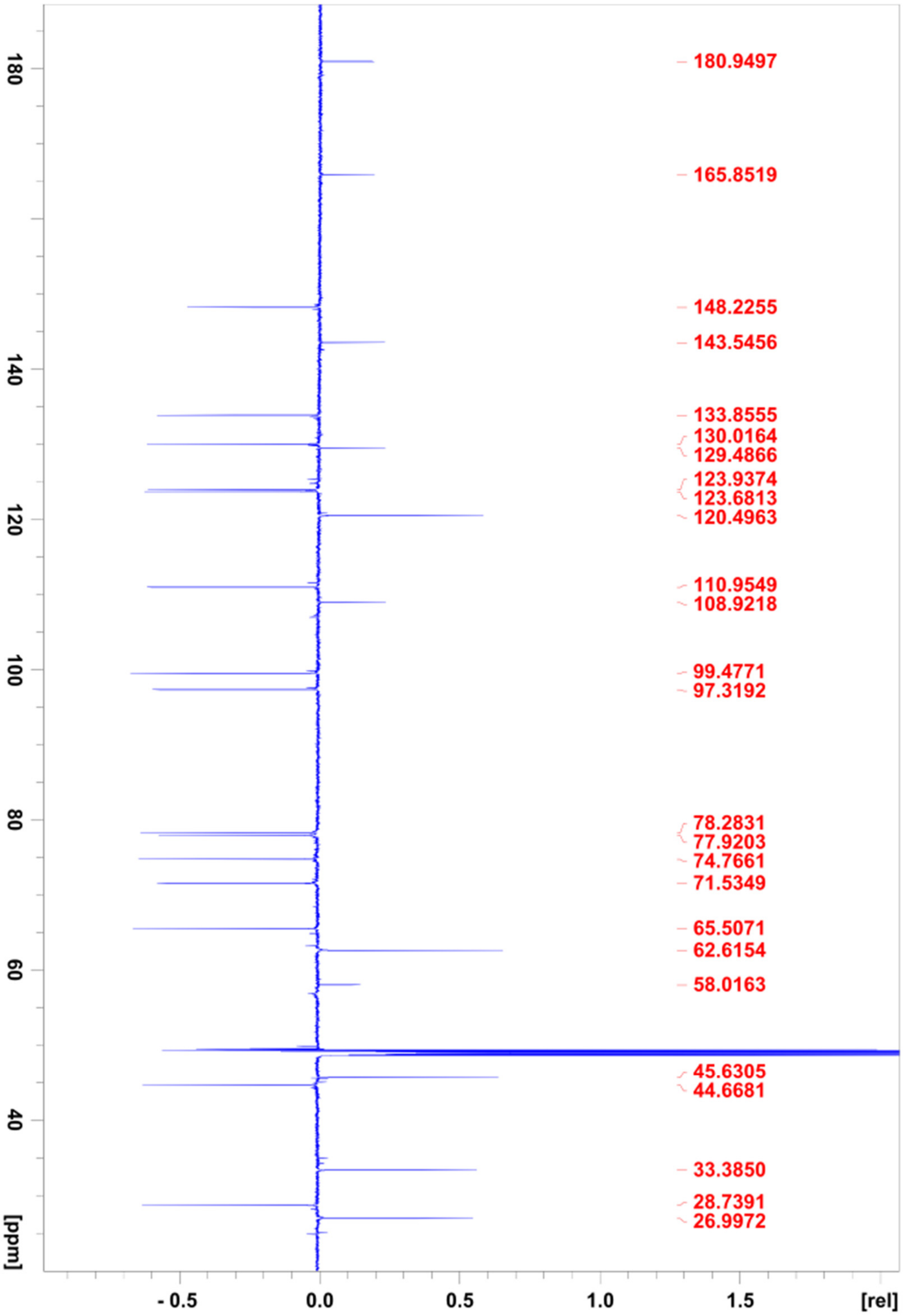


**Figure S47**: $^{13}$C NMR of javaniside (**4**) in $CD_3OD$.

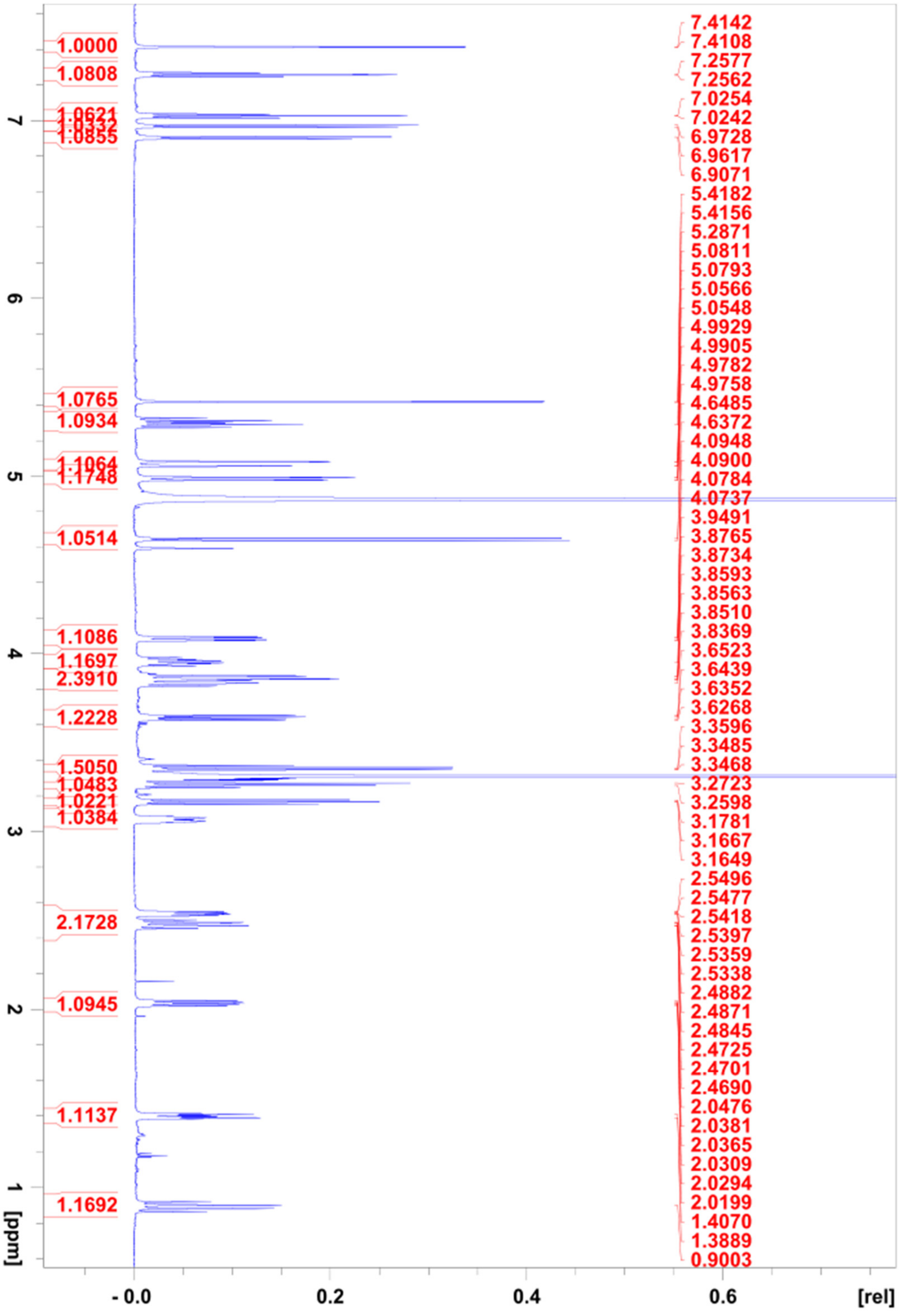


**Figure S48**: $^{1}$H NMR of 7-epi-javaniside (**5**) in $CD_3OD$.

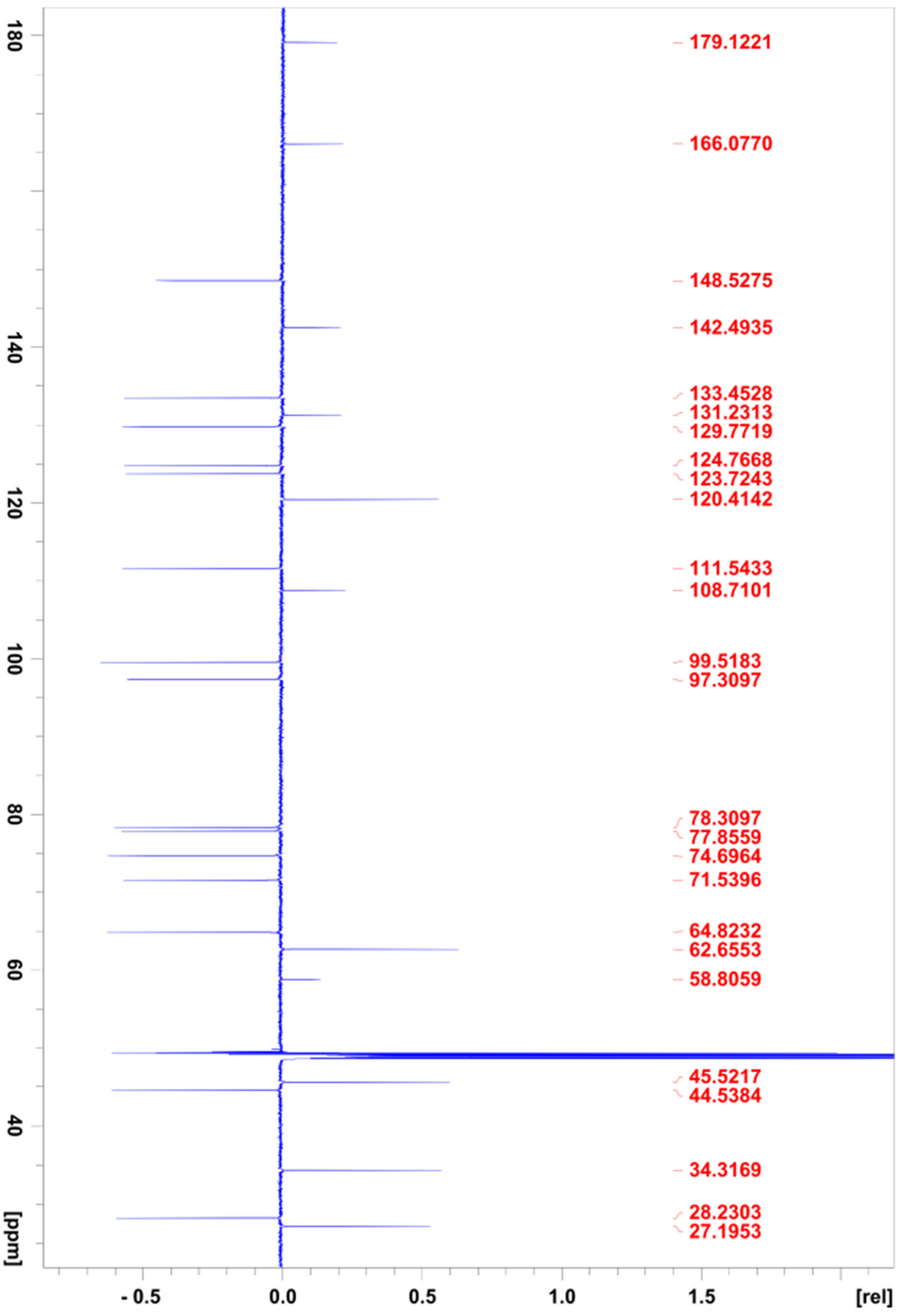


**Figure S49**: $^{13}C$ NMR of 7-epi-javaniside (**5**) in $CD_3OD$.

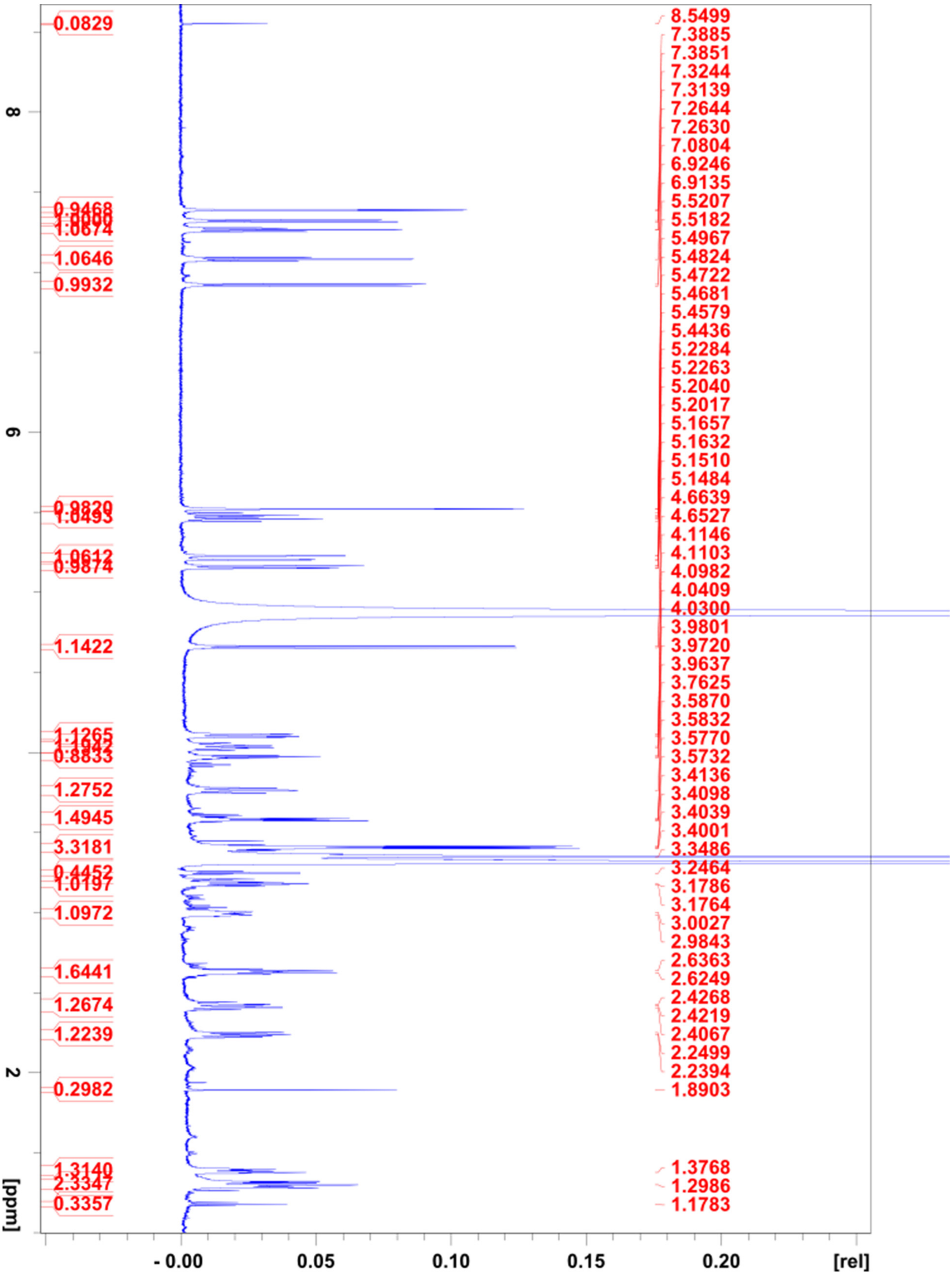


**Figure S50**: $^{1}$H NMR of javanuronic acid (**6**) in $CD_3OD$.

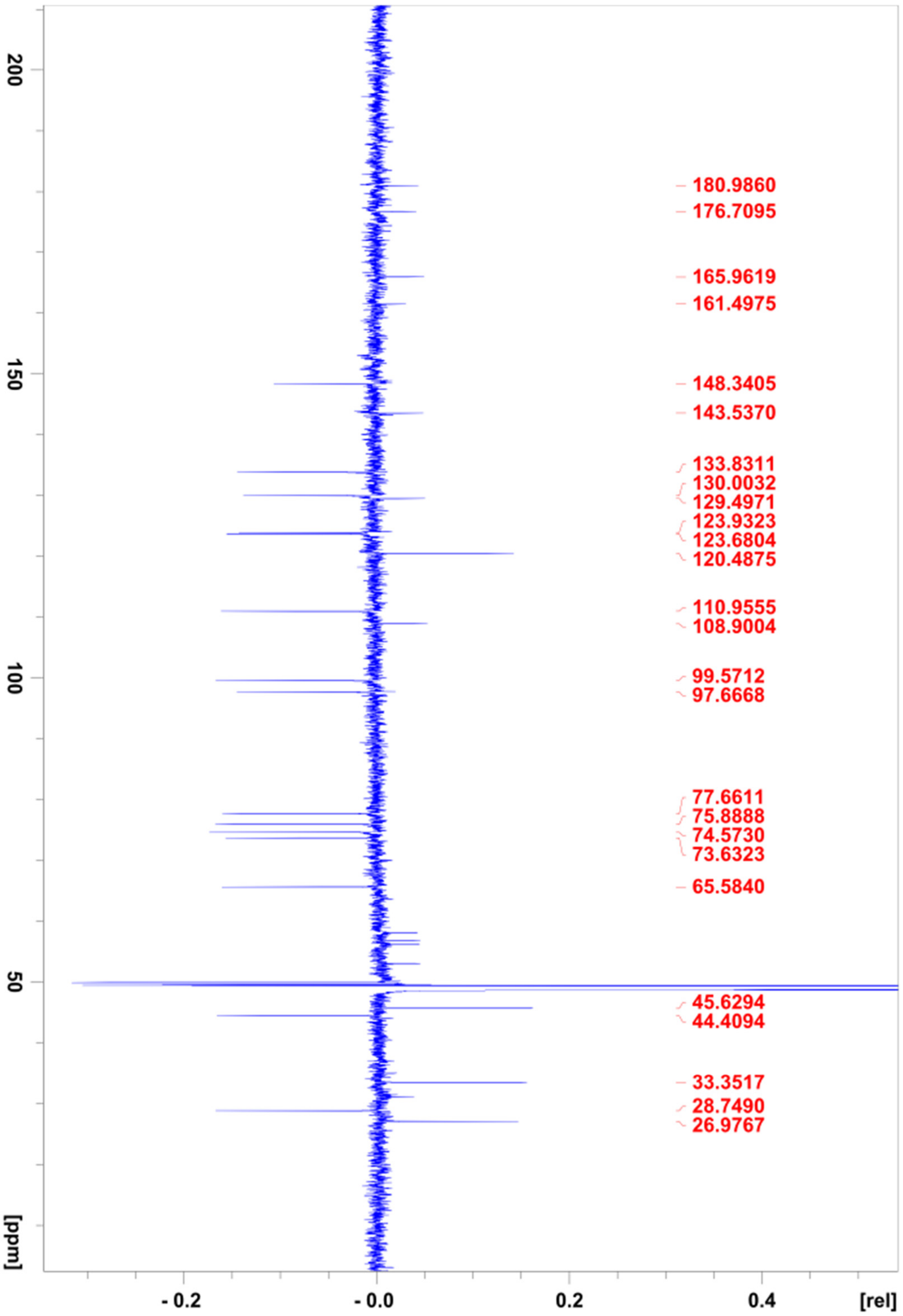


**Figure S51**: $^{13}$C NMR of javanuronic acid (**6**) in $CD_3OD$.

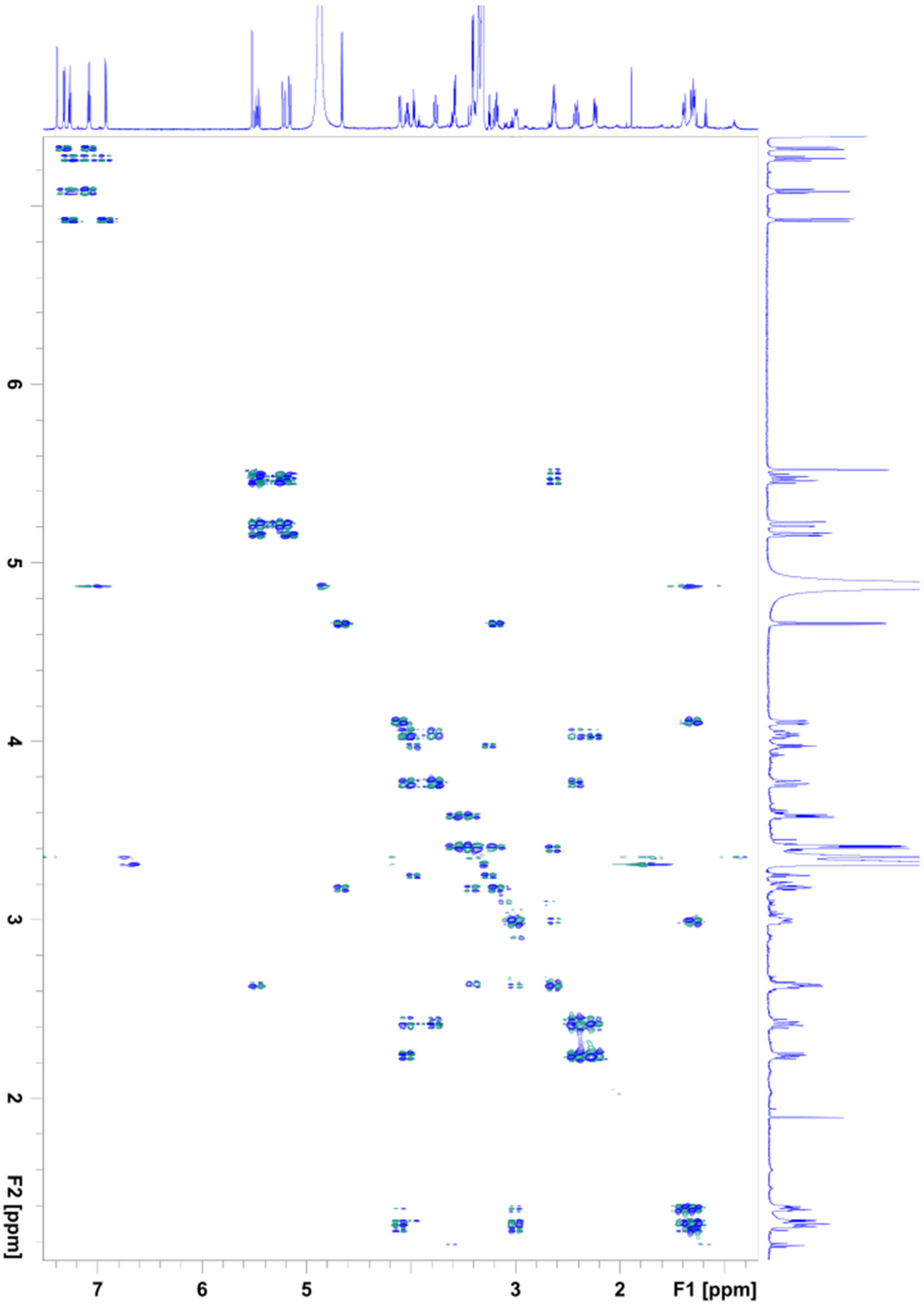


**Figure S52**: COSY spectrum of javanuronic acid (**6**) in $CD_3OD$.

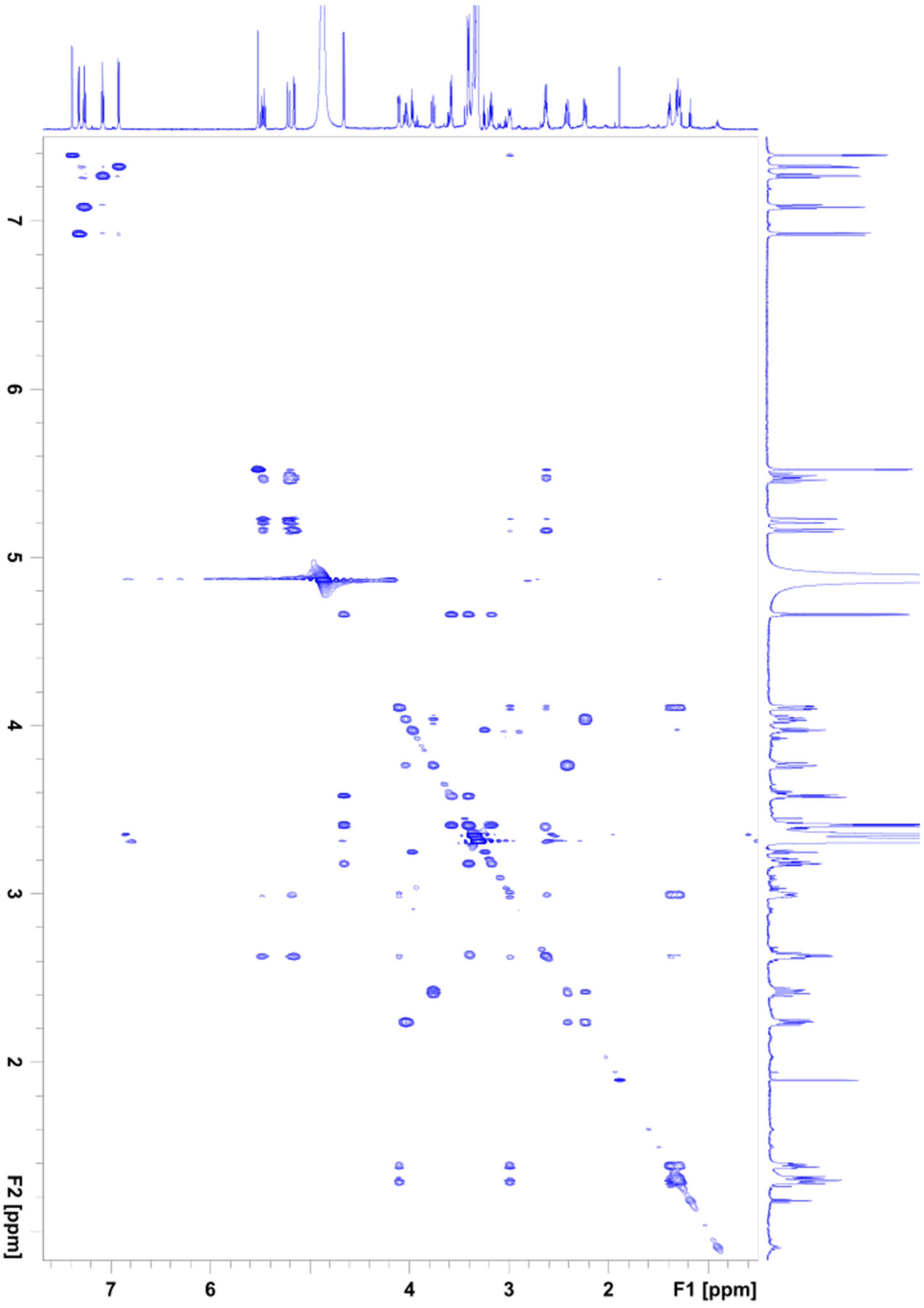


**Figure S53**: TOCSY spectrum of javanuronic acid (**6**) in $CD_3OD$.

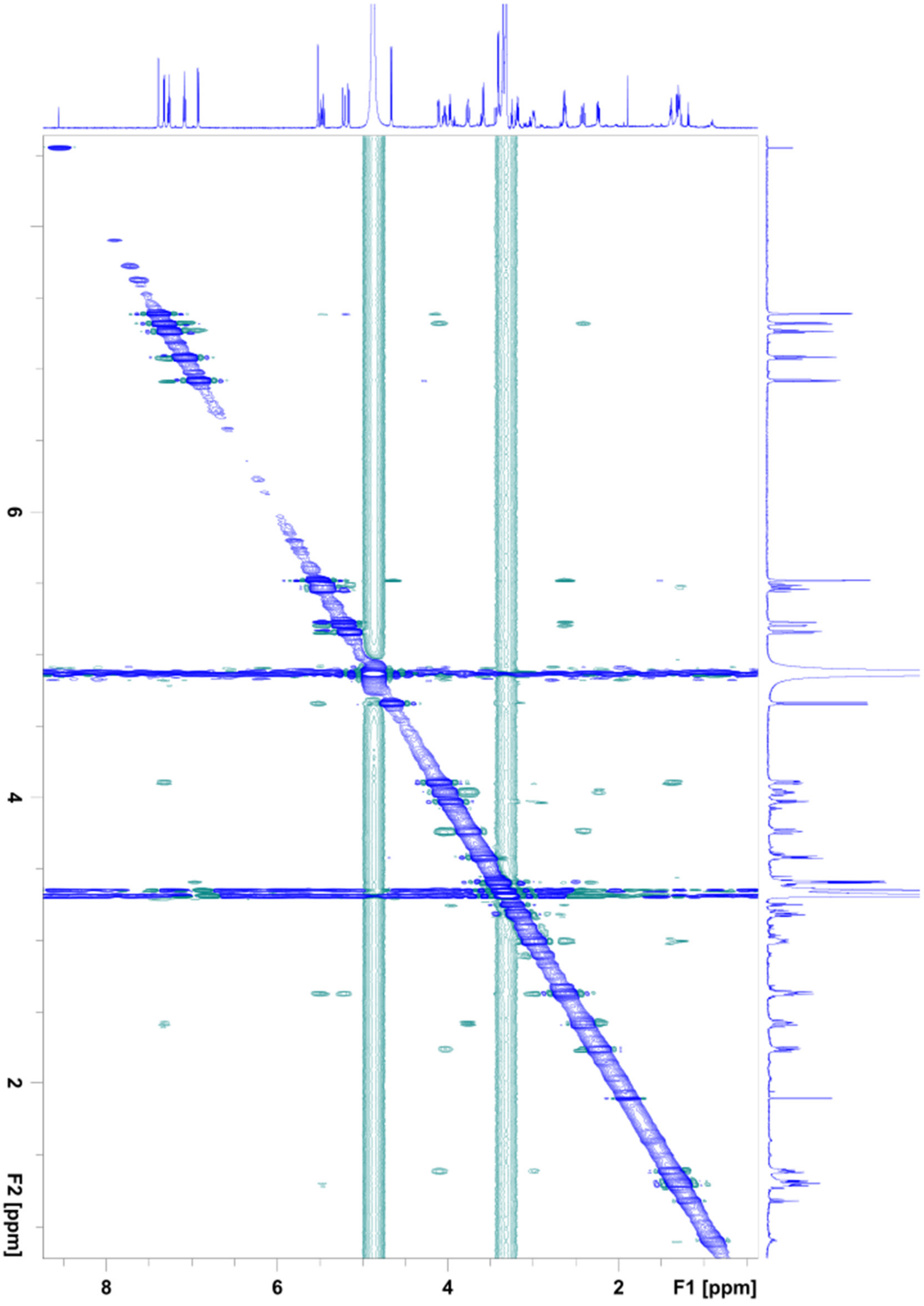


**Figure S54**: NOESY spectrum of javanuronic acid (**6**) in $CD_3OD$.

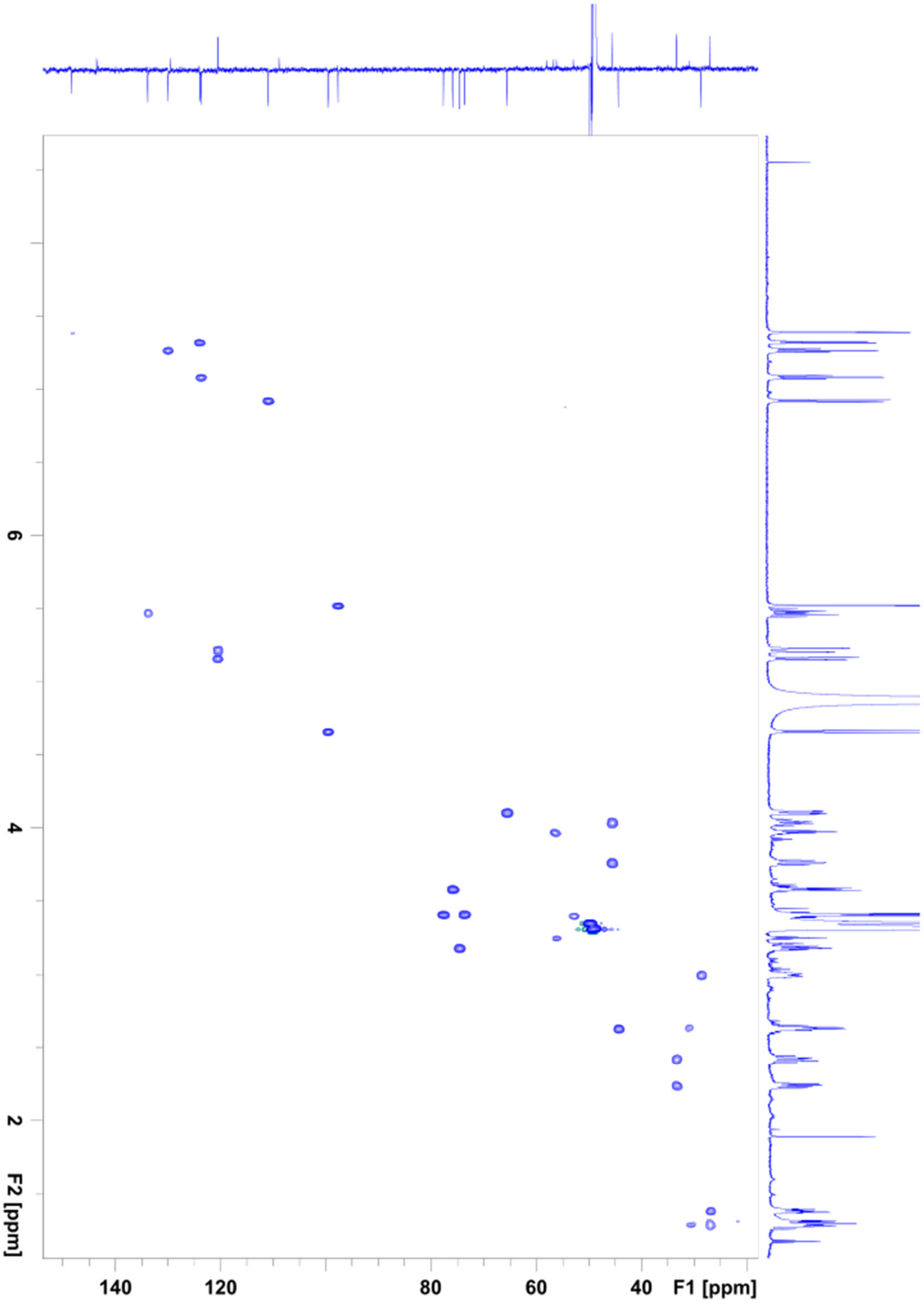


**Figure S55**: HSQC spectrum of javanuronic acid (**6**) in $CD_3OD$.

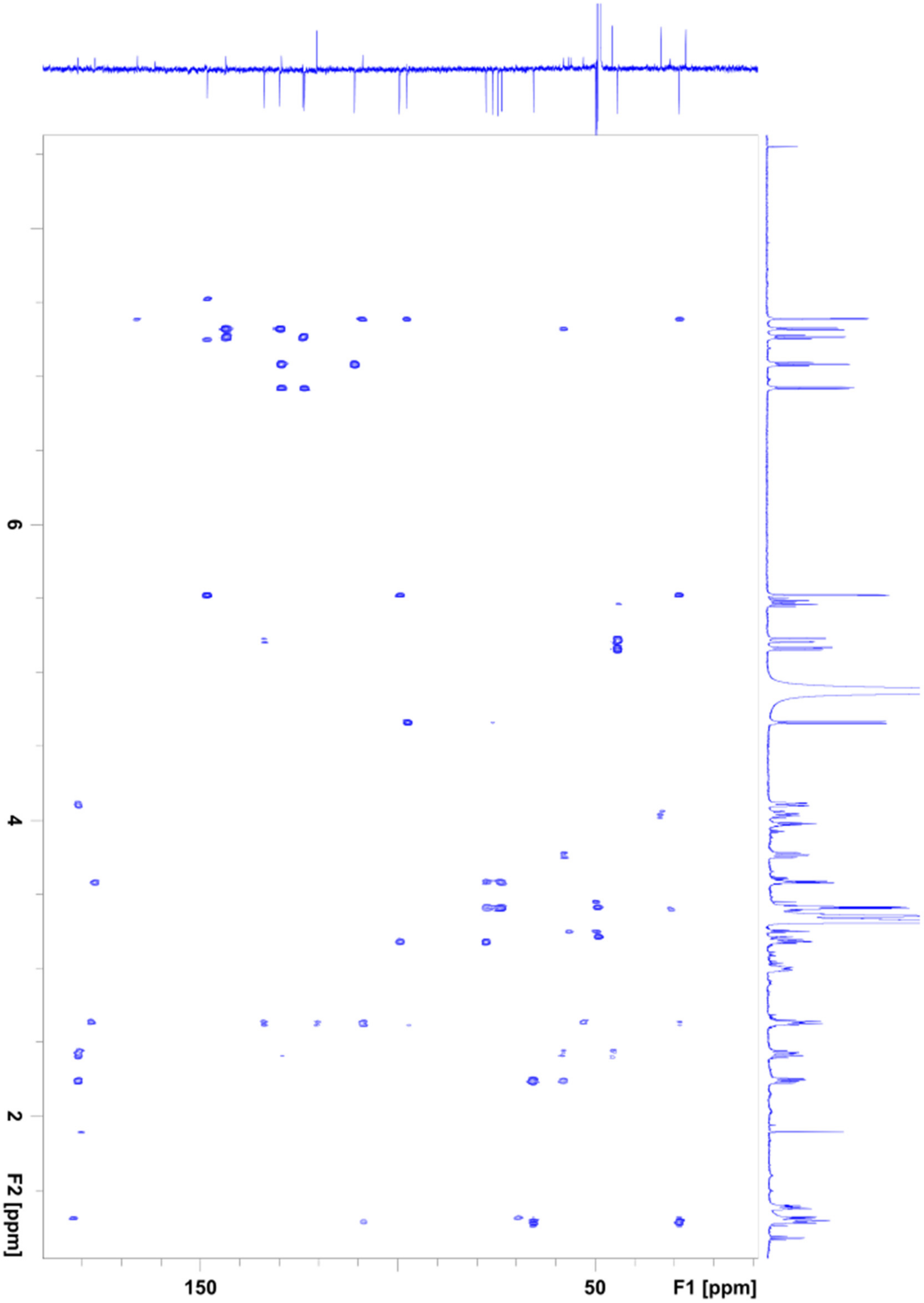


**Figure S56**: HMBC spectrum of javanuronic acid (**6**) in $CD_3OD$.

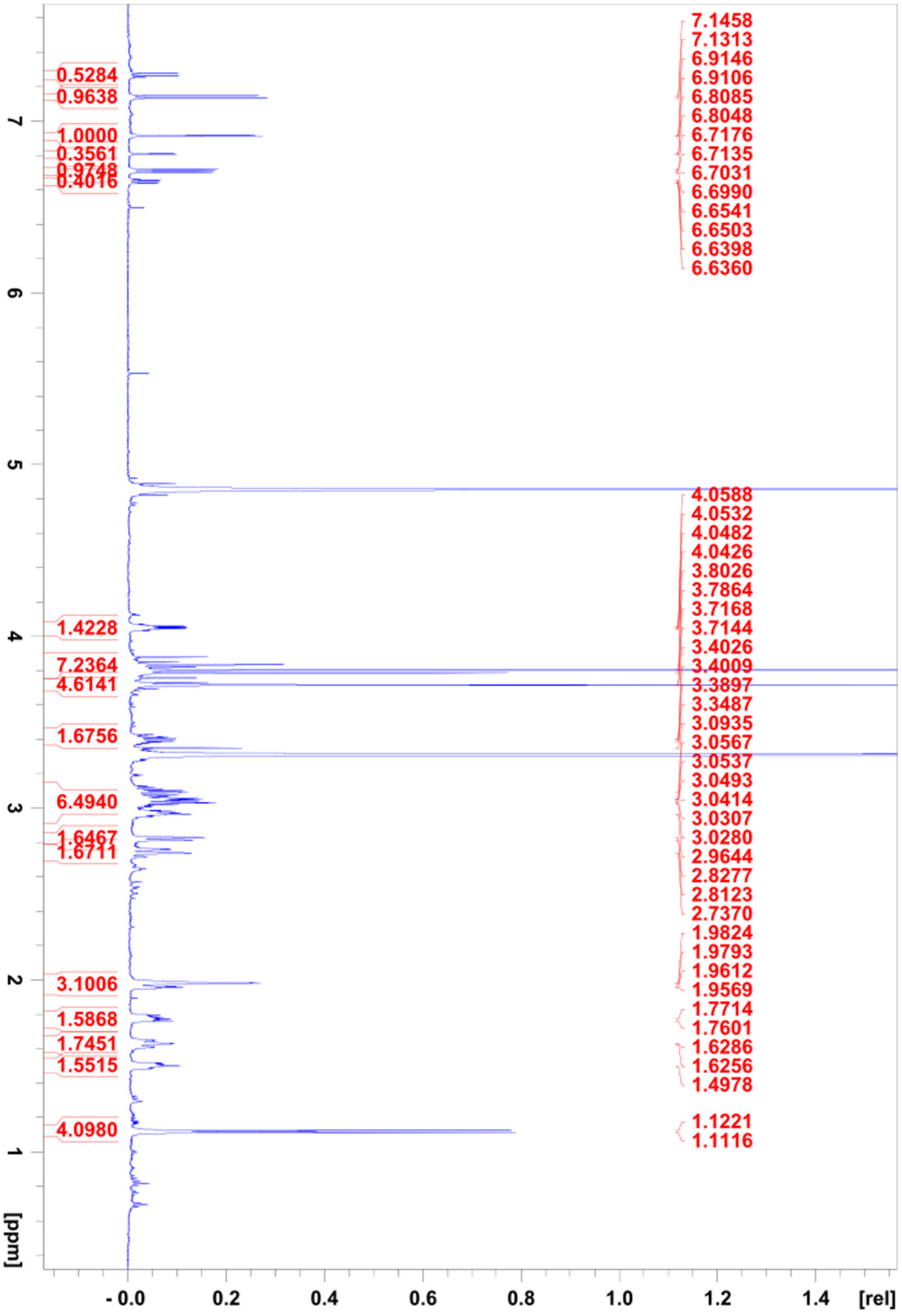


**Figure S57**: $^{1}$H NMR of vocacristine (**7**) in $CD_3OD$.

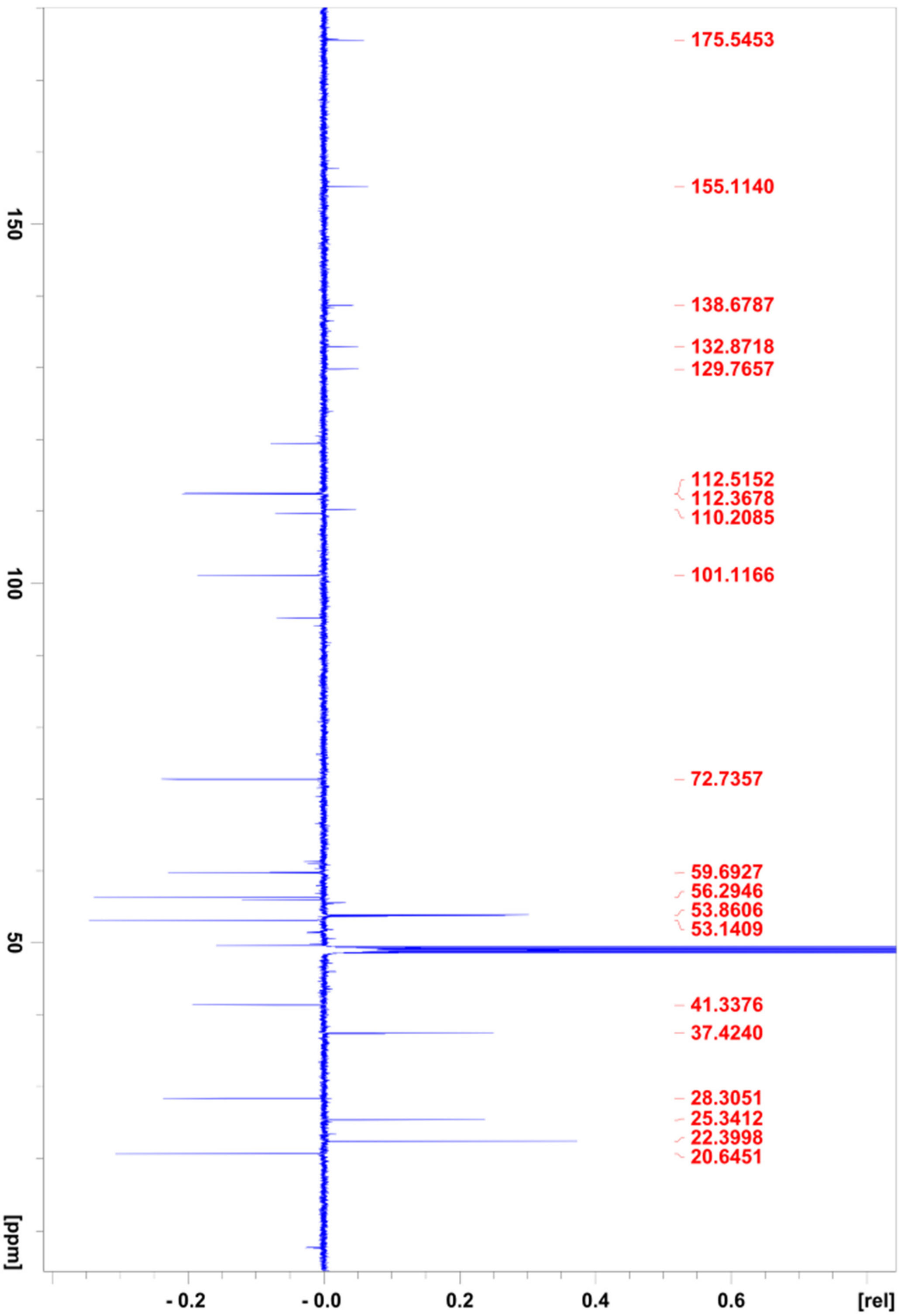


**Figure S58:** $^{13}C$ NMR of vocacristine (**7**) in $CD_3OD$.

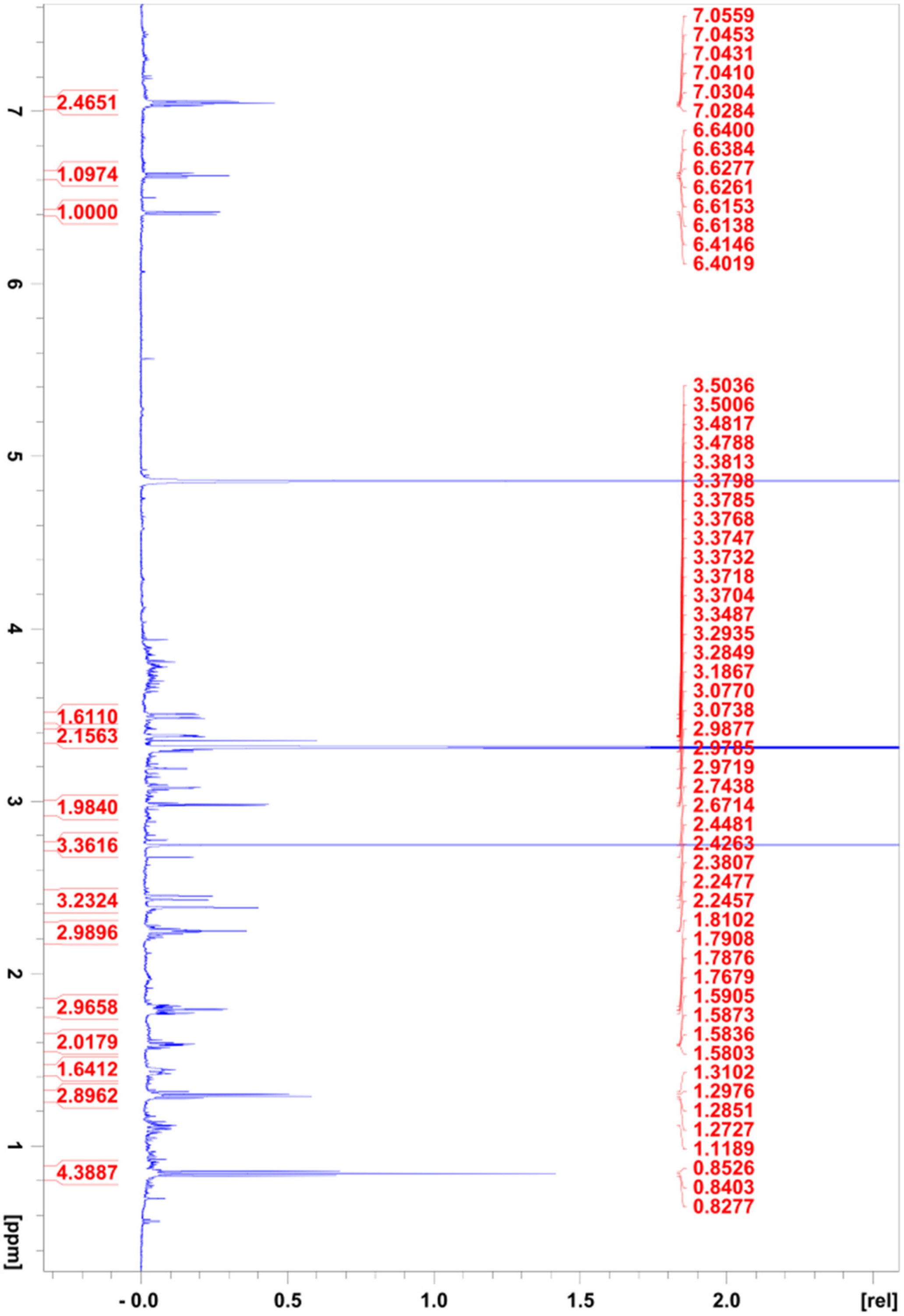


**Figure S59:** $^{1}$H NMR of mehranine (**8**) in $CD_3OD$.

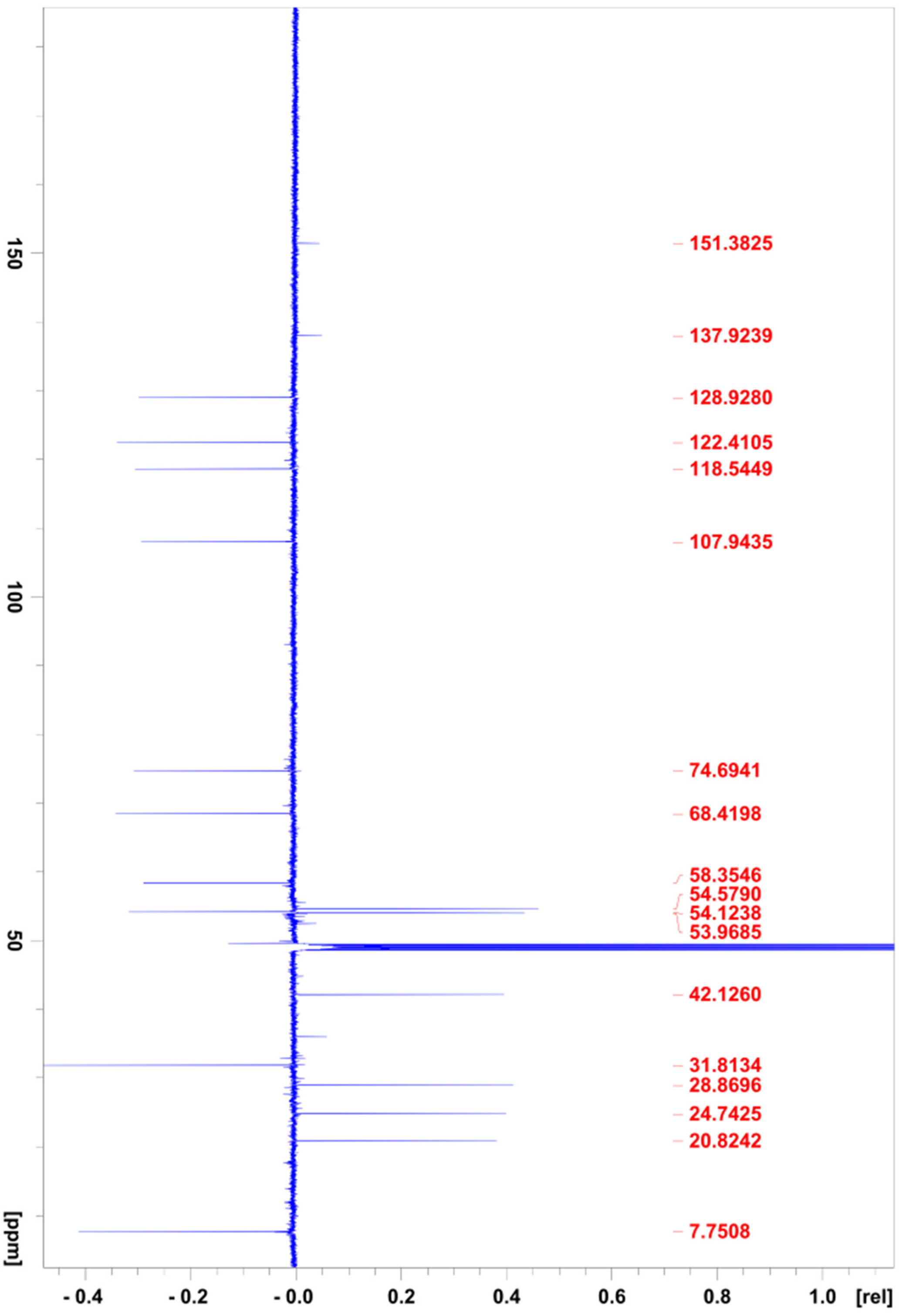


**Figure S60**: $^{13}C$ NMR of mehranine (**8**) in $CD_3OD$.

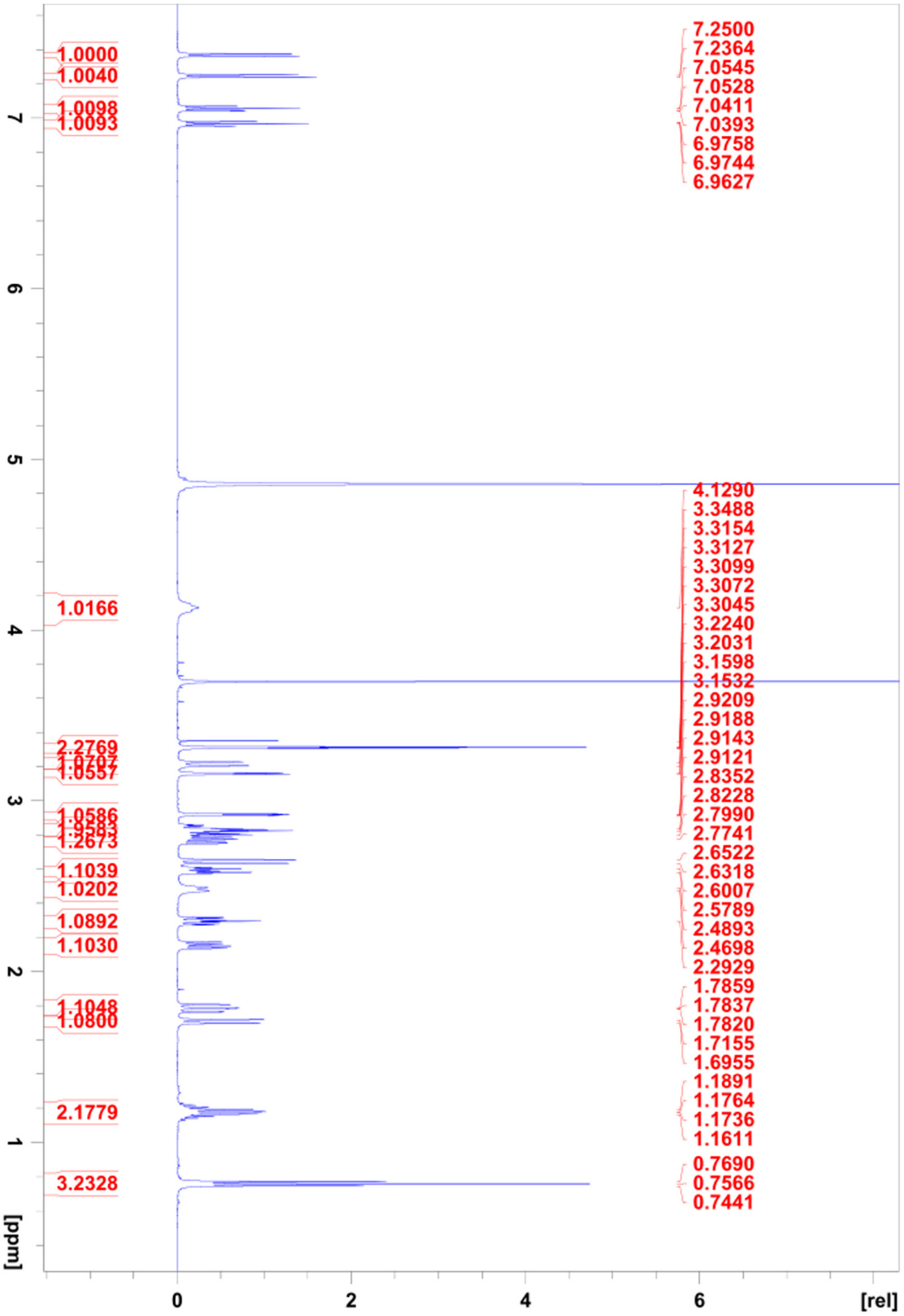


**Figure S61**: $^{1}$H NMR of voafinidine epoxide (**9**) in $CD_3OD$.

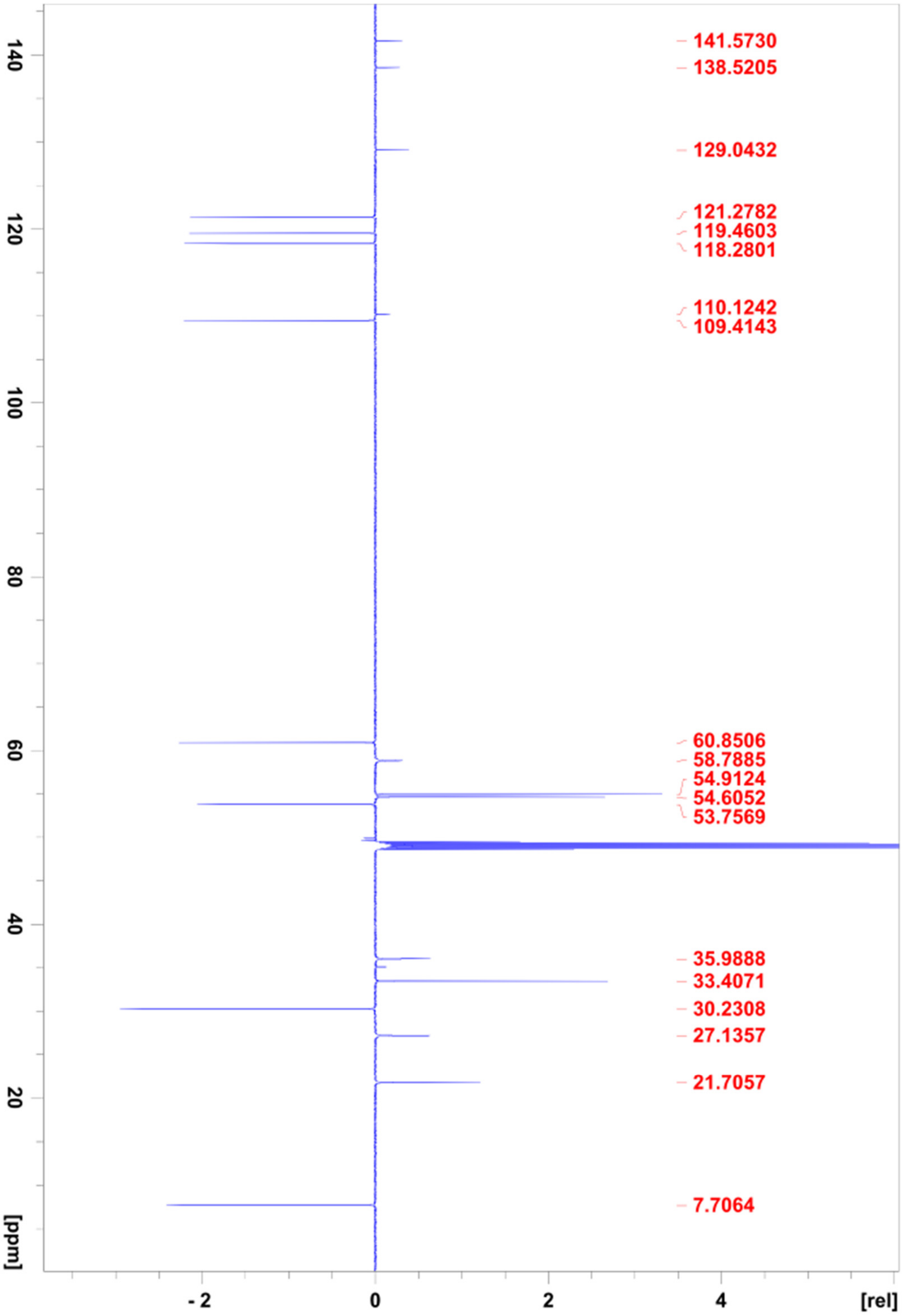


**Figure S62**: $^{13}C$ NMR of voafinidine epoxide (**9**) in $CD_3OD$.

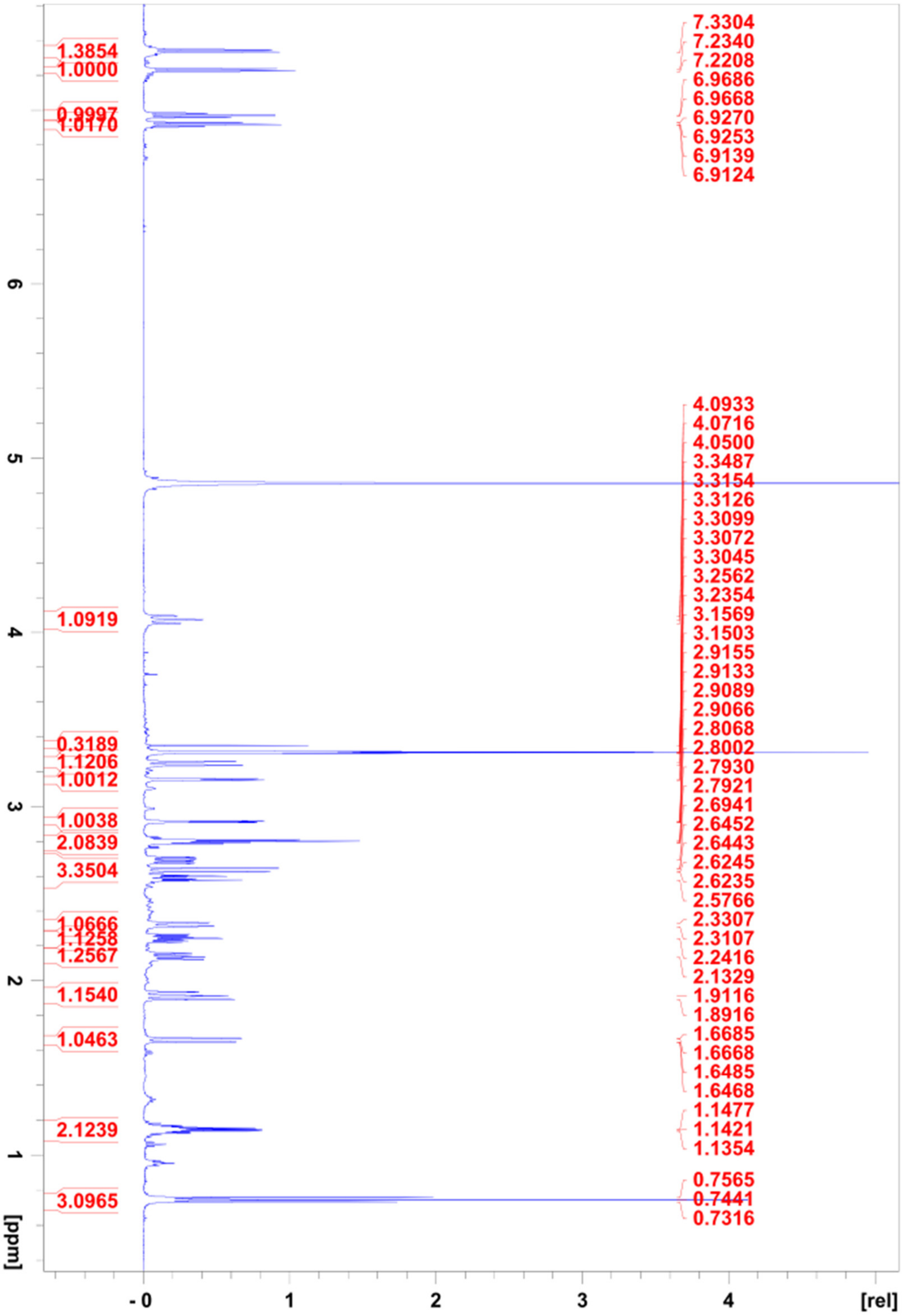


**Figure S63**: $^{1}$H NMR of voaphylline (**10**) in $CD_3OD$.

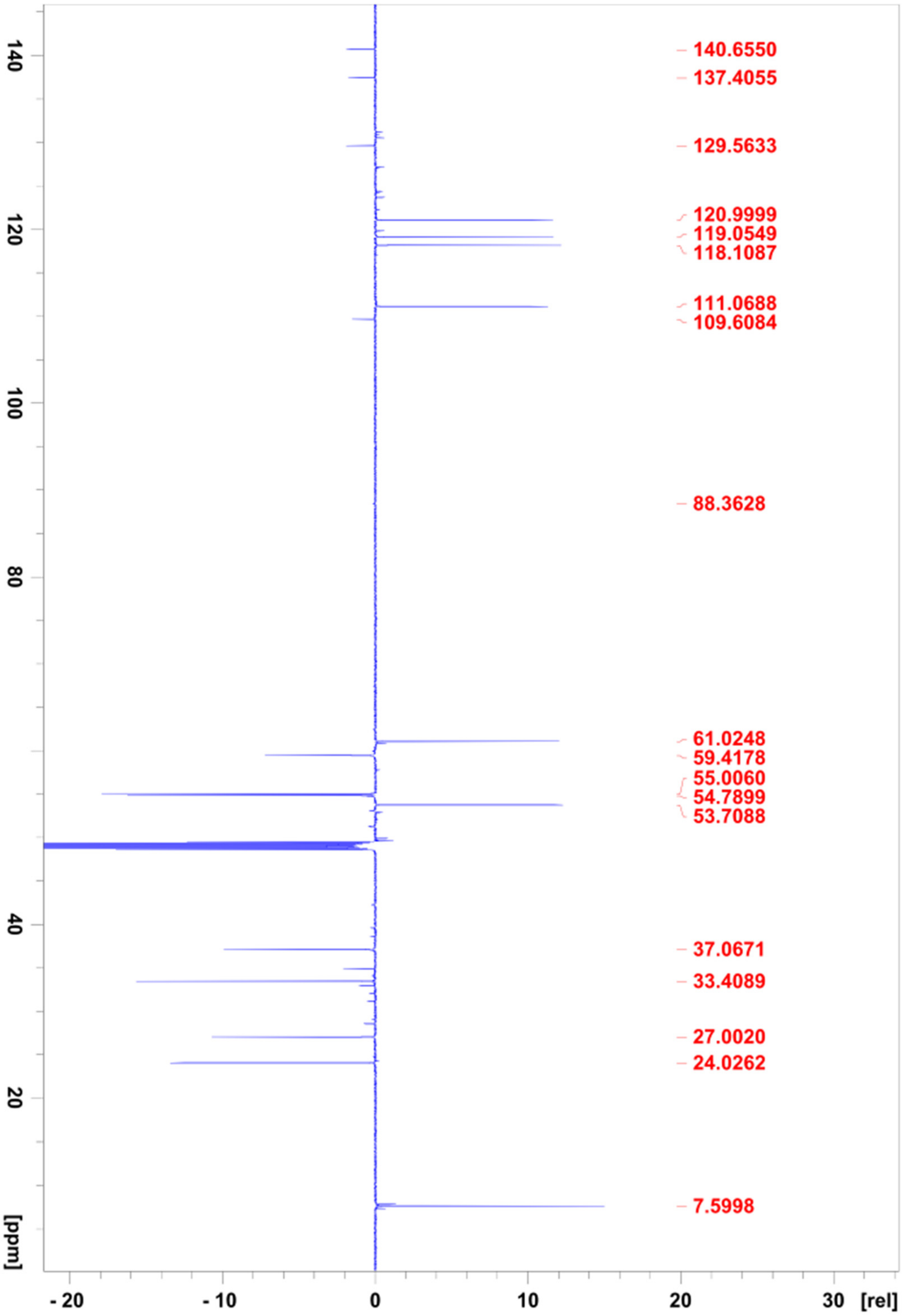


**Figure S64**: $^{13}C$ NMR of voaphylline (**10**) in $CD_3OD$.

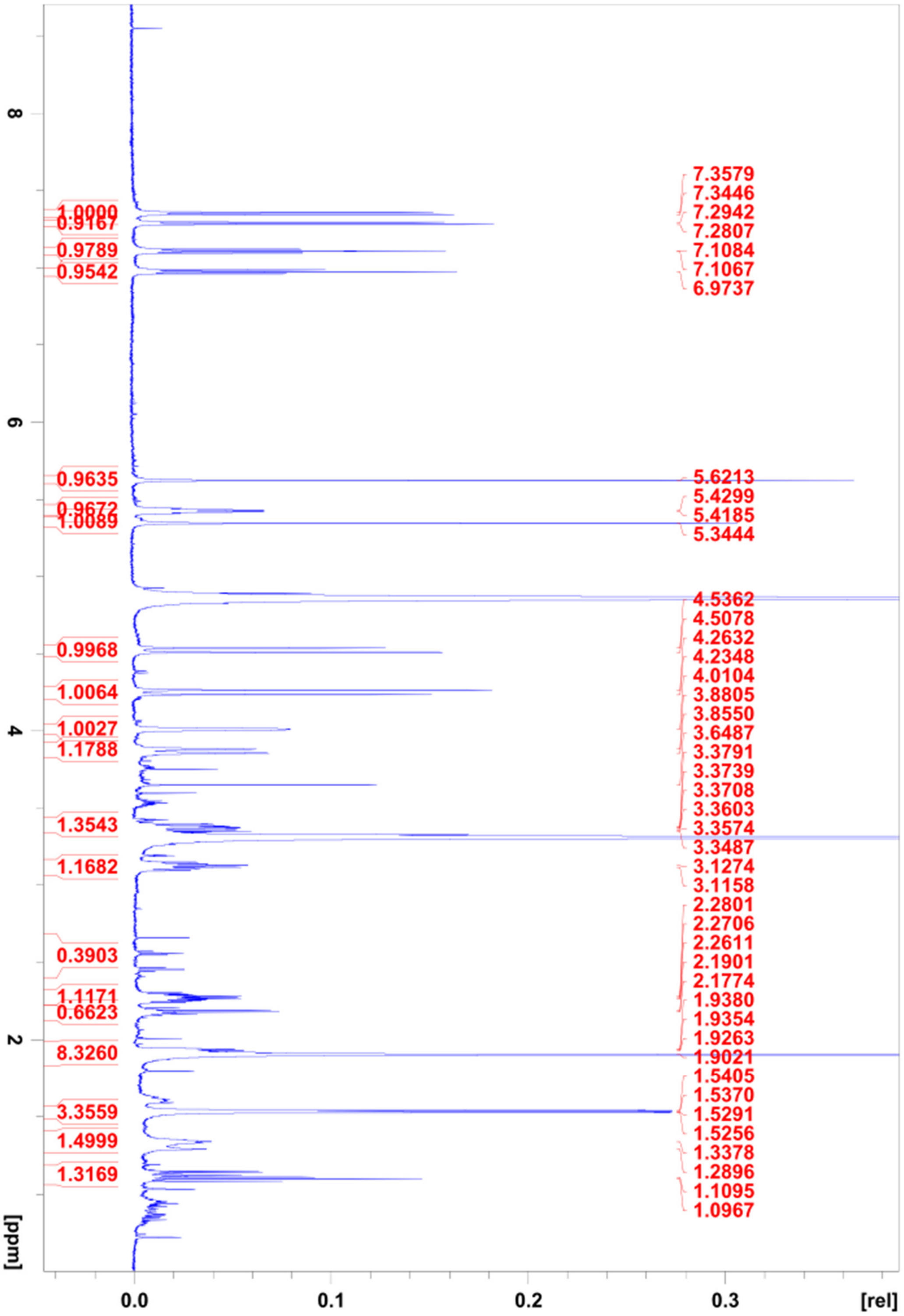


**Figure S65**: $^{1}$H NMR of apparicine (**11**) in $CD_3OD$.

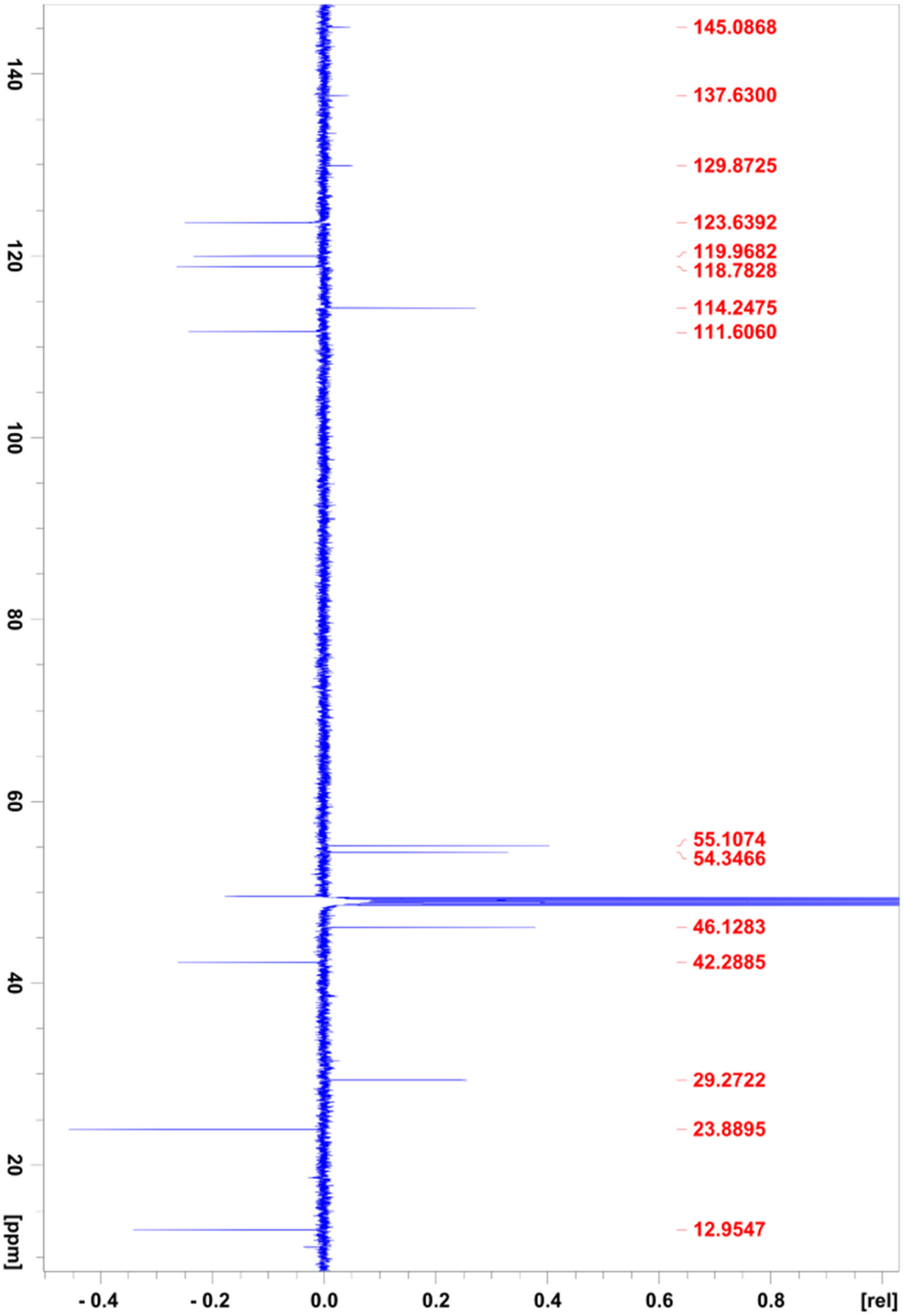


**Figure S66**: $^{13}C$ NMR of apparicine (**11**) in $CD_3OD$.

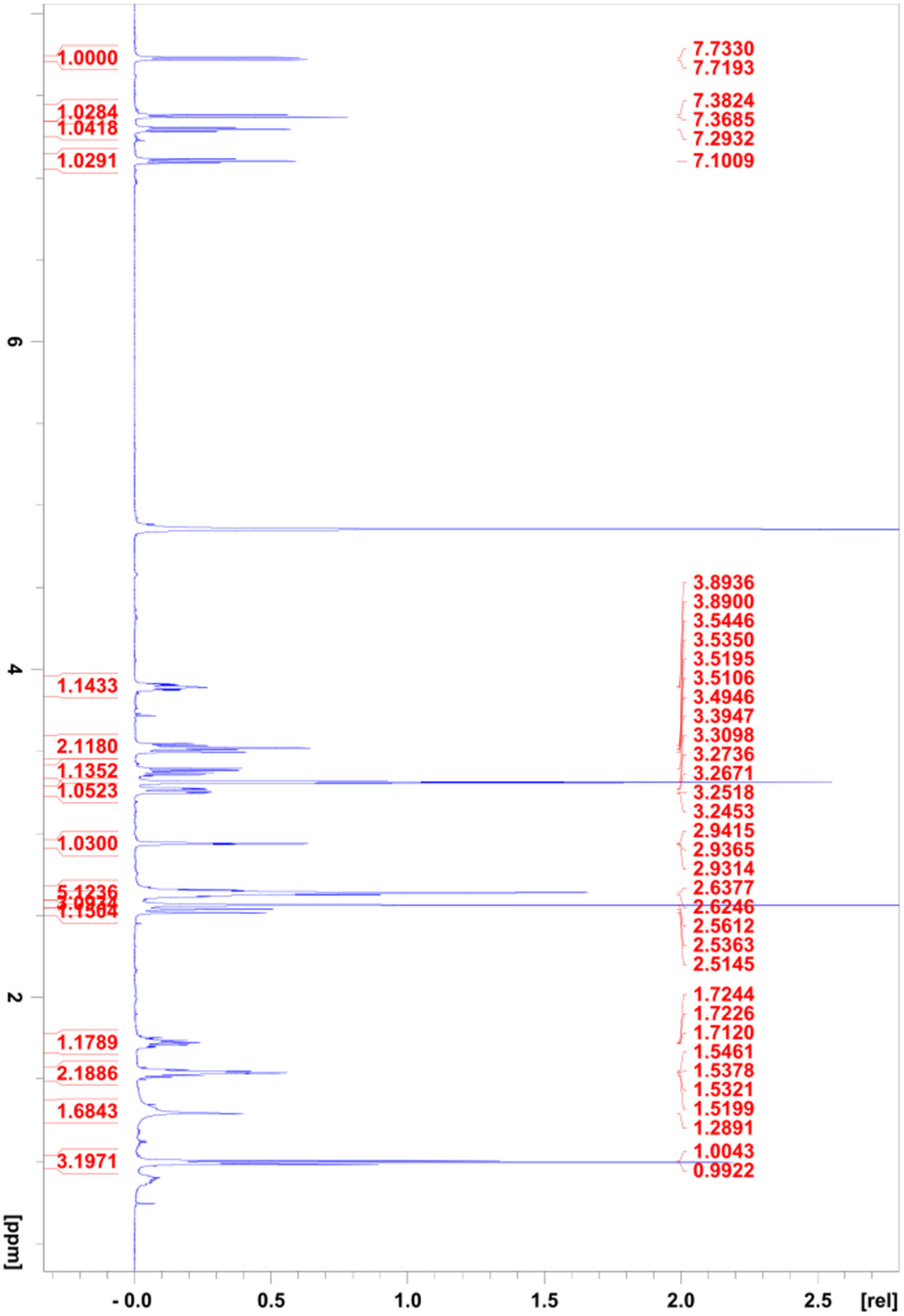


**Figure S67**: $^{1}$H NMR of tabernaemontanin (**12**) in $CD_3OD$.

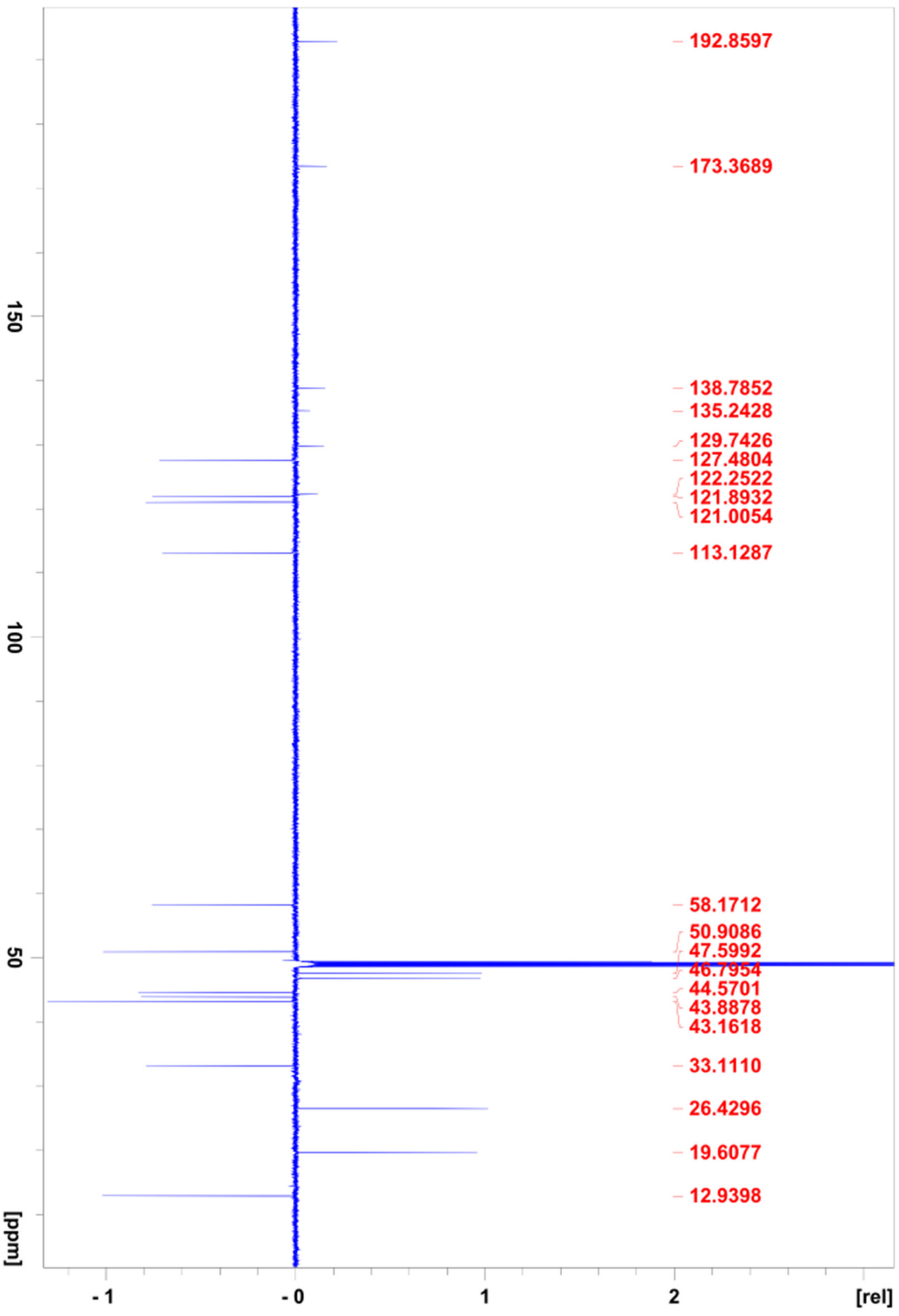


**Figure S68:** $^{13}C$ NMR of tabernaemontanin (**12**) in $CD_3OD$.

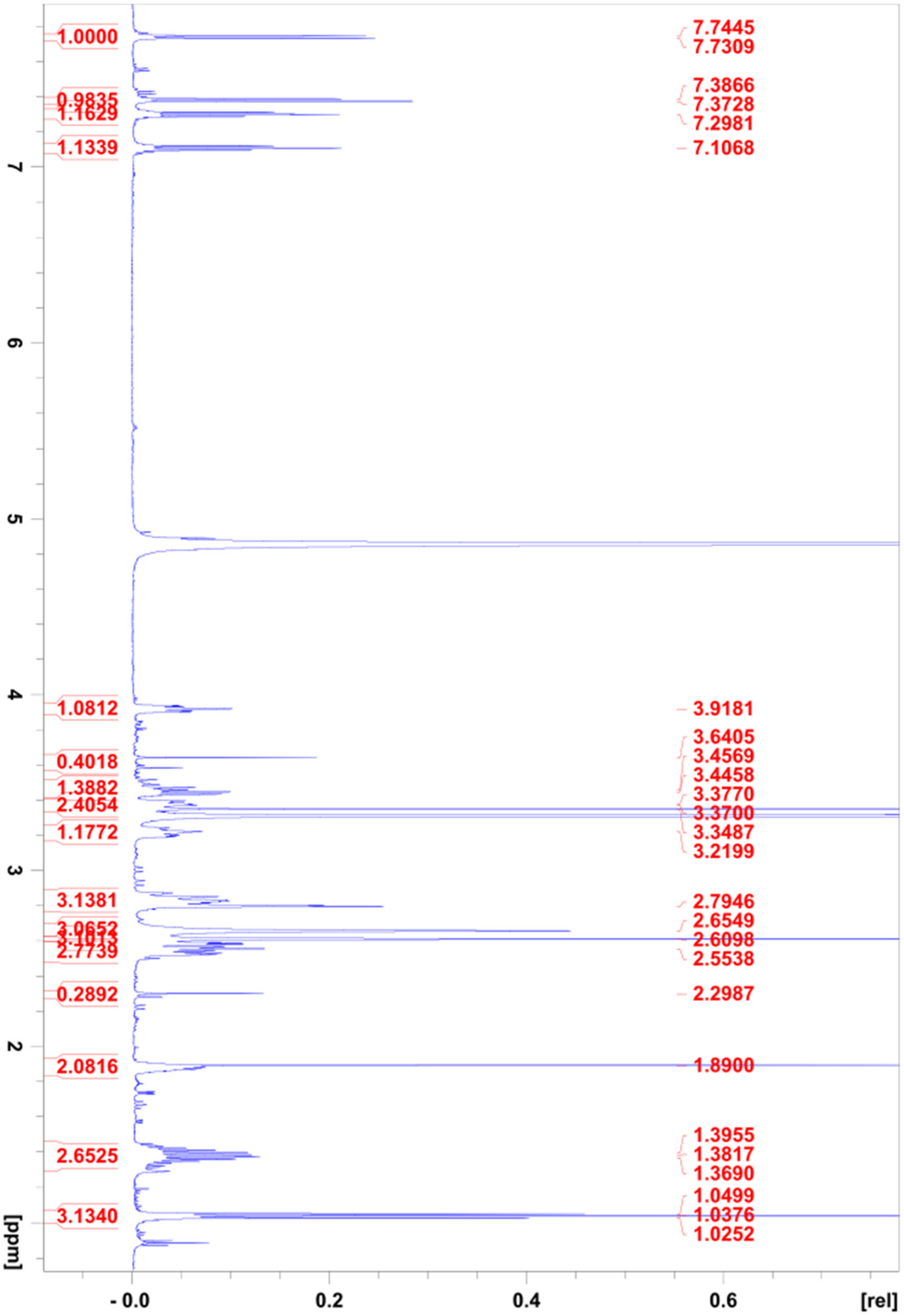


**Figure S69:** $^{1}$H NMR of dregamine (**13**) in $CD_3OD$.

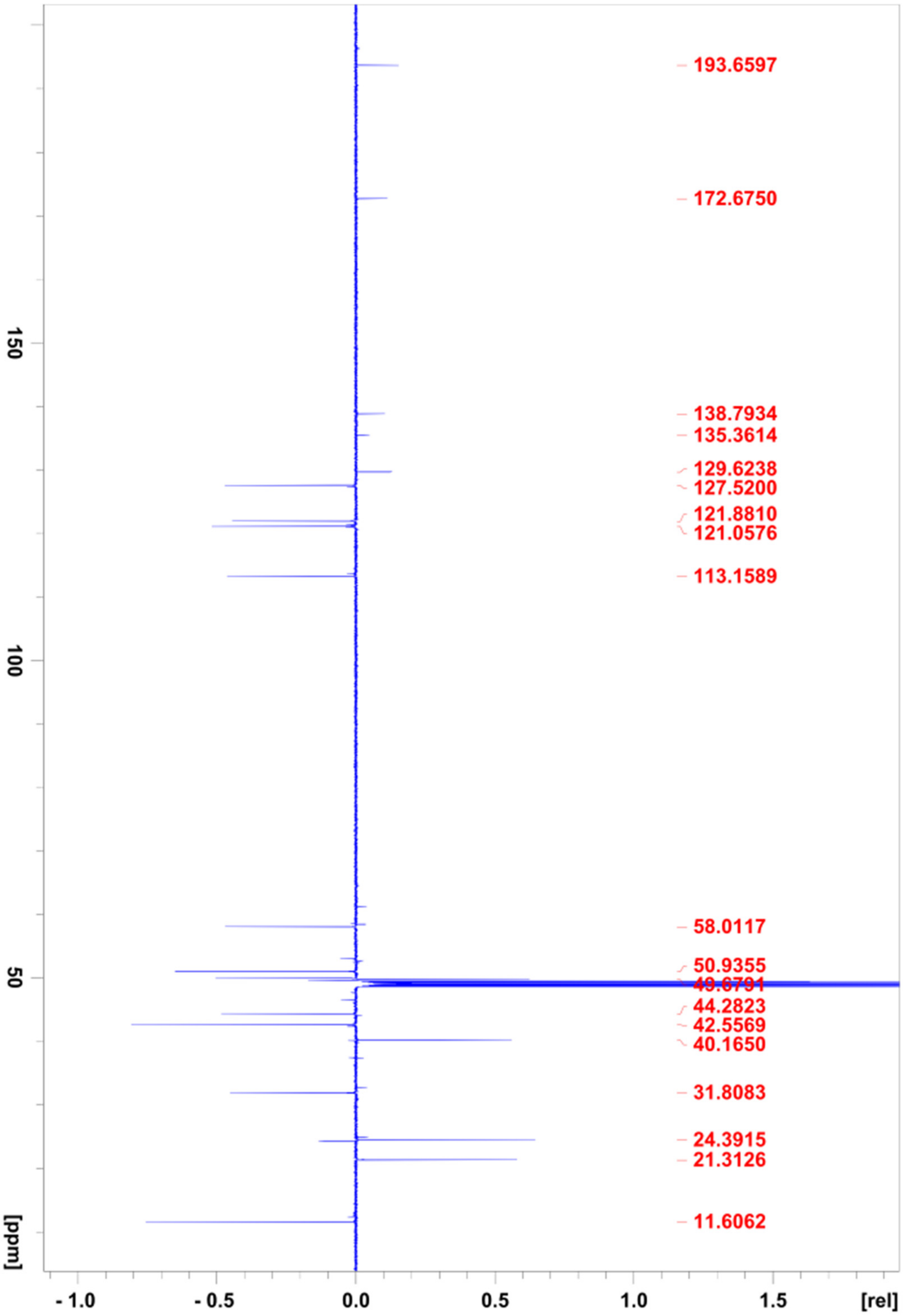


**Figure S70**: $^{13}C$ NMR of dregamine (**13**) in $CD_3OD$.

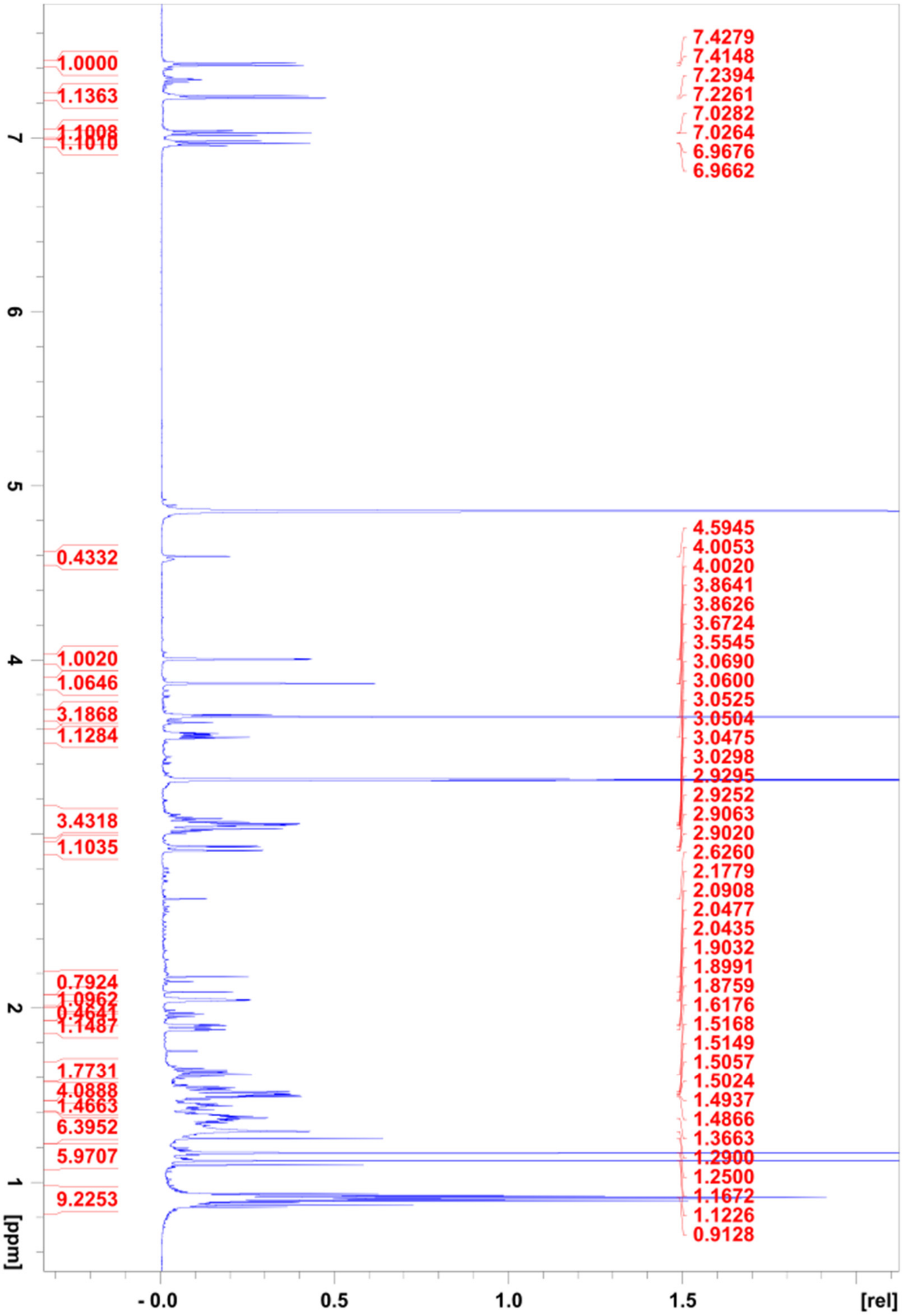


**Figure S71**: $^{1}$H NMR of 3-hydroxy-coronaridine (**14**) in $CD_3OD$.

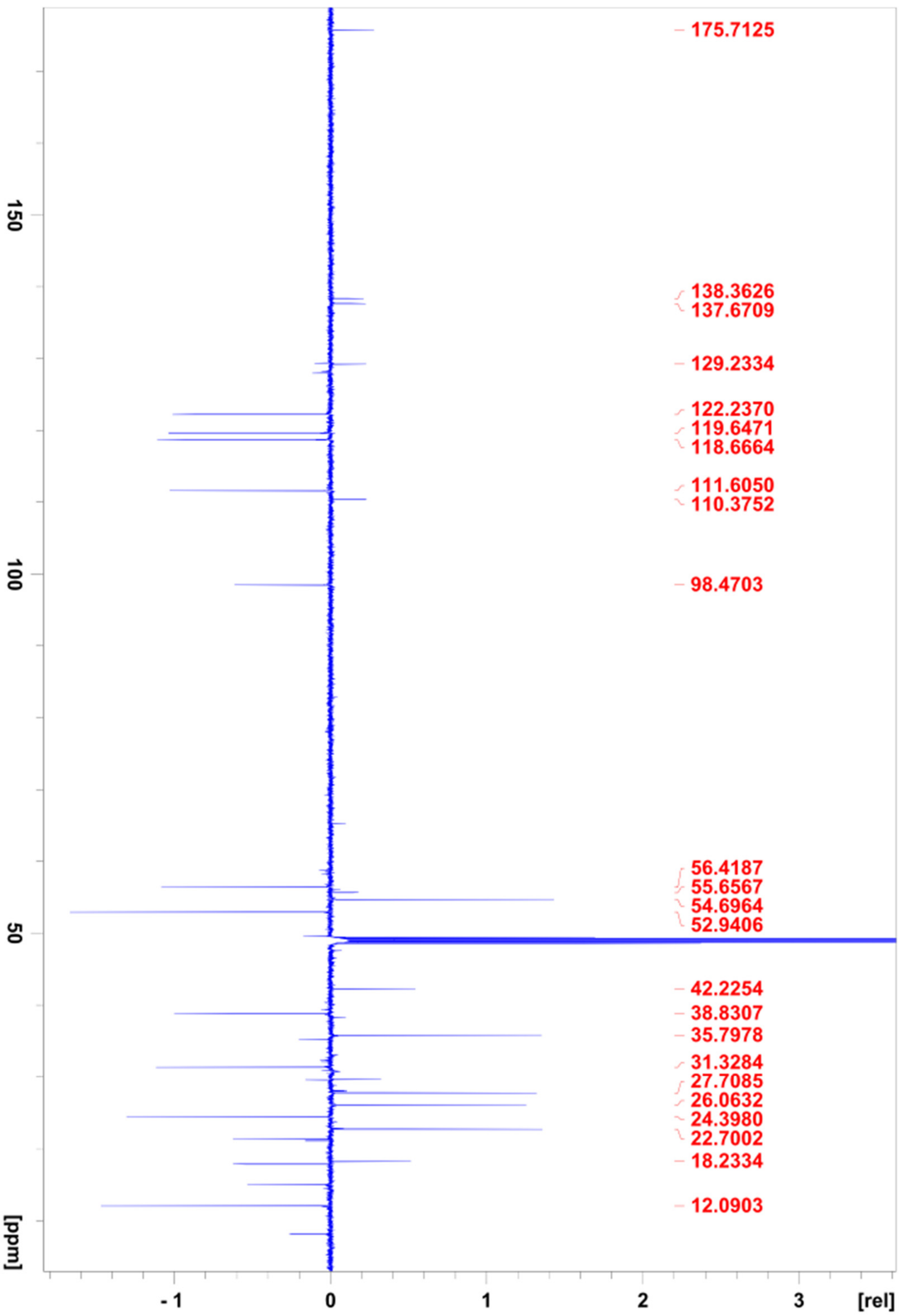


**Figure S72**: $^{13}C$ NMR of 3-hydroxy-coronaridine (**14**) in $CD_3OD$.

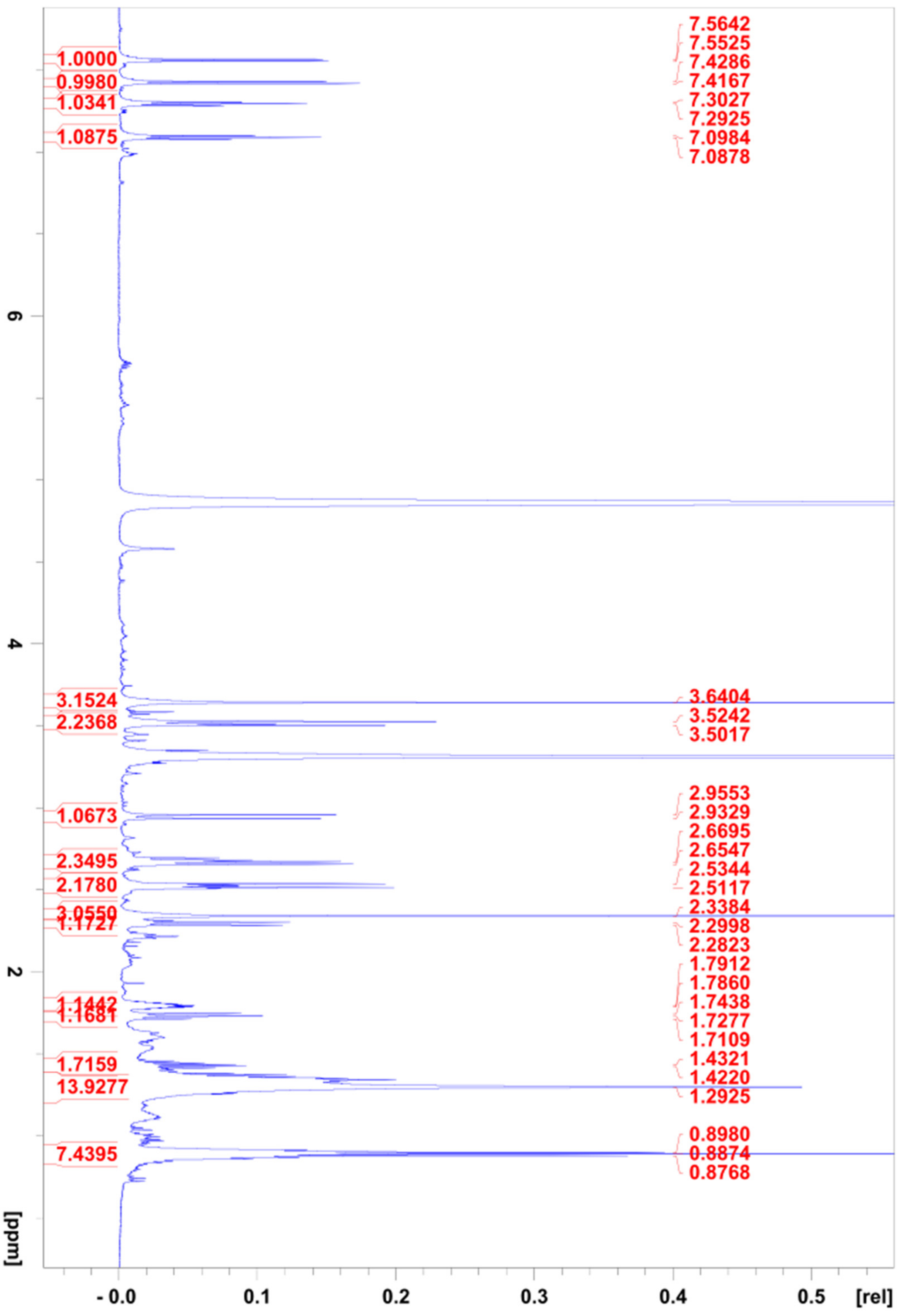


**Figure S73**: $^{1}$H NMR of ervatamine (**15**) in $CD_3OD$.

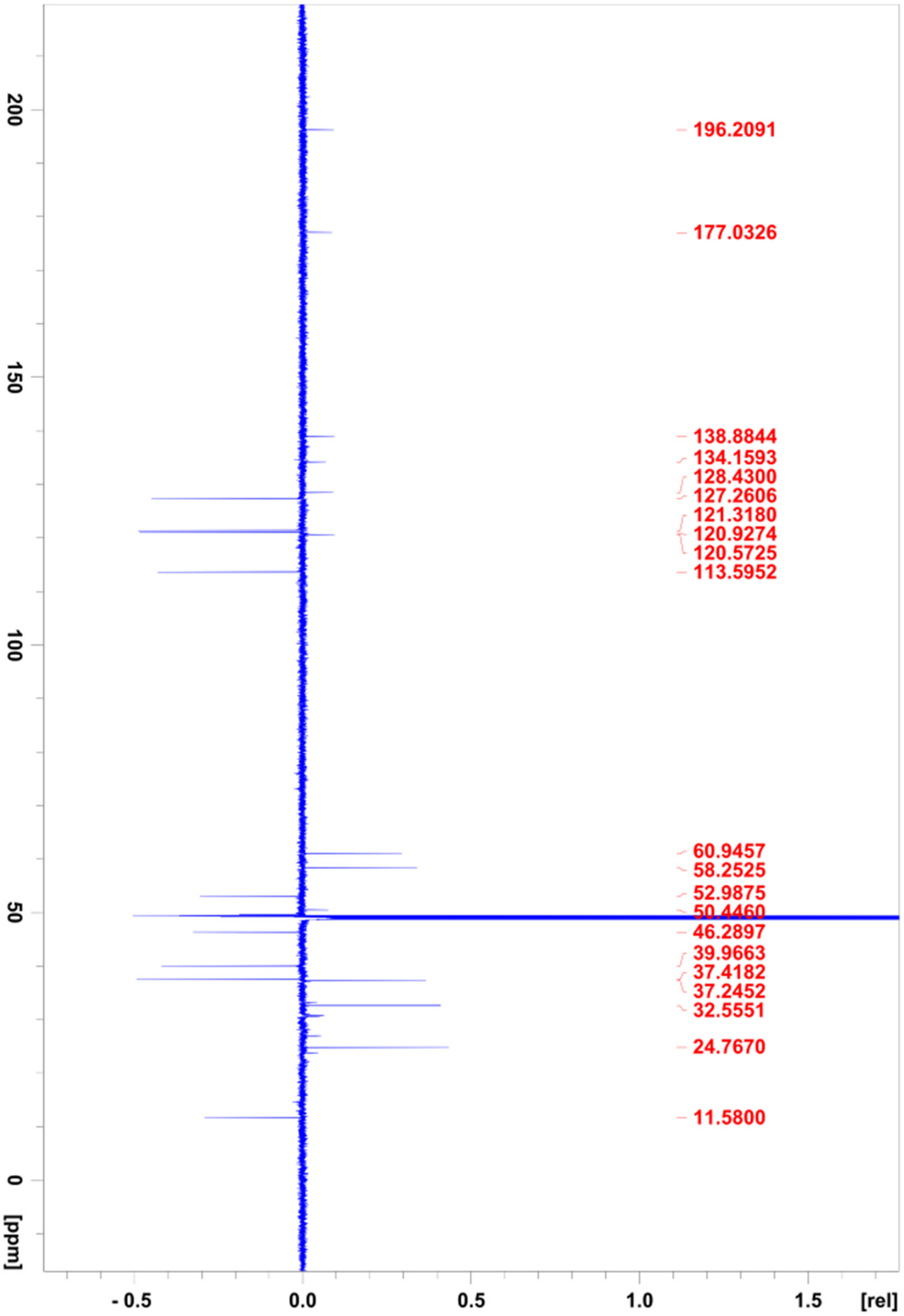


**Figure S74**: $^{13}$C NMR of ervatamine (**15**) in $CD_3OD$.

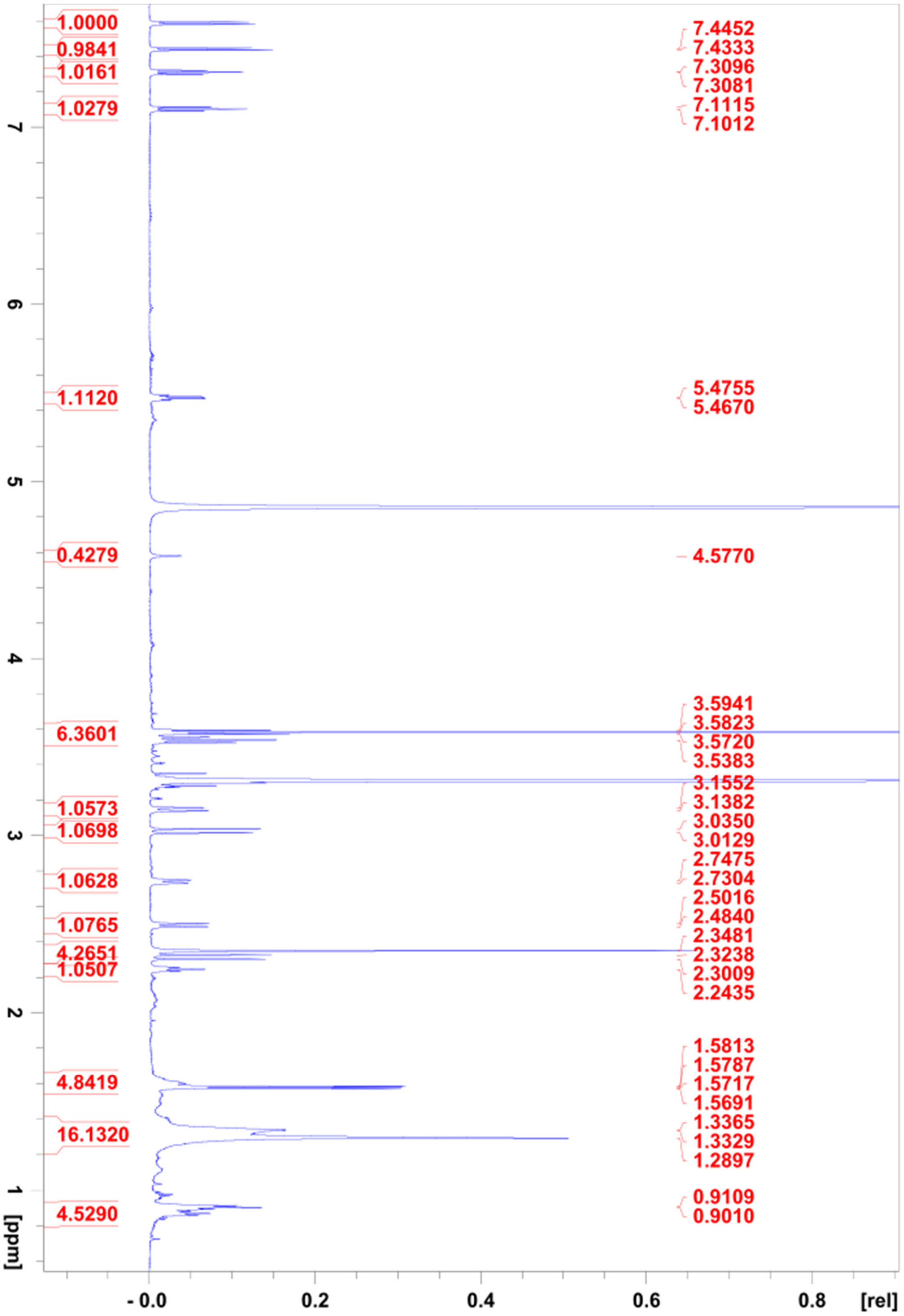


**Figure S75**: $^{1}$H NMR of 19,20-didehydro ervatamine (**16**) in $CD_3OD$.

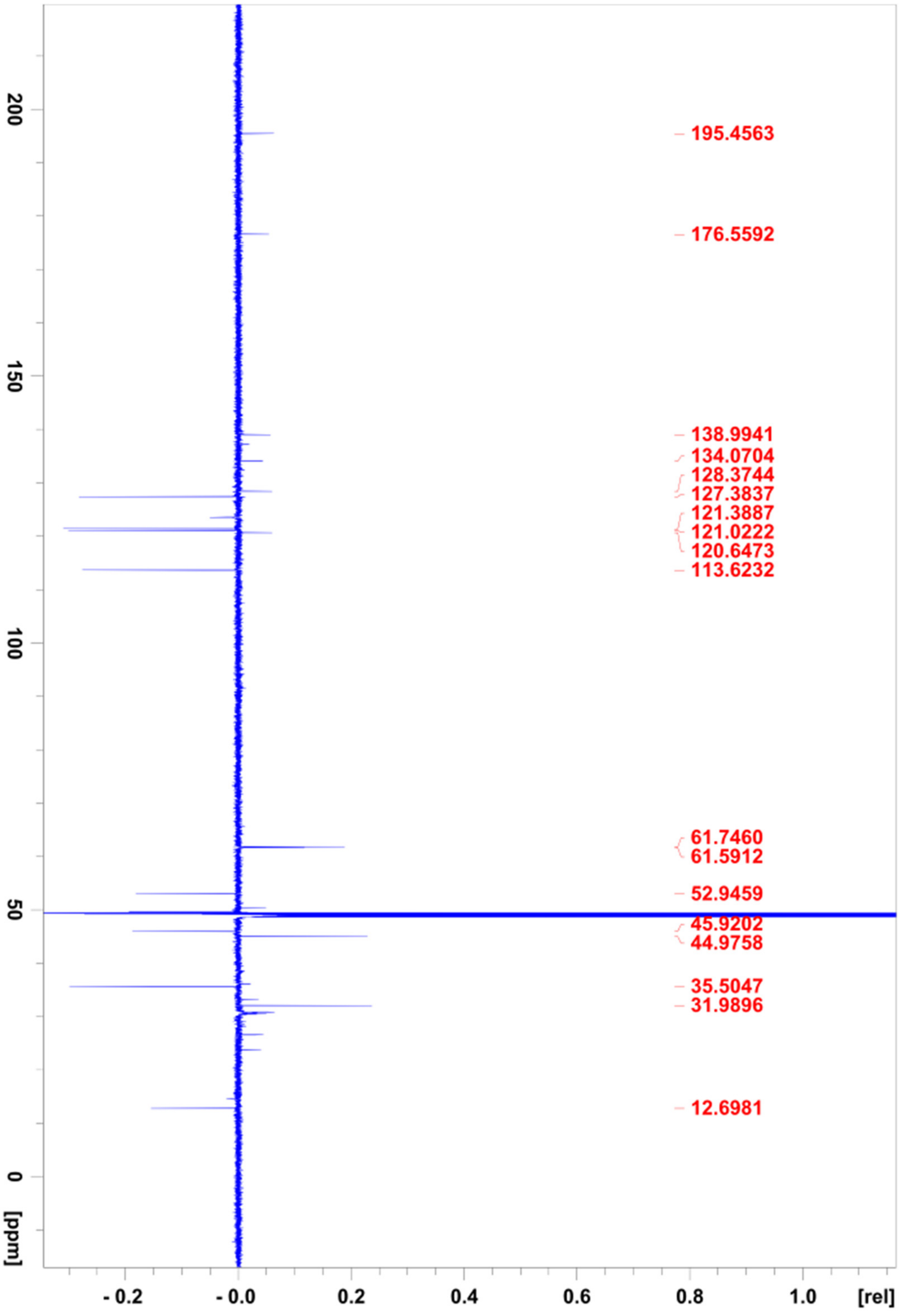


**Figure S76**: $^{13}C$ NMR of 19,20-didehydro ervatamine (**16**) in $CD_3OD$.

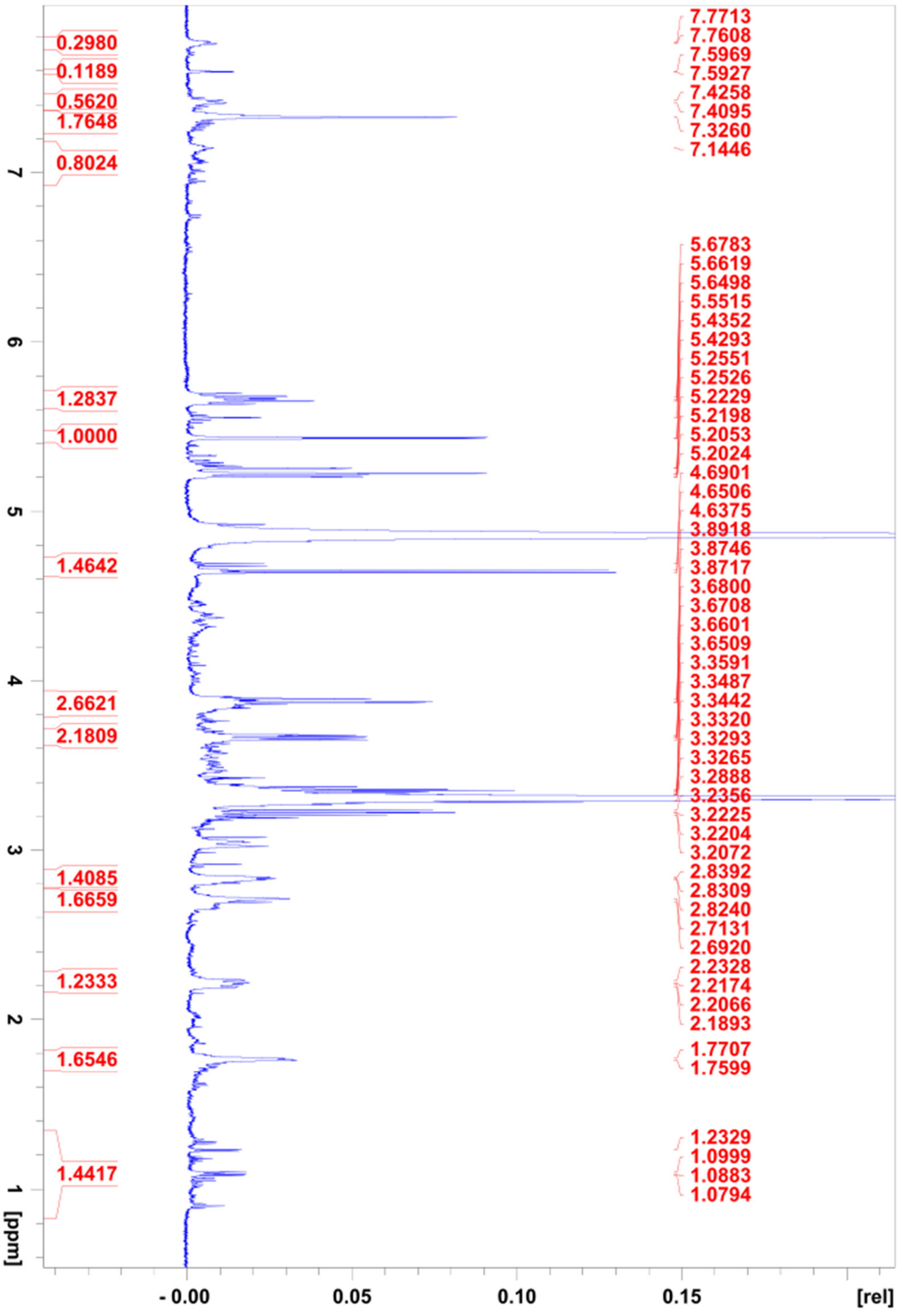


**Figure S77:** $^{1}$H NMR of secologanoside (**17**) in $CD_3OD$.

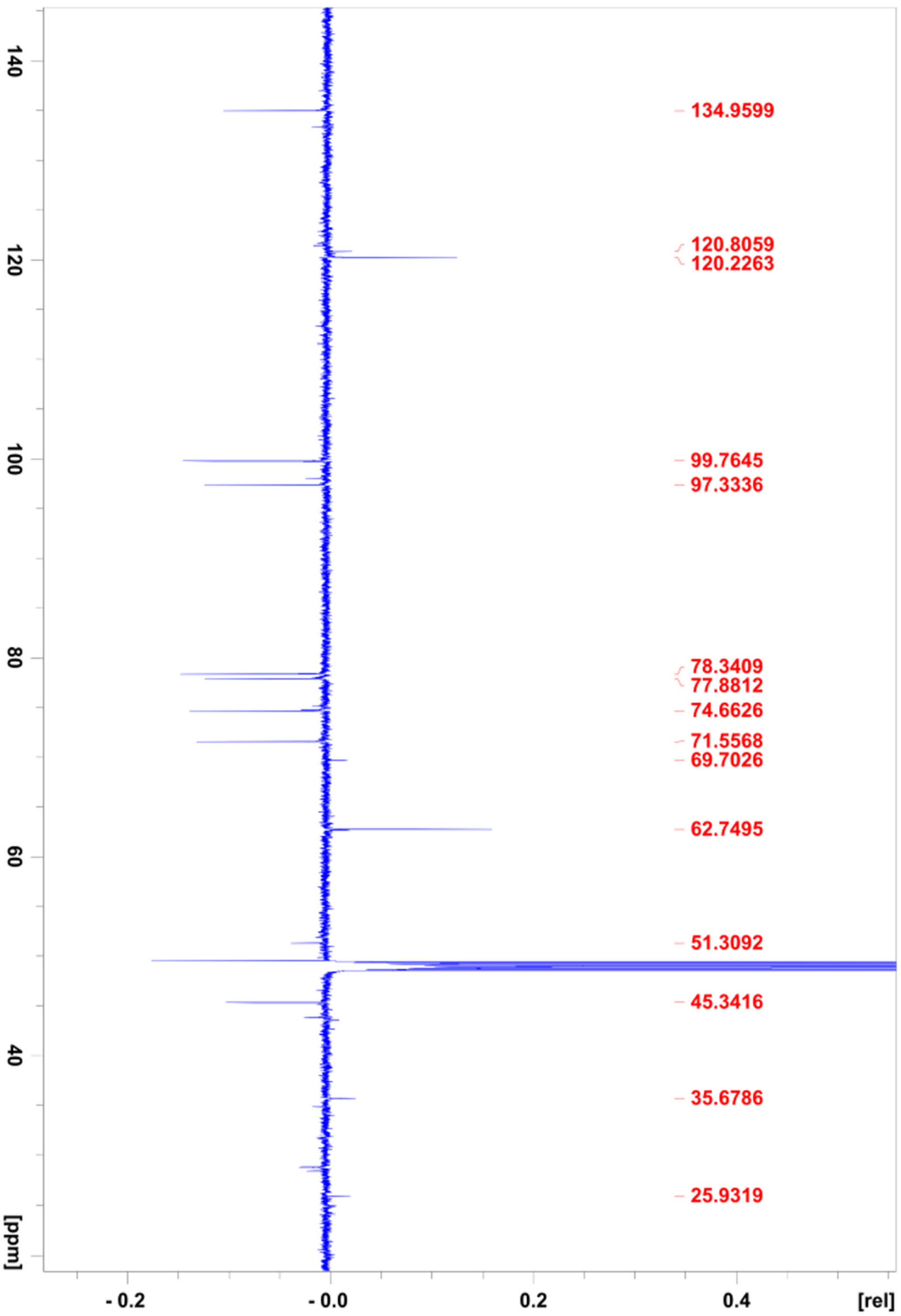


**Figure S78**: $^{13}$C NMR of secologanoside (**17**) in $CD_3OD$.

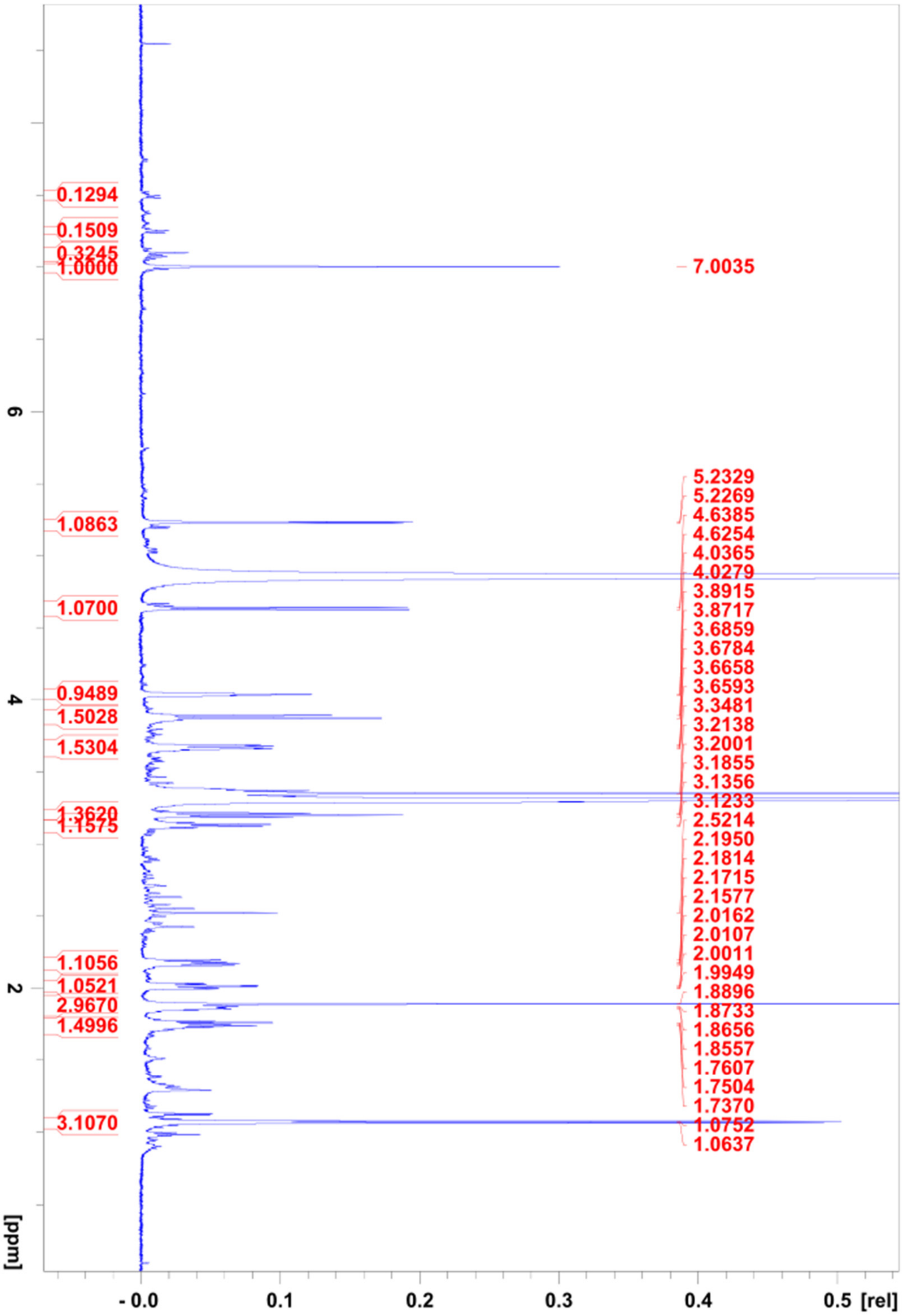


**Figure S79**: $^{1}$H NMR of loganic acid (**18**) in $CD_3OD$.

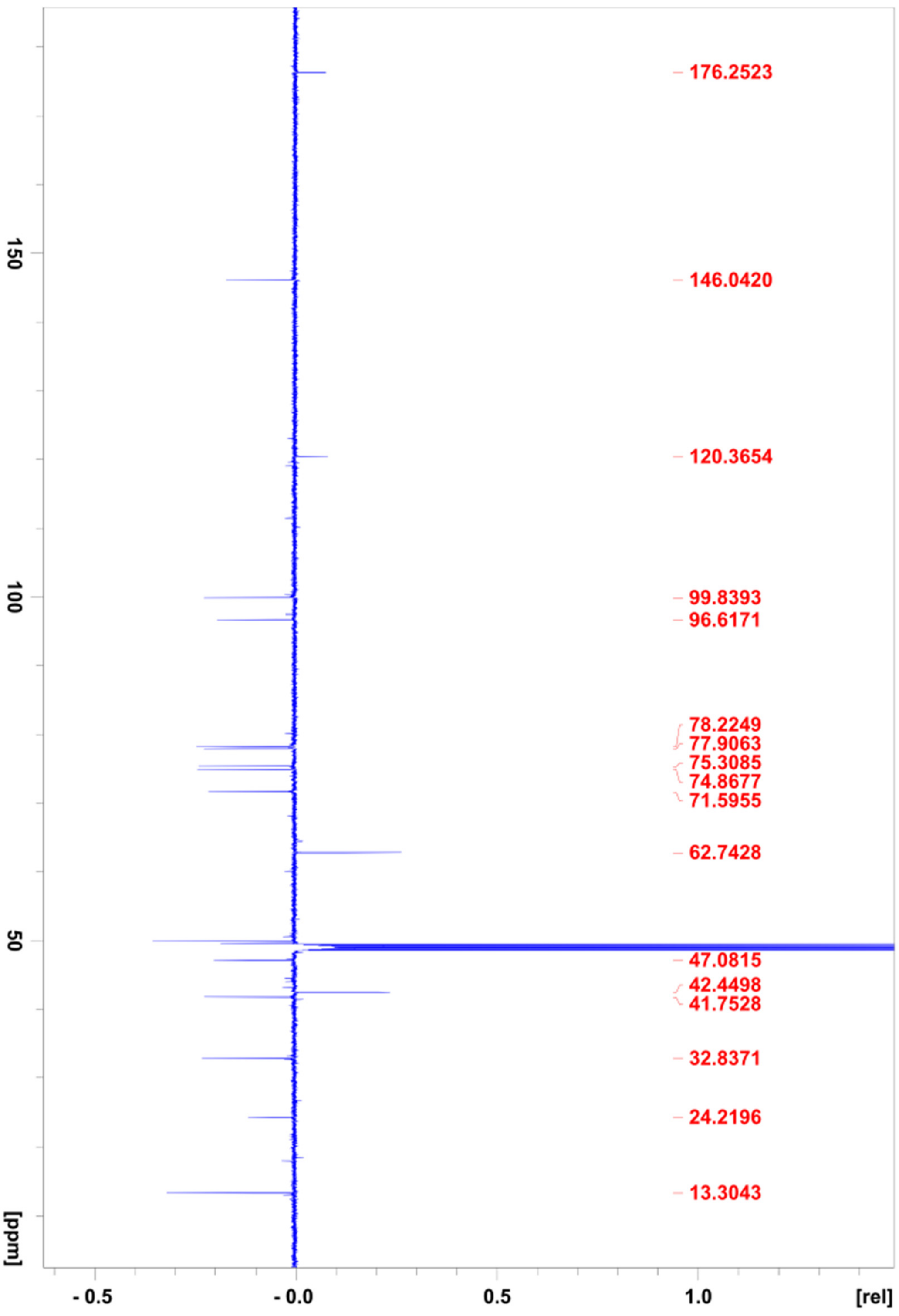


***Figure S80***: *$^{13}$C NMR of loganic acid (**18**) in $CD_3OD$.*

# 7. HPLC profiles of the isolated alkaloids

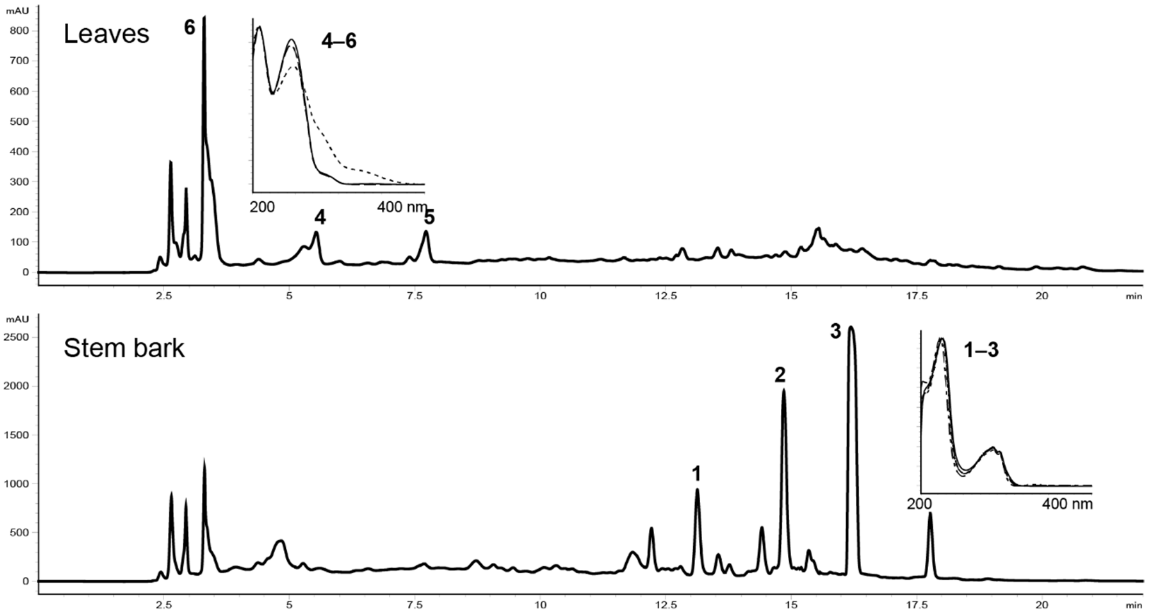


***Figure S81~~81~~:*** HPLC profiles of the leaf and stem bark extract of *T. penducularis*. **1**–**3** = coronaridine derivatives; **4**–**6** = javaniside derivatives.

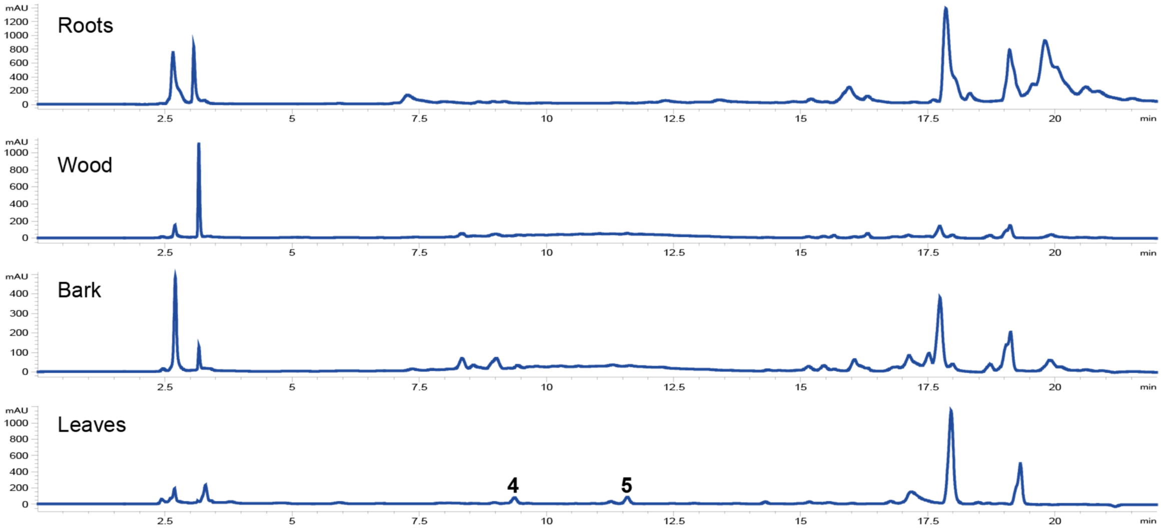


***Figure S82****:* HPLC profiles of the studied organs of *T. divaricvata.* **4** = javaniside, **5** = 7-epi javaniside. The alkaloid aglycones are present from 15 min onwards.
